# Anti-Inflammatory and Proangiogenic Metabolites from the Hadal Trench-Derived Fungus *Acremonium dichromosporum* YP-213

**DOI:** 10.3390/md22010025

**Published:** 2023-12-29

**Authors:** Yan Zhang, Jia-Bao Zhou, Shu-Ting Yang, Xin Liu, Wei Cao, Pei-Hai Li, Hao Chen, Ya-Qin Fan

**Affiliations:** 1 Shandong Provincial Key Laboratory of Applied Mycology, School of Life Sciences, Qingdao Agricultural University, Qingdao 266109, China; 20212206060@stu.qau.edu.cn (Y.Z.); zhoujiabao1224@163.com (J.-B.Z.); yangshuting0701@163.com (S.-T.Y.); liuxin202312@163.com (X.L.); caowei579@163.com (W.C.); 2 Engineering Research Center of Zebrafish Models for Human Diseases and Drug Screening of Shandong Province, Shandong Provincial Engineering Laboratory for Biological Testing Technology, Key Laboratory for Biosensor of Shandong Province, Biology Institute, Qilu University of Technology (Shandong Academy of Sciences), Jinan 250103, China; liph@sdas.org; 3 MNR Key Laboratory of Marine Eco-Environmental Science and Technology, First Institute of Oceanography, Ministry of Natural Resources, Qingdao 266061, China; hchen@fio.org.cn

**Keywords:** hadal trench, *Acremonium dichromosporum*, ascochlorin-type meroterpenoid, pyridone alkaloid, cyclopentenone derivative, anti-inflammatory activity, proangiogenic activity

## Abstract

Four new compounds, including two ascochlorin-type meroterpenoids acremocholrins A (**1**) and B (**2**), one pyridone alkaloid acremopyridone A (**7**), and one cyclopentenone derivative acremoketene A (**12**), together with eight known compounds (**3**–**6** and **8**–**11**), were isolated and identified from the hadal trench-derived fungus *Acremonium dichromosporum* YP-213. Their structures were determined with a detailed spectroscopic analysis of NMR and MS data, NOE analysis, octant rule and quantum chemical calculations of ECD, and NMR (with DP4+ probability analysis). Among the compounds, **7** represent a novel scaffold derived from a pyridone alkaloid by cleavage of the C-16-C-17 bond following oxidation to give a ketone. Compounds **9**, **11**, and **12** showed potent in vivo anti-inflammatory activity in transgenic zebrafish, while compound **8** exhibited significant proangiogenic activity in transgenic zebrafish.

## 1. Introduction

The hadal trench, consisting of deep-sea trenches deeper than 6000 m, represents one of the most unique habitats in the deep sea, characterized by extreme high pressure, low temperature, geological isolation, complex topography, and high seismic activity [1]. The steep slopes of the trenches, formed by the funnel effect of the V-shaped narrow troughs created by plate subduction, transport organic particles from the upper layers downwards, leading to the accumulation of benthic elements in the hadal zone. The hadal trench harbors a diverse range of large benthic organisms, as well as a rich microbial community with unique biodiversity and species specificity [2]. Under extreme conditions, hadal fungi have gradually evolved physiological adaptations, genetic mechanisms, and metabolic systems that allow them to produce and accumulate secondary metabolites distinct from those of terrestrial and shallow-sea microorganisms. Currently, research on natural products from hadal microorganisms is still scarce, and the exploration and utilization of hadal microbial metabolites are lagging behind. However, the potential for discovering novel drug leads from hadal microorganisms is enormous.

**Figure 1:**
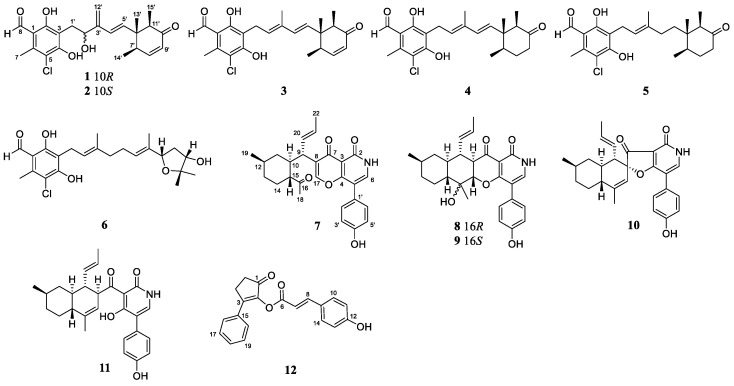
Structures of compounds **1**–**12**.

In the course of discovering bioactive metabolites from hadal trench-derived fungi [3,4], the fungal strain *Acremonium dichromosporum* YP-213, which was isolated from a seawater sample collected from Yap Trench in the Pacific Ocean, drew our attention. Chemical investigation of this fungus led to the isolation of four new compounds, including two ascochlorin-type meroterpenoids acremocholrins A (**1**) and B (**2**), one pyridone alkaloid acremopyridone A (**7**), and one cyclopentenone derivative acremoketene A (**12**), together with eight known compounds, 8′-9′-dehydroascochlorin (**3**), LL-Z 1272 γ (**4**), LL-Z 1272 δ (**5**) [5], ascofuranol (**6**) [6], campyridones A (**10**), C (**8**), and D (**9**) [7] and ilicicolin H (**11**) [8], have been isolated and identified (Figure 1). Among these compounds, **7** represents a novel scaffold derived from a pyridone alkaloid by cleavage of the C-16-C-17 bond following oxidation to give a ketone. Details of the isolation and purification, structure elucidation, and biological evaluation of compounds **1**–**12** are described herein.

## 2. Results and Discussion

### 2.1. Structure Elucidation

**Figure 2:**
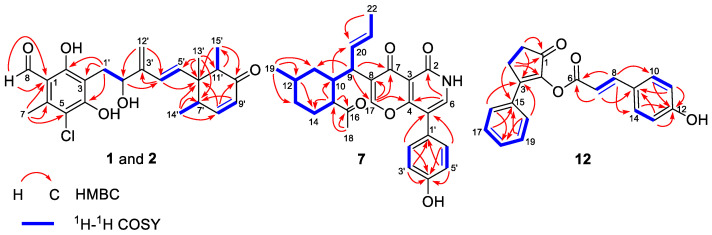
Key ^1^H-^1^H COSY (bold blue lines) and HMBC (red arrows) correlations of compounds **1**, **2**, **7**, and **12**.

Acremocholrin A (**1**) was obtained as a pale yellow amorphous powder. The molecular formula of **1** was determined to be C_23_H_27_ClO_5_ using HRESIMS (Figure S2), indicating ten degrees of unsaturation. Specifically, the existence of a chlorine group was further deduced using the isotopic peaks at *m*/*z* 441 and 443 with a ratio of 3:1. The ^1^H NMR data (Table 1) and HSQC spectrum displayed signals for an aldehyde proton (H-8), six aromatic/olefinic protons (H-4′, H-5′, H-8′, H-9′, and H_2_-12′), an oxygenated methine proton (H-2′), and four methyls (H_3_-7, H_3_-13′, H_3_-14′, H_3_-15′). The ^13^C NMR data (Table 1) revealed the presence of 23 carbon signals, sorted using DEPT into four methyls, two methylenes (including one olefinic), seven methines (including four olefinic), and nine quarternary carbons (including seven aromatic/olefinic and one conjugated keto). The general features of the ^1^H and ^13^C NMR data of **1** resembled 8′,9′-dehydroascochlorin [5], a previously reported analogue isolated from the cultural mycelium of *Verticillium* sp. FO-2787. The major difference was that the signals of the olefinic methine (CH-2′) resonating at *δ*_H/C_ 5.55/128.0 and of the methyl (3′-Me) resonating at *δ*_H/C_ 1.93/12.7 in the NMR spectra of 8′,9′-dehydroascochlorin were replaced by an oxygenated sp^3^ methine resonating at *δ*_H/C_ 4.61/69.9 and an olefinic methylene resonating at *δ*_H/C_ 5.25, 5.12/148.2 in those of **1**, respectively. The above observation suggested that, in comparison to 8′,9′-dehydroascochlorin, compound **1** has undergone a hydroxylation of C-2′ and a dehydrogenation of C-12′, which led to the rearrangement of Δ^2′^ double bond to Δ^3′^. This deduction was further verified using the key HMBC from H-2′ to C-4′, from H-4′ to C-3′, and from H-12′ to C-2′ and C-4′. The structure of **1** was fully defined using the HMBC correlations, as shown in Figure 2.

**Figure 3:**
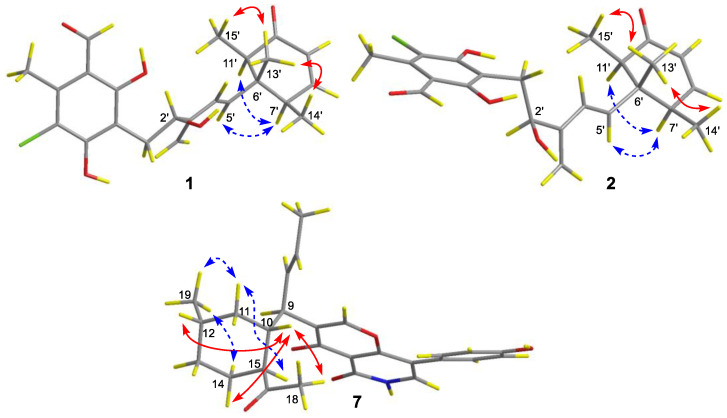
Key NOE correlations of compounds **1**, **2,** and **7** (red solid lines: α-orientation; blue dashed lines: β-orientation).

**Figure 4:**
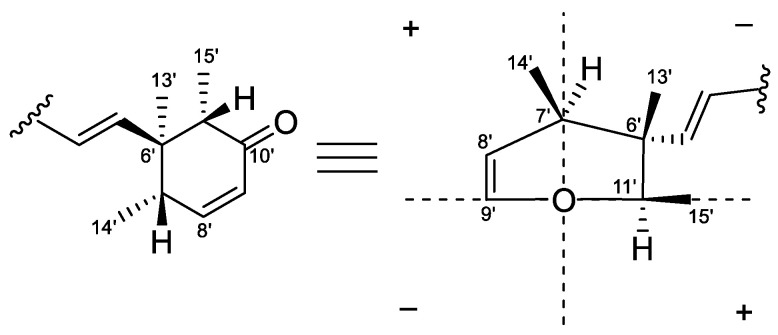
The octant rule for the cyclohexenone.

**Figure 5:**
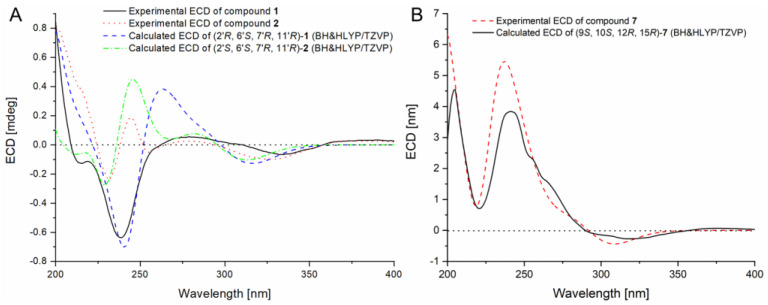
(**A**) Experimental and calculated ECD spectra of compounds **1** (sigma = 0.28 eV, UV-shift = +15 nm) and **2** (sigma = 0.28 eV, UV-shift = +15 nm). (**B**) Experimental and calculated ECD spectra of compound **7** (sigma = 0.28 eV, UV-shift = +15 nm). The calculations were carried out at the BH&HLYP/TZVP level.

The relative configuration of compound **1** was proposed after an analysis of NOE difference spectroscopy (Figure 3). NOE correlations from H-13′ to H-14′ and H-15′ revealed that these groups are on the same side, while correlations from H-7′ to H-5′ and H-11′ suggested these groups were on the other side of the molecule. The absolute configurations of C-6′, C-7′, and C-11′ were established using the octant rule for cyclohexenones [9]. The negative Cotton effect at 332 nm (Δ*ε*_max_ −0.2) for n→π* indicated the 6′*S*, 7′*R*, 11′*R* configuration (Figure 4). To further determine the whole absolute configuration of **1**, time-dependent, density functional (TDDFT)-ECD calculations at the BH&HLYP/TZVP level were performed. The calculated ECD spectrum for the (2′*R*, 6′*S*, 7′*R*, 11′*R*)-**1** matched well with that of the experimental curve (Figure 5A), allowing the establishment of the absolute configuration of **1** as 2′*R*, 6′*S*, 7′*R*, 11′*R* (Figure 1).

Acremocholrin B (**2**) was obtained as a pale yellow amorphous powder. Its molecular formula of C_23_H_27_ClO_5_ was determined using HRESIMS (Figure S9), which was the same as that of **1**. The NMR spectra of **2** was very similar to that of **1**, with some minor differences on the chemical shifts for H_3_-14′ and H_3_-15′. Inspection of the 1D NMR (Table 1) and NOESY data suggested that **2** is a diastereomer of **1**, epimeric at C-2′, which was further evidenced by the ECD calculations. The experimental ECD spectrum of **2** showed excellent accordance with that of (2′*S*, 6′*S*, 7′*R*, 11′*R*)-**2** (Figure 5A). Both experimental and calculated data showed positive CEs near 245 and 280 nm and negative CEs near 230 and 330 nm. These close similarities allowed assignment of the absolute configuration for **2** as shown (Figure 1).

Acremopyridone A (**7**) was obtained as a white amorphous powder. Its molecular formula, C_27_H_29_NO_5_ (Figure S16), was determined using HRESIMS data, indicating the presence of 14 degrees of unsaturation. In the ^1^H NMR spectrum of **7**, the signals indicative of a 1,4-disubstituted benzene ring system at *δ*_H_ 7.29 (d, *J* = 8.5 Hz, H-2′/6′) and *δ*_H_ 6.82 (d, *J* = 8.5 Hz, H-3′/5′) and of a *trans*-double bond at *δ*_H_ 5.66 (dd, *J* = 14.9, 10.7 Hz, H-20) and *δ*_H_ 5.37 (dd, *J* = 14.9, 6.6 Hz, H-21) were observed. The ^13^C NMR data, HSQC, and HMBC spectra (Table 2) indicated the presence of 26 carbon signals, which were sorted using DEPT and HSQC spectra into three methyls, three methylenes, twelve methines (including eight aromatic/olefinic), and nine quaternary carbons (including three carbonyls). The NMR data of **7** (Table 2) are very similar to those of campyridone C [7], a pyridine alkaloid isolated from a mangrove endophytic fungus, *Campylocarpon* sp. HDN13-307. However, signals for the methine group at C-8, the oxygenated quaternary carbon at C-16, and the oxygenated methine group at C-17 of campyridone C were absent in the NMR spectra of **7**. Instead, resonances for a trisubstituted-double bond at *δ*_C_ 129.0 (C-8) and *δ*_H_ 7.91/*δ*_C_ 151.7 (CH-17) and for a ketone group at *δ*_C_ 212.2 (C-16) were observed in the 1D and 2D NMR spectra of **7**. The above observation suggested that the C-16-C-17 bond in the structure of campyridone C was cleaved in that of **7**. Moreover, the formation of Δ^8^ and oxidation of C-16 were also observed in **7**. This deduction was further verified by the key HMBC correlations (Figure 2) from H-15 to C-10, C-14, and C-16, from H-17 to C-4, C-7, and C-8, and from H_3_-18 to C-15 and C-16. The structure of **7** was fully defined using the HMBC correlations, as shown in Figure 3.

**Figure 6:**
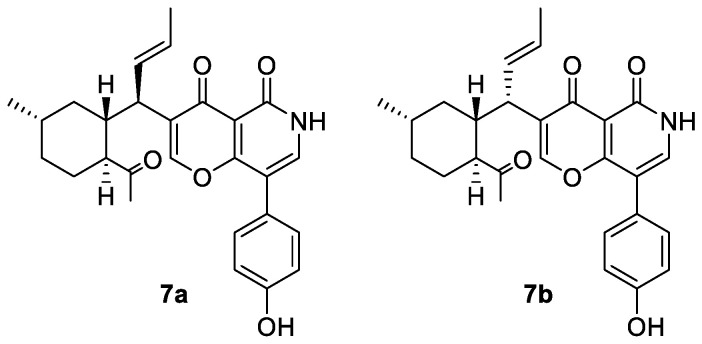
Structures of possible isomers for DP4+ probability analysis of compound **7**.

The relative configuration of **7** was partially assigned with an analysis of NOESY data (Figure 1). The key NOE correlations from H-10 to H-12, H-14β, and H_3_-18 suggested the *co*-facial orientation of these groups, while NOESY correlations from H-11α to H-15 and H_3_-19 and from H-14α to H_3_-19 placed these groups on the opposite face. The relative configuration of C-9 was determined with a comparison of the observed NMR data with those of computed values for two possible isomers (**7a** and **7b**, Figure 6) using DFT calculations through DP4+ probability analysis [10]. The experimental NMR data of **7** correspond to the computed NMR data for isomer **7a** with DP4+ probabilities of 100%, which led to the determination of the α-orientation of H-9. The absolute configuration of **7** was also determined using TDDFT-ECD calculation (Figure 5B). The experimental ECD spectrum of **7** matched well with that calculated for (9*R*, 10*R*, 12*S*, 15*S*)-**7** (Figure 5B). The structure and absolute configuration of **7** were thus assigned as shown in Figure 1.

Acremoketene A (**12**), obtained as a pale yellow amorphous powder, had a molecular formula C_20_H_16_O_4_ as determined with HRESIMS data (Figure S23), indicating 13 degrees of unsaturation. The signals in the ^1^H NMR spectrum (Table 3) of **12** for a *para*-substituted benzene ring (H-11/13 and H-10/14) and for a *mono*-substituted benzene ring (through H-16 to H-20) were observed. Meanwhile, protons for a *trans*-double bond (δ_H_ 6.63, d, *J* = 15.9 Hz, H-7; δ_H_ 7.75, d, *J* = 15.9 Hz, H-8) and for two methylenes (H_2_-4 and H_2_-5) were also found in the ^1^H NMR spectrum. The ^13^C NMR and DEPT data (Table 3) revealed the presence of two methylenes, eleven aromatic/olefinic methines, and seven quaternary carbons (including one ketone and one ester). The key HMBC correlations (Figure 2) from H_2_-4 to C-1 and C-2 and from H_2_-5 to C-1 and C-3, together with ^1^H-^1^H COSY correlations from H_2_-4 to H_2_-5, established the cyclopent-2-en-1-one moiety of **12**. The HMBC correlations from H-16/20 to C-3 and from H_2_-4 to C-15 indicated that the phenyl was attached to the cyclopent-2-en-1-one portion via C-3. The remaining part of the structure of **12** was determined as a cinnamoyl group connected to C-2 with an ester bond with the ^1^H-^1^H COSY correlations from H-7 to H-8 and from H-10/14 to H-11/13 along with the HMBC correlations from H-7 to C-6 and C-9, from H-8 to C-6 and C-10/14, from H-11/13 to C-9 and C-12, and from H-10/14 to C-9. Based on the above data, the structure of compound **12** was assigned as shown in Figure 1.

### 2.2. Bioactivity

**Figure 7:**
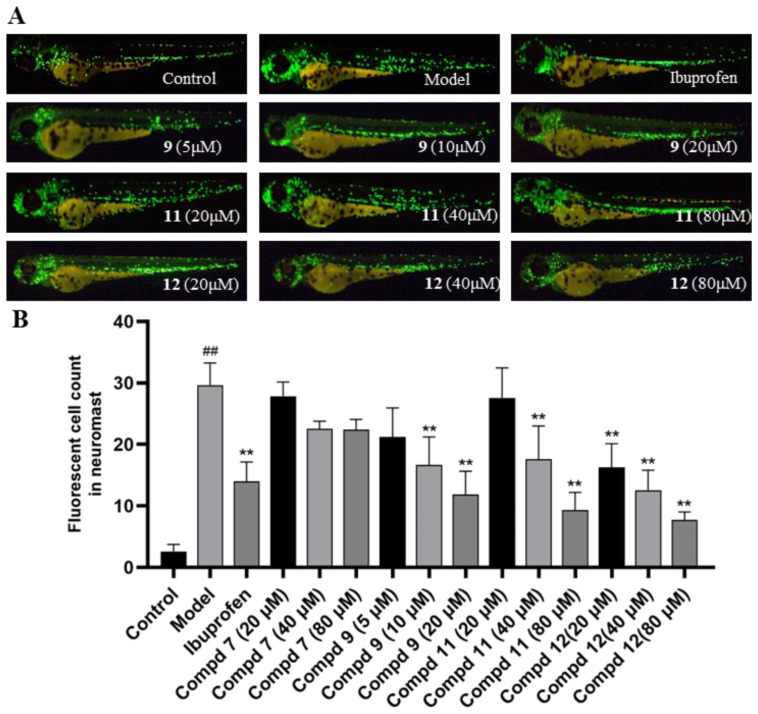
The anti-inflammatory activity of isolated compounds in Tg (zlyz-EGFP) zebrafish. The macrophages that migrated above the caudal notochord were numbered using an Olympus IX53 microscope. (**A**) Typical images of migratory fluorescent macrophages in transgenic zebrafish, using ibuprofen as a positive control. (**B**) Quantitative analysis of fluorescent macrophages in transgenic zebrafish (*n* = 10, mean ± SEM). ^##^ *p* < 0.01, compared to the control group. ** *p* < 0.01 compared to the model group.

The isolated compounds were evaluated for in vivo anti-inflammatory activity in a transgenic zebrafish Tg (zlyz-EGFP) model and in vivo proangiogenic activity in a transgenic zebrafish Tg (vegfr2:GFP) model. In the anti-inflammatory activity assay, zebrafish were treated with CuSO_4_, which induced a strong acute inflammatory response, including breaking down the neuromast and mechanosensory cells of the lateral line system in the zebrafish, as well as causing the infiltration and migration of macrophages in the zebrafish. Compared with the model control group, the number of migrating macrophages with fluorescence of zebrafish treated with compounds **11** and **12** was reduced significantly (*p* < 0.01) at concentrations of 20, 40, and 80 μM (Figure 7). In addition, compound **9** also significantly reduced (*p* < 0.01) the migration of zebrafish macrophages at concentrations of 10 and 20 μM. The results indicated compounds **9**, **11**, and **12** had potent anti-inflammatory activity. In addition, blastocolysis or death were observed after exposure to compounds **1**–**5** for 24 h, which showed potential cytotoxicity of these compounds, as well as in other subsequent activity experiments.

**Figure 8:**
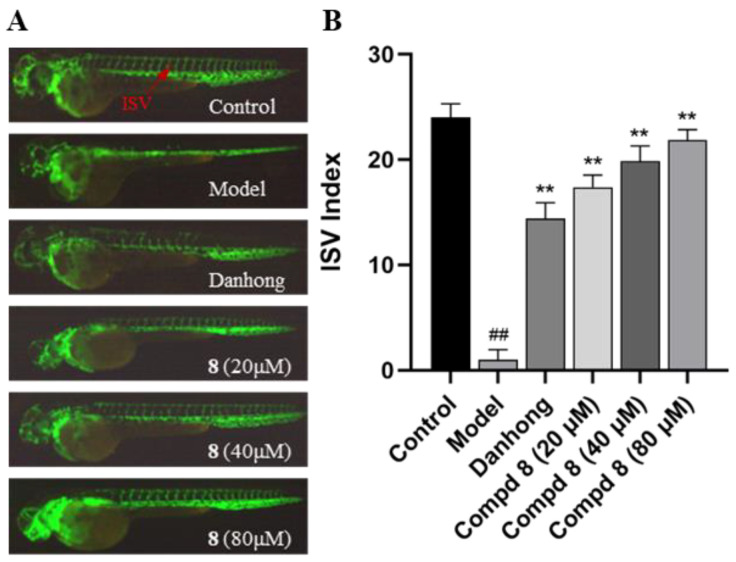
In vivo proangiogenic activities of isolated compounds in fluorescent transgenic zebrafish Tg(vegfr2:GFP) embryos. (**A**) Lateral view of the zebrafish larval trunk in all groups showing ISV (intersegmental blood vessel, red arrows) growth under a fluorescence microscope 24 h after treatment. (**B**) Statistic analysis of the number of ISVs in all groups. Data were derived from 11 independent experiments and represented as mean ± SD; *^##^ p* < 0.01 compared to the control group, ** *p* < 0.01 compared to the model group (PTK787).

In the proangiogenic activity assay, the intersegmental blood vessels (ISVs) of zebrafish in the model group were inhibited by PTK787. Compared to the model group, compound **8** remarkably increased the number of ISVs in model zebrafish in a dose-dependent manner at concentrations of 20, 40, and 80 μM (Figure 8), indicating that **8** exhibited significant proangiogenic activity.

## 3. Materials and Methods

### 3.1. General Experimental Procedures

Optical rotations were obtained on a JASCO P-1020 digital polarimeter. UV spectra were recorded on a Primaide 1430 DAD detector of Hitachi. Electron circular dichroism (ECD) spectra were measured on a JASCO J-815 spectrometer. Measurement of HRESIMS used a Q-TOF Ultima Global GAA076 LC mass spectrometer. The ^1^H, ^13^C, DEPT, and 2D NMR spectral data were recorded on a Bruker Avance 600 spectrometer (Bruker, Karlsruhe, Germany). Vacuum–liquid chromatography (VLC) used silica gel H (Qingdao Marine Chemical Factory, Qingdao, China). Sephadex LH-20 (Pharmacia Biotec AB, Uppsala, Sweden) and reversed-phase C18 silica gel (Merck, Darmstadt, Germany) were used for column chromatography (CC). Thin-layer chromatography (TLC) was performed on plates pre-coated with silica gel GF 254 (10–40 µm) and silica gel (200–300 mesh) (Qingdao Ocean Chemical Co., Ltd., Qingdao, China). MPLC was performed on a SepaBean machineT (Santai Technologies Co., Ltd., Changzhou, China). The semi-preparative HPLC method used was an ODS column (YMC-pack ODS-A, 10 × 250 mm, 5 µm, 4.0 mL/min). All solvents used were of analytical grade (Sinopharm Chemical Reagent Co., Ltd., Shanghai, China).

### 3.2. Fungal Material

The fungal strain was collected from a seawater sample from the Yap Trench in the Pacific Ocean (depth 6215 m, collected in 2017). The strain was identified as *A. dichromosporum* based on ITS sequencing (GenBank No. KF022040) with 99% similarity. The sequence data of YP-213 have been deposited in GenBank with the accession No. OR857042. The strain is preserved at the Shandong Provincial Key Laboratory of Applied Mycology, School of Life Sciences, Qingdao Agricultural University, Qingdao, China.

### 3.3. Fermentation, Extraction, and Isolation

The fungus *A. dichromosporum* YP-213 was transferred aseptically and grown at 28 °C under static conditions. It was cultured in fermentation bags, each containing 80 g rice, 3.96 g sea salt, and 120 mL distilled water.

After the completion of the fermentation period, the whole fermentation broth (60 L) was filtered, and the broth media was extracted with ethyl acetate (EtOAc). The extracts (119 g) were eluted with a stepwise gradient of petroleum ether (PE):EtOAc (1:0, 80:1, 60:1, 40:1, 20:1, 10:1, 5:1, 2:1, and 1:1, *v*/*v*, 1.5 L each) and dichloromethane (CH_2_Cl_2_):methyl alcohol (MeOH) (50:1, 20:1, 10:1, 5:1, 2:1, 1:1, and 0:1, *v*/*v*, 1.5 L each) and fractionated into six fractions (Fr.1–Fr.6) over a silica gel (200–300 mesh) vacuum–liquid chromatography (VLC) column. Fr.2 was further fractioned with MPLC (C-18 ODS) using a step gradient elution of MeOH-H_2_O (5:95 to 100:0) to yield 12 subfractions (Fr.2-1 to Fr.2-12). Fr.2-7 was further fractioned with a Sephadex LH-20 column with MeOH to provide five subfractions (Fr.2-7-1 to Fr.2-7-5). Fr.2-7-2 was separated using semi-preparative HPLC eluted with MeOH-H_2_O (75:25) to obtain compounds **3** (28.8 mg, t_R_ = 30.9 min), **4** (18.2 mg, t_R_ = 36.2 min), and **5** (2.8 mg, t_R_ = 38.7 min). Fr.3 was further fractioned with MPLC (C-18 ODS) using a step gradient elution of MeOH-H_2_O (5:95 to 100:0) to yield 13 subfractions (Fr.3-1 to Fr.3-13). Fr.3-7 was separated using semi-preparative HPLC eluted with MeOH-H_2_O (65:35) to obtain compound **12** (3.8 mg, t_R_ = 5.2 min). Fr.3-8 was separated using semi-preparative HPLC eluted with MeOH-H_2_O (65:35) to obtain compound **7** (5.3 mg, t_R_ = 6.9 min). Fr.3-11 was further fractioned using a Sephadex LH-20 column with MeOH to provide five subfractions (Fr.3-11-1 to Fr.3-11-5). Fr.3-11-3 was separated using semi-preparative HPLC eluted with MeOH-H_2_O (75:25) to obtain compounds **8** (1.4 mg, t_R_ = 9.1 min), **9** (10.6 mg, t_R_ = 10.1 min), **10** (12.4 mg, t_R_ = 12.7 min), and **11** (43.6 mg, t_R_ = 56.8 min). Fr.4 was further fractioned with MPLC (C-18 ODS) using a step gradient elution of MeOH-H_2_O (5:95 to 100:0) to yield 9 subfractions (Fr.4-1 to Fr.4-9). Fr.4-5 and Fr.4-7 were separated using semi-preparative HPLC eluted with MeOH-H_2_O (75:25) to obtain compounds **1** (4.3 mg, t_R_ = 14.0 min) and **2** (3.5 mg, t_R_ = 16.3 min). Fr.5 was further fractioned with MPLC (C-18 ODS) using a step gradient elution of MeOH-H_2_O (5:95 to 100:0) to yield 6 subfractions (Fr.5-1 to Fr.5-6). Fr.5-1 was separated using semi-preparative HPLC eluted with MeOH-H_2_O (75:25) to obtain compound **6** (1.2 mg, t_R_ = 12.0 min).

Acremocholrin A (**1**): pale yellow amorphous powder; [α${]}_{D}^{23}$ +356 (*c* 0.15, MeOH); UV (MeOH) *λ*_max_ 204 (4.87), 227 (5.12), 260 (4.15), 294 (4.62), 323 (4.14), 346 (4.13), and 371 (3.62) nm; ECD (1.2 × 10^−3^ M, MeOH) *λ*_max_ (Δε) 237 (−1.45), 277 (+0.13), 332 (−0.15), and 374 (+0.07); ^1^H and ^13^C NMR data, see Table 1; HRESIMS *m*/*z* 441.1442 [M + Na]^+^ (calculated for C_23_H_27_O_5_ClNa, 441.1439).

Acremocholrin B (**2**): pale yellow amorphous powder; [α${]}_{D}^{23}$ +306 (*c* 0.15, MeOH); UV (MeOH) *λ*_max_ 204 (5.48), 227 (5.73), 260 (4.76), 293 (5.23), 321 (4.80), 346 (4.75), and 373 (4.23) nm; ECD (1.2 × 10^−3^ M, MeOH) *λ*_max_ (Δε) 231 (−0.38), 246 (+0.25), 259 (−0.08), 280 (+0.06), 327 (−0.24), and 374 (+0.07); ^1^H and ^13^C NMR data, see Table 1; HRESIMS *m*/*z* 419.1628 [M + H]^+^ (calculated for C_23_H_28_O_5_Cl, 419.1620).

Acremopyridone A (**7**): white amorphous powder; [α${]}_{D}^{23}$ +115 (*c* 0.06, MeOH); UV (MeOH) *λ*_max_ 222 (5.63), 248 (5.90), 302 (5.20), 326 (5.26), and 371 (4.19); ECD (2.2 × 10^−3^ M, MeOH) *λ*_max_ (Δε) 220 (+1.11), 242 (+4.92), 313 (−0.32), and 374 (+0.09); ^1^H and ^13^C NMR data, see Table 2; HRESIMS *m*/*z* 470.1931 [M + Na]^+^ (calculated for C_27_H_29_O_5_NNa, 470.1938).

Acremoketene A (**12**): pale yellow amorphous powder; [α${]}_{D}^{23}$ −301 (*c* 0.07, MeOH); UV (MeOH) λ_max_ 204 (5.51), 224 (5.57), 249 (5.18), 292 (5.86), 306 (5.83), 318 (5.83), and 371 (4.25); ECD (3.1 × 10^−3^ M, MeOH) *λ*_max_ (Δε) 216 (+4.66), 249 (−0.60), 274 (+4.23), 284 (+3.25), 293 (+3.51), 322 (+0.14), 337 (+0.53), and 355 (−0.04); ^1^H and ^13^C NMR, see Table 3; HRESIMS *m*/*z* 321.1125 [M + H]^+^ (calculated for C_20_H_17_O_4_, 321.1121).

### 3.4. ECD Calculation, Computational NMR Chemical Shift, and DP4+ Analyses

Conformational searches were carried out via molecular mechanics with the MM+ method in HyperChem 8.0 software applying a 21 kJ/mol energy window, and the geometries were optimized at the gas-phase B3LYP/6-31G(d) level in Gaussian 09 software (Version D.01; Gaussian, Inc.: Wallingford, CT, USA) [11] to afford the energy-minimized conformers. Frequency calculations were carried out at the same level of theory to confirm the absence of imaginary frequencies and to obtain thermal corrections to the Gibbs free energies. Then, the optimized conformers were subjected to the calculations of ECD spectra using the TD-DFT at BH&HLYP/TZVP level, and solvent effects of the MeOH solution were evaluated at the same DFT level using the SCRF/PCM method. ECD spectra were generated as the sum of Gaussians using dipole-velocity computed rotational strengths. The calculated ECDs were averaged based on Boltzmann distribution theory.

For NMR calculation, all optimized conformers were subjected to the DFT method at the mPW1PW91/6-31+G(d) PCM level in DMSO to acquire calculated shielding tensors. Then, the calculated shielding tensors were averaged based on Boltzmann distribution theory. Finally, the DP4+ analysis of the calculated shielding tensors and experimental chemical shifts was applied, using the Excel template provided by the original authors [10].

### 3.5. Bioassay

#### 3.5.1. Zebrafish Embryo Acquisition

Healthy transgenic zebrafish Tg (zlyz-EGFP) and Tg (vegfr2:GFP) at 24 h post-fertilization (hpf) were used as the animal model. Adult zebrafish were maintained under a 14/10 h light/dark cycle at a temperature of 28 °C to ensure normal spawning. Afterwards, the eggs were washed and moved to tanks filled with embryo medium. Finally, these embryos were cultured at 28 °C for subsequent experiments.

#### 3.5.2. Anti-Inflammatory Assay

The zebrafish inflammation model was induced with CuSO_4_ to evaluate the effects of compounds on anti-inflammation. In total, 72 hpf zebrafish Tg (zlyz-EGFP) larvae were distributed into 24-well plates (ten embryos per well) in a 2 mL final volume of culture water and treated with different concentrations of each test compound (20 µM, 40 µM, 80 µM) for 2 h as test group. Then, CuSO_4_ was added and incubated for 1 h. The control group was fresh culture water, the model group was 20 µM CuSO_4_, and the positive drug group was 20 µM CuSO_4_ and 10 µM ibuprofen. After 1 h incubation in a light-operated incubator at 28.0 ± 0.5 °C, the number of macrophages was imaged with a fluorescent microscope (Olympus, SZX2-ILLTQ, Tokyo, Japan). All treatments were performed in triplicate.

#### 3.5.3. Pro-Angiogenic Assay

Tg (vegfr2:GFP) zebrafish embryos 24 h post-fertilization were treated with 1 mg/mL pronase E solution to remove the egg membrane. Then, they were randomly divided into 4 groups, namely the normal control group, the model group (vatalanib, PTK787), the positive control group (Danhong injection), and the experimental group. Each group had 10 zebrafish embryos, and each group had two parallel repeats.

As shown in Figure 8A, the model group was built successfully based on significant inhibition of the growth of intersegmental blood vessels (ISVs) by treating zebrafish embryos with vatalanib (PTK787). Then, the positive control group and experimental group were also treated with vatalanib PTK787 (0.2 μg/mL) to afford model zebrafish whose intersegmental blood vessels (ISVs) were significantly inhibited. Simultaneously, the positive control group was given a Danhong injection (10 μL/mL) and the experimental group was given various concentrations of tested compounds (20 μM, 40 μM, 80 μM).

After incubation at 28 °C for 24 h, the intersegmental blood vessels (ISVs) of zebrafish were observed and visualized under a fluorescence microscope (SZX16 stereo microscope and DP2-BSW image acquisition system, Olympus, Japan), and the ISV index was calculated as follows: ISV index = number of intact vessels × 1 + number of defective vessels × 0.5 [12].

## 4. Conclusions

In summary, twelve compounds, including four new compounds (**1**, **2**, **7**, and **12**), were obtained from the hadal trench-derived fungus *Acremonium dichromosporum* YP-213. Among them, acremopyridone A (**7**) featured an unusual *seco*-pyridone alkaloid. The stereoconfigurations of isolated compounds were determined with NOE analysis, octant rule and quantum chemical calculations of ECD, and NMR (with DP4+ probability analysis). Compounds **9**, **11**, and **12** showed potent in vivo anti-inflammatory activity in transgenic zebrafish, while compound **8** exhibited significant proangiogenic activity in transgenic zebrafish. The above compounds possess the potential to be developed as lead compounds in anti-inflammatory or cardiovascular drug discovery.

## Figures and Tables

**Table 1:** ^1^H and ^13^C NMR data for compounds **1** and **2** (measured in DMSO-*d*_6_).

No.	1		2	
	*δ*_C_, Type ^a^	*δ*_H_ (*J* in Hz) ^b^	*δ*_C_, Type ^a^	*δ*_H_ (*J* in Hz) ^b^
1	112.8, C		112.6, C	
2	161.6, C		161.6, C	
3	112.4, C		112.4, C	
4	159.4, C		159.6, C	
5	114.0, C		113.7, C	
6	139.2, C		139.2, C	
7	14.4, CH_3_	2.58, s	14.4, CH_3_	2.58, s
8	195.1, CH	10.12, s	195.0, CH	10.12, s
1′	30.7, CH_2_	3.02, dd (14.2, 2.4) 2.71, dd (14.2, 8.9)	30.7, CH_2_	3.02, dd (14.2, 2.6) 2.73, dd (14.2, 8.7)
2′	69.9, CH	4.61, d (7.1)	69.9, CH	4.62, d (8.6)
3′	113.0, C		113.0, C	
4′	128.2, CH	6.04, d (16.6)	128.3, CH	6.04, d (16.6)
5′	136.9, CH	5.92, d (16.6)	136.9, CH	5.90, d (16.6)
6′	47.7, C		47.7, C	
7′	40.7, CH	2.75 dt (7.4, 2.5)	40.8, CH	2.77, dt (7.5, 2.8)
8′	152.7, CH	6.70, dd (10.1, 1.8)	152.9, CH	6.72, dd (10.1, 1.9)
9′	127.3, CH	5.94, dd (9.9, 3.1)	127.3, CH	5.94, dd (10.1, 3.1)
10′	200.0, C		200.0, C	
11′	50.5, CH	2.62, q (6.7)	50.3, CH	2.61, q (6.7)
12′	148.2, CH_2_	5.25, s 5.12, s	148.3, CH_2_	5.24, s 5.12, s
13′	9.4, CH_3_	0.76, s	9.4, CH_3_	0.75, s
14′	14.7, CH_3_	0.92, d (7.5)	14.8, CH_3_	0.96, d (7.5)
15′	8.9, CH_3_	0.87, d (6.8)	8.9, CH_3_	0.82, d (6.8)
2-OH		12.86, s		12.90, s

^a^ Measured at 150 MHz; ^b^ measured at 600 MHz.

**Table 2:** ^1^H and ^13^C NMR data for compound **7** (measured in DMSO-*d*_6_).

No.	*δ*_C_, Type ^a^	*δ*_H_ (*J* in Hz) ^b^	No.	*δ*_C_, Type ^a^	*δ*_H_ (*J* in Hz) ^b^
1-NH		11.89, brs	15	55.5, CH	2.11, td (11.8, 3.3)
2	159.1, C		16	212.2, C	
3	- ^c^		17	151.7, CH	7.91, s
4	164.1, C		18	27.2, CH_3_	2.22, s
5	111.1, C		19	22.5, CH_3_	0.83, d (6.5)
6	138.1, CH	7.59, s	20	126.8, CH	5.66, dd (14.9, 10.7)
7	174.4, C		21	128.4, CH	5.37, dd (14.9, 6.6)
8	129.0, C		22	17.9, CH_3_	1.66, d (6.6, 1.3)
9	41.1, CH	3.29, td (10.7, 4.6)	1′	122.8, C	
10	40.1, CH	1.98, t (11.8)	2′/6′	130.5, CH	7.29, d (8.5)
11	34.2, CH_2_	1.53, d (12.6) 0.63, q (12.6)	3′/5′	115.2, CH	6.82, d (8.5)
12	31.8, CH	1.24, m	4′	157.1, C	
13	33.8, CH_2_	1.63, m 0.80, m	4′-OH		9.60, s
14	29.3, CH_2_	1.71, dd (12.7, 3.0) 1.32, dd (12.7, 3.0)			

^a^ Measured at 150 MHz; ^b^ measured at 600 MHz; ^c^ not detected.

**Table 3:** ^1^H and ^13^C NMR data for compound **12** (measured in DMSO-*d*_6_).

No.	*δ*_C_, Type ^a^	*δ*_H_ (*J* in Hz) ^b^	No.	*δ*_C_, Type ^a^	*δ*_H_ (*J* in Hz) ^b^
1	200.0, C		9	125.4, C	
2	144.7, C		10/14	131.1, CH	7.64 d (8.7)
3	154.5, C		11/13	116.2, CH	6.80 d (8.7)
4	24.4, CH_2_	3.07 (m, 2H)	12	160.5, C	
5	31.6, CH_2_	2.57 (m, 2H)	15	132.3, C	
6	163.5, C		16/20	127.4, CH	7.79 dd (7.8, 1.9)
7	111.1, CH	6.63 (d, 15.9)	17/19	129.0, CH	7.52 m
8	147.9, CH	7.75 (d, 15.9)	18	130.9, CH	7.51 m

^a^ Measured at 150 MHz; ^b^ measured at 600 MHz.

## Data Availability

The original data presented in the study are included in the article/Supplementary Material; further inquiries can be directed to the corresponding author.

## Supplementary Materials

The following supporting information can be downloaded at: https://www.mdpi.com/article/10.3390/md22010025/s1, Tables S1–S5: The Cartesian coordinates of the optimized conformers for compounds **1**, **2**, and **7**. Figure S1: HPLC chromatogram of compounds **1** and **2**. Figures S2–S28: The 1D and 2D NMR spectra, HRESIMS. Figure S29–S33: optimized geometries of predominant conformers for compounds **1**, **2**, **7**, and **12**. Figure S34: The DP4+ probability analysis of compound **7**.
