# Supplementary material for: Anti-Inflammatory and Proangiogenic Metabolites from the Hadal Trench-Derived Fungus Acremonium dichromosporum YP-213
Source: Mar Drugs. 2023 Dec 29;22(1):25. doi: 10.3390/md22010025 (PMC10817564; doi:10.3390/md22010025)
Supplement: Supplementary file 1 [file marinedrugs-22-00025-s001.zip › marinedrugs-2779276-supplementary.pdf]

Supporting Information (SI)

Anti-inflammatory and Proangiogenic Metabolites from the Hadal Trench-derived Fungus *Acremonium dichromosporum* YP-213

Yan Zhang[1,+], Jia-Bao Zhou[1,+], Shu-Ting Yang[1], Xin Liu[1], Wei Cao[1], Pei-Hai Li[2], Hao Chen[3], Ya-Qin Fan[1,3,*]

[1] Shandong Provincial Key Laboratory of Applied Mycology, School of Life Sciences, Qingdao Agricultural University, Qingdao 266109, China

[2] Engineering Research Center of Zebrafish Models for Human Diseases and Drug Screening of Shandong Province, Shandong Provincial Engineering Laboratory for Biological Testing Technology, Key Laboratory for Biosensor of Shandong Province, Biology Institute, Qilu University of Technology (Shandong Academy of Sciences), Jinan 250103, China

[3] MNR Key Laboratory of Marine Eco-Environmental Science and Technology, First Institute of Oceanography, Ministry of Natural Resources, Qingdao 266061, China

***** Correspondence: fanyaqin@qau.edu.cn; Tel.: +86-532-58957640

[+] Y.Z. and J.-B.Z. contributed equally to the work.

Contents

Table S1. The Cartesian coordinates of the optimized conformers (above 2% population, from 128 conformers in total) for (2'*R*,6'*S*,7'*R*,11'*R*)-**1**.

Table S2. The Cartesian coordinates of the optimized conformers (above 2% population, from 129 conformers in total) for (2'*S*,6'*S*,7'*R*,11'*R*)-**2**.

Table S3. The Cartesian coordinates of the optimized conformers (above 2% population, from 29 conformers in total) for (9*S*,10*S*,12*R*,15*R*)-**7**.

Table S4. The Cartesian coordinates of the optimized conformers (above 2% population, from 34 conformers in total) for (9*R*,10*R*,12*S*,15*S*)-**7**.

Table S5. The Cartesian coordinates of the optimized conformers (above 2% population, from 25 conformers in total) for (9*S**,10*R**,12*S**,15*S**)-**7**.

Figure S1. HPLC chromatogram of acremocholrins A (**1**) and B (**2**) at 225nm.

Figure S2. HRESIMS spectrum of acremocholrin A (**1**).

Figure S3. ^{1}H NMR (600 MHz, DMSO-d_6) spectrum of acremocholrin A (**1**).

Figure S4. DEPT-Q (150 MHz, DMSO-d_6) spectra of acremocholrin A (**1**).

Figure S5. HSQC spectrum of acremocholrin A (**1**).

Figure S6. ^{1}H-^{1}H COSY spectrum of acremocholrin A (**1**).

Figure S7. HMBC spectrum of acremocholrin A (**1**).

Figure S8. NOE difference spectrum of acremocholrin A (**1**).

Figure S9. HRESIMS spectrum of acremocholrin B (**2**).

Figure S10. ^{1}H NMR (600 MHz, DMSO-d_6) spectrum of acremocholrin B (**2**).

Figure S11. DEPT-Q (150 MHz, DMSO-d_6) spectrum of acremocholrin B (**2**).

Figure S12. HSQC spectrum of acremocholrin B (**2**).

Figure S13. ^{1}H-^{1}H COSY spectrum of acremocholrin B (**2**).

Figure S14. HMBC spectrum of acremocholrin B (**2**).

Figure S15. NOE difference spectrum of acremocholrin B (**2**).

Figure S16. HRESIMS spectrum of Acremopyridone A (**7**).

Figure S17. ^{1}H NMR (600 MHz, DMSO-d_6) spectrum of Acremopyridone A (**7**).

Figure S18. DEPT-Q (150 MHz, DMSO-d_6) spectrum of Acremopyridone A (**7**).

Figure S19. HSQC spectrum of Acremopyridone A (**7**).

Figure S20. ^{1}H-^{1}H COSY spectrum of Acremopyridone A (**7**).

Figure S21. HMBC spectrum of Acremopyridone A (**7**).

Figure S22. NOESY spectrum of Acremopyridone A (**7**).

Figure S23. HRESIMS spectrum of acremoketene A (**12**).

Figure S24. ^{1}H NMR (600 MHz, DMSO-d_6) spectrum of acremoketene A (**12**).

Figure S25. DEPT-Q (150 MHz, DMSO-d_6) spectrum of acremoketene A (**12**).

Figure S26. HSQC spectrum of acremoketene A (**12**).

Figure S27. ^{1}H-^{1}H COSY spectrum of acremoketene A (**12**).

Figure S28. HMBC spectrum of acremoketene A (**12**).

Figure S29. Optimized geometries of predominant conformers (weighting factors) for (2'*R*,6'*S*,7'*R*,11'*R*)-**1** at the B3LYP/6-31g(d) level.

Figure S30. Optimized geometries of predominant conformers (weighting factors) for (2'*S*,6'*S*,7'*R*,11'*R*)-**2** at the B3LYP/6-31g(d) level.

Figure S31. Optimized geometries of predominant conformers (weighting factors) for (9*S*,10*S*,12*R*,15*R*)-**7** at the B3LYP/6-31g(d) level.

Figure S32. Optimized geometries of predominant conformers (weighting factors) for (9*R*,10*R*,12*S*,15*S*)-**7** at the B3LYP/6-31g(d) level.

Figure S33. Optimized geometries of predominant conformers (weighting factors) for (9*S**,10*R**,12*S**,15*S**)-**7** at the B3LYP/6-31g(d) level.

Figure S34. DP4+ probability analysis of compound **7** (mPW1PW91/6-31+G(d, p) level).

Table S1. The Cartesian coordinates of the optimized conformers (above 2% population, from 128 conformers in total) for (2'*R*,6'*S*,7'*R*,11'*R*)-**1**.

Conf. A	X axis(Å)	Y axis(Å)	Z axis(Å)	Conf. B	X axis(Å)	Y axis(Å)	Z axis(Å)
C	-4.49567	1.165572	-0.22302	C	3.187929	1.797428	-0.01265
C	-5.05135	0.013105	-0.852	C	4.429641	1.144006	0.249385
C	-4.4582	-1.20909	-0.57796	C	4.513555	-0.21405	-0.01191
C	-3.34145	-1.33115	0.290215	C	3.407308	-0.95423	-0.51675
C	-2.79588	-0.21287	0.920708	C	2.186413	-0.3201	-0.8018
C	-3.37435	1.033955	0.645815	C	2.089483	1.055602	-0.53576
O	-2.8373	2.112049	1.244765	O	0.925743	1.669217	-0.8101
O	-2.76654	-2.52604	0.545898	O	3.60277	-2.26406	-0.71141
Cl	-5.05016	-2.71794	-1.29827	Cl	5.991508	-1.12271	0.283674
C	-6.23964	0.136618	-1.78016	C	5.602229	1.924714	0.800962
C	-5.04535	2.487105	-0.45491	C	3.017272	3.213229	0.228265
H	-5.90599	2.564682	-1.13798	H	3.879861	3.763604	0.635469
O	-4.61342	3.528145	0.062003	O	1.972323	3.849178	0.008304
C	-1.61493	-0.32893	1.853197	C	1.024777	-1.02885	-1.47332
C	-0.26549	-0.14851	1.126625	C	0.288721	-2.13481	-0.68202
C	0.956066	-0.17321	2.045522	C	-0.09341	-1.75869	0.740282
C	2.286544	-0.16305	1.417041	C	-1.35055	-1.04013	0.985782
C	0.866193	-0.20389	3.386925	C	0.707136	-2.08236	1.772813
C	2.544945	-0.13203	0.099818	C	-2.25594	-0.64987	0.073414
C	3.910083	-0.02645	-0.56704	C	-3.56096	0.082347	0.323235
C	4.280513	1.505962	-0.61628	C	-3.5206	1.417668	-0.50866
C	5.702828	1.733649	-1.06591	C	-4.88681	2.043627	-0.65445
C	6.677668	0.818876	-0.9599	C	-6.04403	1.379964	-0.51477
C	6.424746	-0.5154	-0.38113	C	-6.072	-0.0645	-0.21546
C	5.037971	-0.75815	0.23567	C	-4.72539	-0.79992	-0.26438
C	3.797151	-0.62008	-1.99015	C	-3.79685	0.366068	1.818331
C	4.816361	-2.25773	0.468921	C	-4.85063	-2.20635	0.329532
C	3.298619	2.377481	-1.42027	C	-2.50044	2.449896	0.003724
O	7.299757	-1.37044	-0.36021	O	-7.12121	-0.64885	0.015666
O	-0.27171	1.030635	0.313335	O	1.143416	-3.29681	-0.72767
H	-3.37308	2.904803	0.953499	H	1.046768	2.635916	-0.57889
H	-3.24495	-3.22187	0.060071	H	2.738566	-2.73329	-0.82372
H	-7.10118	0.577255	-1.2665	H	5.9096	2.719754	0.111936
H	-6.00684	0.77385	-2.6406	H	5.35125	2.397112	1.757295
H	-6.54685	-0.83551	-2.16414	H	6.46254	1.277765	0.965479
H	-1.62675	-1.30454	2.346722	H	1.363462	-1.49865	-2.40729
H	-1.714	0.437807	2.628757	H	0.291022	-0.26975	-1.74935
H	-0.15958	-0.97114	0.408821	H	-0.62106	-2.38362	-1.24347
H	3.11505	-0.18112	2.12562	H	-1.5326	-0.81916	2.035206

H	1.760472	-0.21867	4.005357	H	0.440233	-1.82081	2.792798
H	-0.07801	-0.22025	3.919385	H	1.655168	-2.59301	1.637235
H	1.705587	-0.0935	-0.58962	H	-2.07546	-0.86301	-0.98133
H	4.220176	1.854028	0.428348	H	-3.19813	1.134413	-1.52616
H	5.930115	2.712725	-1.48917	H	-4.90517	3.10794	-0.89011
H	7.692522	1.00841	-1.30002	H	-7.00916	1.871534	-0.60573
H	5.09149	-0.26281	1.220307	H	-4.5046	-0.90562	-1.3414
H	4.730627	-0.51645	-2.55177	H	-4.72015	0.932815	1.96958
H	3.546321	-1.68418	-1.94458	H	-3.87899	-0.56319	2.389311
H	3.004349	-0.1257	-2.56071	H	-2.9763	0.947152	2.248831
H	4.868895	-2.81899	-0.46903	H	-5.63433	-2.76045	-0.19256
H	3.844891	-2.44501	0.932637	H	-5.13491	-2.17475	1.385755
H	5.602103	-2.65295	1.117267	H	-3.90887	-2.75544	0.241013
H	3.564411	3.43583	-1.31331	H	-2.43067	3.288815	-0.69835
H	2.273195	2.251116	-1.06007	H	-1.50477	2.007482	0.096654
H	3.321426	2.141011	-2.48946	H	-2.79209	2.860323	0.976189
H	-0.65229	1.750576	0.843924	H	0.867714	-3.91149	-0.02798
Free Energy = -1728.579667 Hartree				Free Energy = -1728.576479 Hartree			

Table S2. The Cartesian coordinates of the optimized conformers (above 2% population, from 129 conformers in total) for (2'*S*,6'*S*,7'*R*,11'*R*)-**2**.

Conf. A	X axis(Å)	Y axis(Å)	Z axis(Å)	Conf. B	X axis(Å)	Y axis(Å)	Z axis(Å)
C	4.955389	0.402523	0.109914	C	4.716257	-0.54234	0.565521
C	4.94341	-0.99107	-0.17081	C	4.956366	0.841326	0.308753
C	3.702387	-1.62492	-0.20745	C	3.890521	1.615379	-0.1243
C	2.495801	-0.94339	0.048419	C	2.592228	1.065878	-0.31281
C	2.499121	0.421947	0.358129	C	2.331188	-0.2813	-0.01705
C	3.73767	1.086395	0.355146	C	3.408154	-1.08208	0.398406
O	3.806408	2.412214	0.627237	O	3.16187	-2.37667	0.651039
O	1.295756	-1.57708	0.023314	O	1.636934	1.896072	-0.75776
Cl	3.548423	-3.35888	-0.56967	Cl	4.077908	3.32514	-0.49591
C	6.222983	-1.74552	-0.434	C	6.340532	1.42318	0.49593
C	6.199896	1.197435	0.127781	C	5.776016	-1.42204	1.010419
H	6.040935	2.275406	0.303198	H	6.778398	-0.98259	1.134351
O	7.332429	0.765002	-0.03463	O	5.633845	-2.63014	1.261082
C	1.205035	1.103758	0.753134	C	0.931817	-0.86676	-0.04329
C	0.582074	2.020669	-0.31864	C	0.355129	-1.13908	-1.44626
C	-0.883	2.322144	-0.03634	C	-0.81082	-2.11629	-1.46109
C	-1.81649	1.233544	-0.38879	C	-2.10163	-1.80882	-0.82426
C	-1.26623	3.495784	0.487111	C	-0.62617	-3.31383	-2.04639
C	-2.92232	0.913327	0.299411	C	-2.52286	-0.62526	-0.34742

C	-3.9435	-0.16093	-0.02031	C	-3.86144	-0.31886	0.297719
C	-3.99164	-1.14924	1.20388	C	-3.56916	0.258873	1.732879
C	-5.23006	-2.0129	1.200815	C	-4.76549	0.965486	2.324578
C	-6.3564	-1.72217	0.533519	C	-5.79152	1.447169	1.607849
C	-6.48428	-0.4839	-0.25908	C	-5.82258	1.336592	0.136407
C	-5.3503	0.539813	-0.11572	C	-4.54093	0.829982	-0.53735
C	-3.61818	-0.9077	-1.32684	C	-4.77979	-1.55249	0.373632
C	-5.46844	1.635311	-1.17998	C	-4.78913	0.508771	-2.01416
C	-2.73139	-2.02021	1.359406	C	-3.00861	-0.77596	2.725049
O	-7.46582	-0.27131	-0.95659	O	-6.80596	1.673521	-0.50781
O	1.392854	3.206762	-0.33485	O	-0.01806	0.154849	-1.98102
H	2.963436	2.844513	0.361729	H	4.025814	-2.78362	0.947812
H	1.450407	-2.52167	-0.15958	H	0.919508	1.37282	-1.18822
H	6.764364	-1.30839	-1.27736	H	6.354576	2.487234	0.264575
H	6.899674	-1.66679	0.421098	H	6.685731	1.301222	1.528779
H	6.028687	-2.79798	-0.64026	H	7.071144	0.929895	-0.15528
H	0.472597	0.338333	1.014704	H	0.954449	-1.81409	0.498404
H	1.358129	1.7205	1.648745	H	0.240723	-0.20486	0.492316
H	0.655472	1.517112	-1.2948	H	1.156498	-1.55876	-2.06715
H	-1.54376	0.647189	-1.26611	H	-2.77475	-2.66296	-0.77388
H	-2.31418	3.728735	0.649224	H	-1.40419	-4.07275	-2.04951
H	-0.53898	4.253743	0.759313	H	0.310329	-3.58001	-2.52864
H	-3.1391	1.48089	1.207204	H	-1.86748	0.238598	-0.43902
H	-4.05387	-0.51631	2.106487	H	-2.78744	1.027495	1.600161
H	-5.17785	-2.9303	1.787791	H	-4.77074	1.077963	3.409109
H	-7.21902	-2.38342	0.536904	H	-6.6472	1.929963	2.07271
H	-5.51789	1.005848	0.871317	H	-3.84307	1.684372	-0.48968
H	-3.62678	-0.22653	-2.18245	H	-5.02922	-1.92154	-0.62538
H	-4.34872	-1.6984	-1.5219	H	-5.71767	-1.31492	0.884344
H	-2.62887	-1.37249	-1.28615	H	-4.30387	-2.37318	0.918386
H	-6.4474	2.115442	-1.10967	H	-3.86205	0.205454	-2.50897
H	-5.38897	1.225217	-2.19128	H	-5.19174	1.388031	-2.52279
H	-4.69152	2.394472	-1.05248	H	-5.52448	-0.29257	-2.13505
H	-2.74892	-2.54216	2.322951	H	-2.66867	-0.27845	3.640435
H	-1.82113	-1.41441	1.319384	H	-2.15719	-1.31159	2.294713
H	-2.66794	-2.78086	0.574183	H	-3.76582	-1.51299	3.011987
H	1.135725	3.7643	-1.0859	H	-0.22362	0.068202	-2.92502
Free Energy = -1728.578787 Hartree				Free Energy = -1728.576636 Hartree			

Table S3. The Cartesian coordinates of the optimized conformers (above 2% population, from 29 conformers in total) for (9*S*,10*S*,12*R*,15*R*)-**7**.

Conf. A	X axis(Å)	Y axis(Å)	Z axis(Å)	Conf. B	X axis(Å)	Y axis(Å)	Z axis(Å)
C	-2.95542	-1.69782	4.051597	C	-4.70659	1.87658	-2.9901
C	-2.92644	-3.63058	-2.95875	C	-3.88475	0.975657	4.105885
C	-2.77086	-2.2204	-2.46843	C	-3.17637	0.161056	3.060506
C	-3.35349	-0.77234	2.89632	C	-4.17537	0.570809	-2.38761
C	-4.85754	-0.8547	2.589643	C	-5.30345	-0.44073	-2.13901
C	-5.24939	0.060099	1.422414	C	-4.77412	-1.7402	-1.52207
C	-4.43223	-0.2536	0.14675	C	-3.97819	-1.48448	-0.21231
C	-2.91097	-0.15831	0.442741	C	-2.84328	-0.46067	-0.44977
C	-2.54585	-1.07916	1.62296	C	-3.4047	0.827352	-1.08171
C	0.431697	-0.82925	-0.56014	C	0.376491	-0.67641	0.496191
C	-0.61085	0.017091	-0.63338	C	-0.59264	0.255553	0.566964
C	-2.04747	-0.43954	-0.841	C	-2.01456	-0.21961	0.865034
O	1.729376	-0.44573	-0.38628	O	1.690198	-0.42108	0.241331
C	2.04009	0.867818	-0.26811	C	2.105738	0.857414	0.067581
C	1.061008	1.849635	-0.34174	C	1.210805	1.917373	0.117817
C	-0.35025	1.476924	-0.56638	C	-0.23508	1.675137	0.337758
C	3.436932	1.135077	-0.05227	C	3.520739	1.000581	-0.15095
C	3.762055	2.457764	0.08619	C	3.957901	2.288735	-0.30476
N	2.827641	3.440843	0.031197	N	3.111829	3.348597	-0.24883
C	1.438601	3.261176	-0.18385	C	1.70972	3.289691	-0.04979
O	0.718597	4.2444	-0.21217	O	1.07928	4.333184	-0.03411
O	-1.25845	2.295067	-0.70039	O	-1.07697	2.5704	0.326669
C	-2.16726	-1.8647	-1.33046	C	-2.72137	0.634974	1.896074
C	4.476561	0.080138	0.049262	C	4.475706	-0.13556	-0.18269
C	4.60388	-0.9395	-0.91194	C	5.679346	-0.06511	0.540766
C	5.612408	-1.89074	-0.82688	C	6.622753	-1.08571	0.489313
C	6.530972	-1.84432	0.228892	C	6.373147	-2.22098	-0.28771
C	6.423235	-0.8403	1.195063	C	5.177483	-2.3166	-1.00823
C	5.401758	0.103963	1.103249	C	4.244808	-1.28439	-0.95578
O	7.501094	-2.80483	0.260913	O	7.326037	-3.19968	-0.30047
C	-4.88163	0.62705	-1.02798	C	-3.46946	-2.84235	0.275348
C	-4.6982	2.130727	-0.93181	C	-4.40577	-3.65091	1.15701
O	-5.37334	0.116377	-2.02163	O	-2.37266	-3.27394	-0.0415
H	-2.69135	0.873689	0.741235	H	-2.14975	-0.92629	-1.16222
H	-4.66078	-1.27941	-0.1671	H	-4.67618	-1.08449	0.53739
H	-1.88663	-1.61141	4.28225	H	-3.89174	2.581174	-3.1923
H	-3.51542	-1.45885	4.963758	H	-5.23719	1.693865	-3.93277
H	-3.15847	-2.74759	3.8025	H	-5.40677	2.369019	-2.30301
H	-3.98715	-3.89003	-3.0741	H	-4.88704	0.576734	4.314873
H	-2.46439	-3.76035	-3.94686	H	-3.33797	0.961335	5.05869
H	-2.47099	-4.35328	-2.27257	H	-3.99315	2.019411	3.793545
H	-3.21087	-1.44392	-3.09434	H	-3.0222	-0.89419	3.301236

H	-3.12926	0.263939	3.195999	H	-3.4774	0.120165	-3.11214
H	-5.1172	-1.89563	2.343853	H	-6.04787	0.008776	-1.4643
H	-5.44051	-0.58982	3.481914	H	-5.82796	-0.66699	-3.07718
H	-6.32079	-0.04011	1.205961	H	-5.60534	-2.43086	-1.32901
H	-5.08354	1.104435	1.719593	H	-4.10862	-2.24196	-2.23814
H	-2.7282	-2.1276	1.345017	H	-4.08259	1.315827	-0.36665
H	-1.47238	-0.99467	1.839048	H	-2.59341	1.540207	-1.25467
H	0.373669	-1.90645	-0.64554	H	0.214228	-1.74157	0.619587
H	-2.43967	0.217707	-1.62656	H	-1.88657	-1.21006	1.314339
H	4.787651	2.777261	0.235116	H	5.00193	2.516214	-0.49021
H	3.098587	4.41288	0.126293	H	3.460702	4.29152	-0.37665
H	-1.76266	-2.65116	-0.69091	H	-2.8382	1.688542	1.659183
H	3.9085	-0.98163	-1.74374	H	5.870557	0.798457	1.17245
H	5.710023	-2.67284	-1.57307	H	7.546887	-1.02681	1.055383
H	7.125288	-0.80376	2.026137	H	4.978237	-3.19629	-1.61778
H	5.312175	0.861392	1.877549	H	3.329812	-1.37223	-1.53178
H	8.064691	-2.66173	1.037216	H	7.023641	-3.92905	-0.86379
H	-5.16267	2.535651	-0.02521	H	-5.42413	-3.67103	0.752394
H	-3.63082	2.381974	-0.88715	H	-4.02789	-4.66899	1.27253
H	-5.14659	2.599991	-1.81014	H	-4.46753	-3.17478	2.144864
Free Energy = -1476.753209 Hartree				Free Energy = -1476.752728 Hartree			
**Conf. C**	**X axis(Å)**	**Y axis(Å)**	**Z axis(Å)**	**Conf. D**	**X axis(Å)**	**Y axis(Å)**	**Z axis(Å)**
C	6.861312	-1.54071	-0.91768	C	6.853575	-1.56303	-0.92685
C	2.729006	-3.68891	3.394088	C	2.714198	-3.71246	3.371141
C	2.681618	-2.31631	2.785916	C	2.671125	-2.33609	2.771188
C	5.474896	-1.00775	-1.29714	C	5.471701	-1.01504	-1.30147
C	5.537381	0.424272	-1.83933	C	5.545971	0.422175	-1.82819
C	4.140147	0.92602	-2.20601	C	4.152991	0.939232	-2.18981
C	3.134594	0.868899	-1.01727	C	3.146728	0.87684	-1.00204
C	3.077024	-0.55405	-0.39747	C	3.076615	-0.55289	-0.39935
C	4.508304	-1.06485	-0.10607	C	4.503579	-1.07786	-0.11186
C	-0.17614	-1.01891	0.344971	C	-0.17873	-1.01879	0.329903
C	0.793126	-0.21053	0.805274	C	0.789971	-0.21506	0.799668
C	2.244689	-0.65119	0.9328	C	2.240733	-0.6584	0.928134
O	-1.48293	-0.6583	0.213603	O	-1.48511	-0.65611	0.199308
C	-1.87636	0.5945	0.541187	C	-1.87764	0.594548	0.53474
C	-0.97729	1.525104	1.045973	C	-0.97906	1.520868	1.047933
C	0.441788	1.163864	1.22624	C	0.438653	1.155862	1.231563
C	-3.27722	0.853264	0.342874	C	-3.27804	0.855634	0.335862
C	-3.69507	2.106913	0.699165	C	-3.69532	2.107998	0.69653
N	-2.84101	3.038557	1.19704	N	-2.84116	3.036383	1.200693
C	-1.44656	2.875685	1.378476	C	-1.44754	2.870193	1.38639
O	-0.79004	3.827924	1.768338	O	-0.79113	3.819373	1.783902

O	1.297938	1.931961	1.666655	O	1.293583	1.918605	1.683533
C	2.318	-2.04246	1.530534	C	2.309691	-2.05364	1.517079
C	-4.23468	-0.14857	-0.18906	C	-4.23471	-0.14505	-0.19985
C	-5.46329	-0.36769	0.457452	C	-3.97931	-0.8542	-1.38819
C	-6.40433	-1.25853	-0.04835	C	-4.90364	-1.75347	-1.90397
C	-6.12662	-1.96677	-1.22141	C	-6.11864	-1.96592	-1.24127
C	-4.9057	-1.76988	-1.87634	C	-6.39249	-1.27205	-0.05956
C	-3.97485	-0.8698	-1.36519	C	-5.45382	-0.37722	0.452751
O	-7.07863	-2.83506	-1.67539	O	-6.98895	-2.86166	-1.7949
C	1.817679	1.374278	-1.61893	C	1.834629	1.401697	-1.59734
C	1.568354	2.870618	-1.55634	C	1.59965	2.89957	-1.51792
O	1.046641	0.620458	-2.19006	O	1.055772	0.661595	-2.17555
H	2.606858	-1.21306	-1.14183	H	2.602782	-1.19957	-1.15211
H	3.466481	1.579345	-0.24856	H	3.485219	1.574895	-0.22481
H	6.806921	-2.57462	-0.55602	H	6.790651	-2.60019	-0.57604
H	7.543155	-1.52132	-1.77658	H	7.536045	-1.5402	-1.78517
H	7.311422	-0.93353	-0.12161	H	7.308171	-0.96785	-0.12427
H	3.738609	-3.93067	3.753045	H	2.415586	-4.47849	2.647086
H	2.062432	-3.76169	4.264242	H	3.722789	-3.95909	3.729657
H	2.431687	-4.46006	2.67493	H	2.046543	-3.78872	4.240194
H	2.969269	-1.4905	3.438989	H	2.960345	-1.51503	3.429569
H	5.069509	-1.64966	-2.09604	H	5.062006	-1.64506	-2.10758
H	5.984094	1.08236	-1.07872	H	5.997756	1.068444	-1.06045
H	6.19103	0.473751	-2.72087	H	6.200384	0.475743	-2.70892
H	4.19676	1.953644	-2.5869	H	4.218023	1.970562	-2.55917
H	3.737194	0.312149	-3.02321	H	3.745351	0.337896	-3.01397
H	4.930565	-0.46781	0.718383	H	4.929478	-0.49342	0.719748
H	4.455759	-2.09265	0.268831	H	4.442991	-2.1094	0.251323
H	-0.03979	-2.04094	0.01479	H	-0.04268	-2.03802	-0.0089
H	2.693365	0.053281	1.643036	H	2.688499	0.040312	1.64461
H	-4.72821	2.415546	0.583184	H	-4.72782	2.418341	0.579232
H	-3.17185	3.970003	1.420912	H	-3.17082	3.967668	1.42684
H	2.038821	-2.87078	0.873866	H	2.028765	-2.87712	0.855141
H	-5.67782	0.156235	1.385366	H	-3.04778	-0.69059	-1.92018
H	-7.34894	-1.42745	0.458723	H	-4.70566	-2.29529	-2.82335
H	-4.68557	-2.31494	-2.79267	H	-7.32949	-1.4398	0.468622
H	-3.03958	-0.71944	-1.89431	H	-5.66703	0.134526	1.387669
H	-6.75724	-3.26222	-2.4847	H	-7.78248	-2.91718	-1.23997
H	0.717438	3.131611	-2.18973	H	0.757212	3.177013	-2.15565
H	1.367236	3.169468	-0.52065	H	1.390338	3.185758	-0.48022
H	2.454795	3.429095	-1.88051	H	2.494394	3.454074	-1.82545
Free Energy = -1476.752714 Hartree				Free Energy = -1476.752684 Hartree			
**Conf. E**	**X axis(Å)**	**Y axis(Å)**	**Z axis(Å)**	**Conf. F**	**X axis(Å)**	**Y axis(Å)**	**Z axis(Å)**

C	-3.99949	2.1861	-3.23054	C	2.77312	-1.38727	4.019655
C	-3.8918	0.438914	4.136262	C	2.581104	4.536239	-1.01473
C	-3.16174	-0.24699	3.015855	C	2.460299	3.062166	-1.28038
C	-3.7732	0.805822	-2.60313	C	3.323935	-1.26769	2.594153
C	-5.08784	0.027827	-2.44914	C	4.7831	-0.7861	2.587183
C	-4.85821	-1.34721	-1.81207	C	5.325809	-0.62586	1.162139
C	-4.12818	-1.24055	-0.44518	C	4.434398	0.320651	0.313783
C	-2.80415	-0.45309	-0.58749	C	2.980787	-0.20816	0.283021
C	-3.06171	0.916721	-1.24373	C	2.453295	-0.34255	1.724996
C	0.377369	-0.79453	0.433169	C	-0.41639	0.94725	-0.24179
C	-0.597	0.119632	0.597521	C	0.607599	0.159547	-0.61468
C	-2.02978	-0.38237	0.776441	C	2.050568	0.652415	-0.64629
O	1.699513	-0.50931	0.265895	O	-1.72574	0.560966	-0.21485
C	2.11574	0.780981	0.276698	C	-2.06678	-0.70395	-0.56308
C	1.217004	1.825294	0.443806	C	-1.111	-1.61712	-0.98413
C	-0.2324	1.557115	0.603447	C	0.312554	-1.21948	-1.08354
C	3.531493	0.953006	0.08501	C	-3.47047	-0.99858	-0.4443
C	3.963489	2.251901	0.073637	C	-3.82618	-2.2794	-0.77147
N	3.111093	3.296402	0.229685	N	-2.91391	-3.20106	-1.17213
C	1.711594	3.209548	0.435909	C	-1.52052	-2.98671	-1.32362
O	1.079166	4.241691	0.581482	O	-0.82442	-3.9152	-1.69661
O	-1.07217	2.445149	0.72314	O	1.194897	-1.9487	-1.52341
C	-2.7731	0.341756	1.879866	C	2.164222	2.133405	-0.36501
C	4.484824	-0.16614	-0.12125	C	-4.48705	-0.02195	0.0211
C	5.428325	-0.10489	-1.16202	C	-5.42716	-0.39307	0.998475
C	6.375388	-1.10609	-1.34866	C	-6.42836	0.477399	1.416218
C	6.39122	-2.21237	-0.49382	C	-6.50438	1.760596	0.866308
C	5.454907	-2.29896	0.542518	C	-5.57307	2.154613	-0.10091
C	4.517213	-1.28612	0.724362	C	-4.58067	1.27121	-0.51703
O	7.335412	-3.17282	-0.72113	O	-7.50076	2.581368	1.313294
C	-3.92806	-2.66633	0.070679	C	5.078204	0.460084	-1.06624
C	-5.05226	-3.25873	0.902394	C	6.133604	1.545224	-1.2181
O	-2.92678	-3.31212	-0.19224	O	4.786953	-0.27262	-1.99381
H	-2.16173	-1.03987	-1.25838	H	3.000369	-1.20287	-0.17208
H	-4.7908	-0.70938	0.252017	H	4.45309	1.309178	0.795604
H	-3.05285	2.723153	-3.36152	H	1.742641	-1.76218	4.018256
H	-4.4793	2.103473	-4.21354	H	3.378399	-2.07391	4.623807
H	-4.64477	2.80547	-2.59452	H	2.772625	-0.41228	4.524746
H	-4.85586	-0.04529	4.345059	H	3.579123	4.909914	-1.28132
H	-3.31377	0.398001	5.069664	H	1.86455	5.108369	-1.6196
H	-4.08318	1.491541	3.903725	H	2.401129	4.773943	0.039458
H	-2.92655	-1.30276	3.17467	H	2.62735	2.74488	-2.31157
H	-3.1222	0.226722	-3.27848	H	3.301385	-2.26754	2.133674

H	-5.78116	0.611498	-1.82453	H	4.846236	0.179601	3.112408
H	-5.57518	-0.09551	-3.42577	H	5.414979	-1.48754	3.14849
H	-5.81558	-1.86966	-1.68592	H	6.355828	-0.24746	1.195109
H	-4.24798	-1.96677	-2.4835	H	5.364341	-1.60444	0.665431
H	-3.67038	1.540685	-0.57438	H	2.402986	0.648508	2.20487
H	-2.11757	1.454113	-1.37062	H	1.42683	-0.72816	1.710041
H	0.216938	-1.86697	0.408408	H	-0.32913	1.981643	0.064417
H	-1.92742	-1.42477	1.097619	H	2.405258	0.46259	-1.66603
H	5.011838	2.500816	-0.04914	H	-4.85874	-2.60913	-0.73529
H	3.459909	4.247977	0.233622	H	-3.20585	-4.14016	-1.41717
H	-2.96777	1.399346	1.728508	H	1.999432	2.455083	0.666111
H	5.406764	0.735296	-1.85122	H	-5.36036	-1.3776	1.45408
H	7.096728	-1.05461	-2.15794	H	-7.14737	0.185516	2.175014
H	5.463833	-3.15624	1.213481	H	-5.62902	3.151009	-0.53606
H	3.808294	-1.36528	1.54172	H	-3.87608	1.590116	-1.27781
H	7.226901	-3.8841	-0.07073	H	-7.43569	3.435552	0.858507
H	-6.02949	-3.08567	0.436782	H	6.660674	1.422398	-2.16665
H	-4.89037	-4.32908	1.046804	H	5.642596	2.526817	-1.20128
H	-5.07388	-2.75914	1.879877	H	6.848991	1.531459	-0.38757
Free Energy = -1476.752376 Hartree				Free Energy = -1476.751194 Hartree			
**Conf. G**	**X axis(Å)**	**Y axis(Å)**	**Z axis(Å)**	**Conf. H**	**X axis(Å)**	**Y axis(Å)**	**Z axis(Å)**
C	-7.12686	-0.68075	0.350198	C	2.765524	-1.34731	4.036956
C	-3.16844	2.812872	3.849718	C	2.578199	4.525381	-1.05811
C	-2.77416	1.555513	3.12796	C	2.458824	3.048567	-1.30847
C	-5.74671	-0.76221	-0.31199	C	3.318868	-1.24252	2.611273
C	-5.42846	-2.18065	-0.80172	C	4.778041	-0.76095	2.601871
C	-4.03967	-2.24305	-1.44273	C	5.323146	-0.61606	1.176132
C	-2.92584	-1.75671	-0.47821	C	4.433504	0.32159	0.31628
C	-3.21972	-0.31399	0.031767	C	2.97969	-0.2069	0.288543
C	-4.64107	-0.27371	0.637016	C	2.449764	-0.32658	1.730938
C	0.257198	-0.31742	1.032708	C	-0.41565	0.943601	-0.24965
C	-0.77504	0.397419	0.550902	C	0.608167	0.152822	-0.61673
C	-2.18001	0.176366	1.098651	C	2.051385	0.644928	-0.65057
O	1.551849	-0.16448	0.647033	O	-1.72504	0.557882	-0.22071
C	1.875453	0.767736	-0.28208	C	-2.06667	-0.70785	-0.56432
C	0.907388	1.58589	-0.85027	C	-1.11145	-1.62426	-0.97932
C	-0.51467	1.436614	-0.47123	C	0.313184	-1.22943	-1.07583
C	3.276486	0.830081	-0.6001	C	-3.47107	-0.99971	-0.44692
C	3.623398	1.790095	-1.51241	C	-3.82885	-2.28051	-0.77118
N	2.705369	2.619972	-2.07103	N	-2.91731	-3.20525	-1.16677
C	1.30888	2.602714	-1.83218	C	-1.52287	-2.9942	-1.31506
O	0.602817	3.395563	-2.43202	O	-0.82796	-3.92551	-1.68317
O	-1.42733	2.098919	-0.96435	O	1.196167	-1.96365	-1.50605

C	-2.61203	1.448245	1.806594	C	2.164903	2.128664	-0.38353
C	4.30237	-0.05515	0.005933	C	-4.48484	-0.01853	0.015015
C	4.137047	-1.44812	0.069665	C	-4.58136	1.272324	-0.53672
C	5.133042	-2.26637	0.595176	C	-5.56576	2.162219	-0.12631
C	6.32606	-1.70782	1.067363	C	-6.49059	1.779898	0.852862
C	6.509411	-0.32267	1.014713	C	-6.41357	0.502784	1.414785
C	5.503975	0.485942	0.495274	C	-5.41595	-0.37864	1.000193
O	7.339047	-2.45944	1.591437	O	-7.43635	2.696006	1.217079
C	-1.57366	-1.98277	-1.16151	C	5.07972	0.445666	-1.06412
C	-1.28917	-1.25912	-2.46981	C	6.133993	1.530308	-1.22682
O	-0.77731	-2.79108	-0.71206	O	4.791367	-0.29866	-1.98329
H	-3.18737	0.38446	-0.81197	H	2.999662	-1.20607	-0.15665
H	-2.91472	-2.42681	0.392994	H	4.45187	1.315224	0.787451
H	-7.35639	0.343197	0.668251	H	1.734832	-1.7217	4.03754
H	-7.91731	-1.00363	-0.33813	H	3.369392	-2.02806	4.649157
H	-7.17572	-1.3239	1.23858	H	2.764637	-0.36722	4.532107
H	-2.40575	3.105593	4.58438	H	1.858544	5.089999	-1.6663
H	-3.30709	3.647686	3.154818	H	2.401314	4.773599	-0.00584
H	-4.10433	2.675521	4.408495	H	3.574668	4.897929	-1.33199
H	-2.61297	0.674993	3.754448	H	2.624297	2.721141	-2.33675
H	-5.75185	-0.09872	-1.19179	H	3.297215	-2.24715	2.16122
H	-5.47448	-2.87768	0.048784	H	4.840174	0.210323	3.116854
H	-6.18627	-2.51569	-1.52261	H	5.408929	-1.45632	3.171805
H	-3.81522	-3.268	-1.76586	H	6.353243	-0.23767	1.206671
H	-4.03722	-1.61868	-2.34665	H	5.362084	-1.59992	0.689988
H	-4.65875	-0.89035	1.550545	H	2.39845	0.669394	2.20041
H	-4.86623	0.747989	0.960259	H	1.423345	-0.7124	1.718057
H	0.173925	-1.11075	1.765948	H	-0.3283	1.979991	0.049633
H	-2.10991	-0.61561	1.859018	H	2.407615	0.445231	-1.66789
H	4.649383	1.918943	-1.83924	H	-4.8623	-2.60772	-0.73684
H	2.988714	3.312961	-2.75421	H	-3.21044	-4.14414	-1.4111
H	-2.75651	2.317128	1.16688	H	2.001206	2.460197	0.644624
H	3.224512	-1.90214	-0.30226	H	-3.88009	1.579571	-1.30545
H	4.984595	-3.34421	0.630691	H	-5.63952	3.155333	-0.55802
H	7.432526	0.102048	1.396045	H	-7.12065	0.200813	2.185411
H	5.645682	1.563604	0.486823	H	-5.3496	-1.35807	1.466591
H	7.081068	-3.39431	1.574564	H	-8.00711	2.308543	1.898868
H	-1.8214	-1.76662	-3.28483	H	6.847506	1.52758	-0.39455
H	-1.6234	-0.21758	-2.46056	H	6.663382	1.396626	-2.17262
H	-0.21905	-1.30906	-2.6828	H	5.641668	2.511358	-1.22307
Free Energy = -1476.751048 Hartree				Free Energy = -1476.75102 Hartree			
**Conf. I**	**X axis(Å)**	**Y axis(Å)**	**Z axis(Å)**	**Conf. J**	**X axis(Å)**	**Y axis(Å)**	**Z axis(Å)**
C	-7.1304	-0.65646	0.372565	C	7.017049	0.781644	0.054628

C	-3.138	2.790319	3.876191	C	2.903125	-3.46553	3.319606
C	-2.75275	1.53638	3.143656	C	2.758109	-2.05259	2.831641
C	-5.7541	-0.74113	-0.29722	C	5.617191	0.642743	-0.55436
C	-5.44599	-2.1581	-0.79756	C	5.592675	-0.36779	-1.70964
C	-4.06079	-2.22383	-1.44595	C	4.186232	-0.50701	-2.29938
C	-2.93961	-1.74955	-0.48408	C	3.136428	-0.91659	-1.22847
C	-3.22303	-0.3087	0.036613	C	3.147926	0.084929	-0.04659
C	-4.6411	-0.26445	0.649204	C	4.577953	0.24878	0.507139
C	0.260764	-0.32968	1.025574	C	-0.2839	-0.71486	0.782455
C	-0.77193	0.388061	0.548781	C	0.742654	0.146736	0.863595
C	-2.17536	0.16893	1.101548	C	2.177589	-0.26754	1.145603
O	1.554768	-0.17614	0.637552	O	-1.57427	-0.35403	0.522421
C	1.876177	0.756058	-0.29188	C	-1.88779	0.950427	0.328428
C	0.907327	1.574969	-0.8575	C	-0.93221	1.949654	0.446623
C	-0.51312	1.43048	-0.47035	C	0.468384	1.603262	0.761409
C	3.276712	0.818556	-0.61257	C	-3.27441	1.192248	0.029666
C	3.621494	1.775201	-1.52894	C	-3.61559	2.50769	-0.13329
N	2.702099	2.603503	-2.08794	N	-2.70634	3.508618	-0.01506
C	1.306487	2.588839	-1.84339	C	-1.3252	3.352826	0.262959
O	0.599285	3.380802	-2.44307	O	-0.6247	4.348943	0.321258
O	-1.42547	2.099787	-0.95453	O	1.357868	2.427048	0.955629
C	-2.59778	1.437746	1.82075	C	2.311088	-1.69314	1.624445
C	4.304102	-0.06185	-0.00184	C	-4.29672	0.121571	-0.08021
C	5.495809	0.485215	0.495435	C	-4.09959	-1.01504	-0.88003
C	6.502028	-0.32366	1.021772	C	-5.08991	-1.98512	-1.0091
C	6.325684	-1.70913	1.071139	C	-6.31027	-1.83705	-0.34057
C	5.139022	-2.2741	0.588349	C	-6.52684	-0.71278	0.461595
C	4.146681	-1.45921	0.058934	C	-5.52654	0.245448	0.589409
O	7.266256	-2.55945	1.579044	O	-7.31973	-2.75388	-0.42966
C	-1.59159	-1.97756	-1.17491	C	1.796128	-1.00306	-1.96222
C	-1.31067	-1.24943	-2.48159	C	1.331493	-2.38802	-2.38458
O	-0.79591	-2.79001	-0.73228	O	1.158479	-0.00648	-2.25046
H	-3.19103	0.395095	-0.80267	H	2.823612	1.054406	-0.43559
H	-2.92749	-2.42525	0.382767	H	3.410094	-1.91695	-0.86502
H	-7.35273	0.366638	0.698405	H	7.02787	1.526055	0.859579
H	-7.9261	-0.97059	-0.31377	H	7.358733	-0.17204	0.47811
H	-7.17825	-1.30509	1.257012	H	7.750271	1.092773	-0.69942
H	-2.37047	3.074696	4.609114	H	2.294275	-3.63947	4.217114
H	-3.2766	3.630548	3.187816	H	2.598619	-4.19108	2.557342
H	-4.0717	2.653043	4.438634	H	3.942196	-3.6841	3.600413
H	-2.59252	0.650692	3.763032	H	3.044581	-1.2708	3.53745
H	-5.76023	-0.07209	-1.17282	H	5.323817	1.622413	-0.96277
H	-5.49156	-2.86031	0.048693	H	6.294279	-0.06269	-2.49776

H	-6.20926	-2.48438	-1.51672	H	5.938996	-1.34666	-1.34356
H	-3.84346	-3.24791	-1.77657	H	4.189053	-1.2426	-3.11531
H	-4.05954	-1.59388	-2.34601	H	3.870072	0.448373	-2.73772
H	-4.65765	-0.88655	1.559033	H	4.558943	1.012376	1.295596
H	-4.85923	0.756453	0.979759	H	4.894529	-0.68963	0.987591
H	0.178881	-1.12518	1.756603	H	-0.22037	-1.78717	0.918132
H	-2.10507	-0.62881	1.855793	H	2.497163	0.393126	1.961576
H	4.646702	1.902571	-1.85878	H	-4.62969	2.805331	-0.37672
H	2.983273	3.293108	-2.77539	H	-2.9827	4.473229	-0.15709
H	-2.74157	2.311834	1.187981	H	2.030659	-2.48481	0.92639
H	5.633699	1.563326	0.490561	H	-3.16558	-1.13839	-1.41818
H	7.415673	0.125553	1.407134	H	-4.91744	-2.85573	-1.6397
H	5.015656	-3.3516	0.629561	H	-7.4721	-0.613	0.985548
H	3.238166	-1.91497	-0.32088	H	-5.69478	1.101192	1.238058
H	8.030942	-2.04396	1.879467	H	-7.03866	-3.47845	-1.00985
H	-1.64218	-0.20706	-2.46704	H	2.127511	-2.91977	-2.9205
H	-0.24155	-1.30141	-2.69902	H	1.093023	-2.99002	-1.49759
H	-1.84741	-1.75256	-3.29636	H	0.44588	-2.30997	-3.01893
Free Energy = -1476.750812 Hartree				Free Energy = -1476.750698 Hartree			

Table S4. The Cartesian coordinates of the optimized conformers (above 1% population, from 34 conformers in total) for (9*R*,10*R*,12*S*,15*S*)-**7**.

Conf. A	X axis(Å)	Y axis(Å)	Z axis(Å)	Conf. B	X axis(Å)	Y axis(Å)	Z axis(Å)
C	3.037551	-1.97176	3.875242	C	2.967991	-1.62692	4.07586
C	2.865529	-3.37983	-3.2748	C	2.920126	-3.68566	-2.89548
C	2.721165	-2.01147	-2.67459	C	2.764834	-2.26648	-2.43171
C	3.415365	-0.96862	2.77954	C	3.36177	-0.72098	2.903757
C	4.912922	-1.03029	2.438507	C	4.865263	-0.80629	2.594997
C	5.284548	-0.03625	1.330752	C	5.253001	0.088083	1.410669
C	4.439735	-0.25458	0.053156	C	4.433767	-0.25044	0.142797
C	2.925553	-0.18009	0.386807	C	2.912951	-0.15116	0.440666
C	2.582342	-1.18412	1.503818	C	2.551689	-1.05145	1.637903
C	-0.4384	-0.78234	-0.61274	C	-0.43279	-0.83961	-0.54617
C	0.60206	0.069751	-0.6218	C	0.610224	0.00481	-0.63221
C	2.03497	-0.36312	-0.8968	C	2.046228	-0.4557	-0.83542
O	-1.73248	-0.42201	-0.37488	O	-1.73021	-0.453	-0.37689
C	-2.04206	0.872551	-0.1207	C	-2.03972	0.86233	-0.27305
C	-1.06688	1.861006	-0.12711	C	-1.05969	1.842165	-0.36068
C	0.339984	1.516196	-0.41504	C	0.350314	1.465646	-0.58641
C	-3.43812	1.117028	0.124795	C	-3.43569	1.133769	-0.05713
C	-3.76779	2.425636	0.356699	C	-3.75822	2.458461	0.069496

N	-2.83952	3.415896	0.354026	N	-2.82271	3.439508	0.001933
C	-1.44807	3.254973	0.140123	C	-1.43486	3.25583	-0.21662
O	-0.72886	4.237387	0.199156	O	-0.71348	4.237602	-0.25786
O	1.242742	2.347286	-0.49625	O	1.258239	2.281089	-0.73756
C	2.146156	-1.74519	-1.49804	C	2.164601	-1.88967	-1.29877
C	-4.48178	0.061376	0.104993	C	-4.47837	0.083052	0.055941
C	-5.67525	0.262358	-0.61066	C	-5.41102	0.125755	1.107256
C	-6.69719	-0.68085	-0.60441	C	-6.43582	-0.80857	1.211854
C	-6.54039	-1.8678	0.117996	C	-6.54403	-1.82736	0.260261
C	-5.35677	-2.09274	0.829873	C	-5.62068	-1.89455	-0.78919
C	-4.34446	-1.13706	0.822854	C	-4.60425	-0.94833	-0.8879
O	-7.56905	-2.7653	0.08777	O	-7.5627	-2.72446	0.409199
C	4.867687	0.710963	-1.06153	C	4.878118	0.608029	-1.05004
C	4.676176	2.202614	-0.85495	C	4.701127	2.113816	-0.97872
O	5.350215	0.276184	-2.09486	O	5.359686	0.078319	-2.03872
H	2.715496	0.827306	0.764824	H	2.693291	0.885719	0.721612
H	4.660455	-1.25427	-0.34038	H	4.662456	-1.2818	-0.15221
H	1.974	-1.89914	4.133514	H	1.899535	-1.53861	4.307273
H	3.23166	-3.00213	3.54969	H	3.172678	-2.68045	3.844525
H	3.616678	-1.79818	4.790214	H	3.529403	-1.37102	4.982515
H	2.432417	-4.15453	-2.63232	H	2.466053	-4.39541	-2.19496
H	2.374287	-3.43611	-4.25567	H	2.45689	-3.83449	-3.88038
H	3.922905	-3.6249	-3.4408	H	3.980776	-3.94688	-3.00731
H	3.143538	-1.18885	-3.2519	H	3.202813	-1.50177	-3.07327
H	3.199102	0.044423	3.15497	H	3.136493	0.319976	3.185856
H	5.1658	-2.05183	2.116261	H	5.126084	-1.85099	2.367089
H	5.513554	-0.82855	3.335556	H	5.44982	-0.52463	3.481056
H	6.350517	-0.12499	1.084676	H	6.324208	-0.01385	1.19401
H	5.131476	0.984914	1.705295	H	5.085434	1.137269	1.68913
H	2.757192	-2.21003	1.147542	H	2.735036	-2.1044	1.378234
H	1.513472	-1.11488	1.746684	H	1.478565	-0.96466	1.854875
H	-0.38086	-1.84517	-0.80802	H	-0.37518	-1.91792	-0.61657
H	2.409753	0.354895	-1.63623	H	2.43615	0.186888	-1.63412
H	-4.78911	2.724896	0.565201	H	-4.78278	2.781064	0.218815
H	-3.11008	4.373542	0.546549	H	-3.09203	4.41272	0.089564
H	1.757483	-2.57933	-0.91133	H	1.762485	-2.66406	-0.6431
H	-5.79657	1.167436	-1.20008	H	-5.31985	0.895564	1.869155
H	-7.61334	-0.52151	-1.16398	H	-7.14893	-0.77266	2.029201
H	-5.2297	-3.01263	1.39796	H	-5.70189	-2.68279	-1.5357
H	-3.44034	-1.32356	1.392838	H	-3.9069	-1.00858	-1.71683
H	-7.33007	-3.5411	0.618621	H	-7.51854	-3.37704	-0.307
H	5.108554	2.737212	-1.70347	H	5.143517	2.565847	-1.86905
H	3.608414	2.442908	-0.77547	H	3.635015	2.369649	-0.92964

H	5.151821	2.544376	0.071688	H	5.174854	2.532879	-0.08343
Free Energy = -1476.753584 Hartree				Free Energy = -1476.753286 Hartree			
**Conf. C**	**X axis(Å)**	**Y axis(Å)**	**Z axis(Å)**	**Conf. D**	**X axis(Å)**	**Y axis(Å)**	**Z axis(Å)**
C	4.706332	1.875298	-2.99124	C	-6.86131	-1.54118	-0.91734
C	3.884902	0.977562	4.105161	C	-2.72877	-3.68763	3.395372
C	3.176472	0.16244	3.060214	C	-2.68148	-2.31528	2.78663
C	4.175298	0.569742	-2.38808	C	-5.47492	-1.00824	-1.29694
C	5.303512	-0.44149	-2.13893	C	-5.53749	0.423641	-1.8395
C	4.774365	-1.74074	-1.52135	C	-4.14028	0.925337	-2.20636
C	3.978332	-1.48446	-0.21177	C	-3.13474	0.868599	-1.01759
C	2.843309	-0.46091	-0.44975	C	-3.07705	-0.55417	-0.39741
C	3.404512	0.826872	-1.08237	C	-4.50831	-1.06499	-0.10584
C	-0.37642	-0.67632	0.496119	C	0.176145	-1.01875	0.345094
C	0.592668	0.255701	0.566726	C	-0.79309	-0.21027	0.805254
C	2.014593	-0.21929	0.864964	C	-2.24464	-0.65085	0.932875
O	-1.69013	-0.42109	0.241148	O	1.482979	-0.65821	0.213718
C	-2.10577	0.857388	0.067417	C	1.87644	0.594598	0.541183
C	-1.21089	1.917394	0.117554	C	0.977401	1.52533	1.04579
C	0.235062	1.675212	0.337166	C	-0.44174	1.164237	1.225914
C	-3.52079	1.000457	-0.15101	C	3.277274	0.853267	0.342798
C	-3.95803	2.288601	-0.30481	C	3.695181	2.107003	0.698798
N	-3.11202	3.348508	-0.24895	N	2.841197	3.038764	1.196506
C	-1.7099	3.289696	-0.04982	C	1.446708	2.875894	1.378215
O	-1.07958	4.333251	-0.03386	O	0.790346	3.828146	1.768288
O	1.076976	2.570446	0.325481	O	-1.29795	1.932538	1.665853
C	2.721419	0.635797	1.895576	C	-2.31786	-2.04192	1.531145
C	-4.47574	-0.1357	-0.18266	C	4.234704	-0.14867	-0.18903
C	-4.24488	-1.28458	-0.95569	C	5.463398	-0.36764	0.457338
C	-5.17755	-2.31679	-1.00805	C	6.404378	-1.25857	-0.04844
C	-6.37319	-2.22114	-0.28747	C	6.126489	-1.96707	-1.22129
C	-6.62276	-1.08584	0.4895	C	4.905433	-1.77037	-1.87604
C	-5.67934	-0.06523	0.540857	C	3.974667	-0.8702	-1.36494
O	-7.32607	-3.19985	-0.30014	O	7.078442	-2.83547	-1.67523
C	3.469664	-2.84215	0.276542	C	-1.81787	1.37396	-1.61928
C	4.405935	-3.6502	1.158686	C	-1.56869	2.870338	-1.55713
O	2.372992	-3.27403	-0.04037	O	-1.04669	0.620059	-2.19011
H	2.149822	-0.92698	-1.16194	H	-2.60689	-1.21335	-1.14162
H	4.676236	-1.08403	0.537775	H	-3.46676	1.579204	-0.24907
H	3.891358	2.579629	-3.19388	H	-6.80685	-2.57498	-0.55539
H	5.406371	2.368241	-2.30437	H	-7.31147	-0.93381	-0.12145
H	5.237024	1.69218	-3.93378	H	-7.54314	-1.52208	-1.77626
H	3.338144	0.96374	5.057988	H	-2.06231	-3.75997	4.265651
H	3.993308	2.021152	3.792277	H	-3.73839	-3.92941	3.754287

H	4.887183	0.578728	4.314325	H	-2.43123	-4.45903	2.676559
H	3.022341	-0.89269	3.301446	H	-2.96922	-1.48921	3.439348
H	3.477439	0.118629	-3.11243	H	-5.0695	-1.65033	-2.09567
H	6.047837	0.008445	-1.46439	H	-5.98418	1.081912	-1.07904
H	5.828112	-0.66812	-3.07696	H	-6.19118	0.472863	-2.72102
H	5.6057	-2.43115	-1.32788	H	-4.19694	1.952841	-2.58757
H	4.108994	-2.243	-2.2372	H	-3.7373	0.31124	-3.02338
H	4.082261	1.315917	-0.36757	H	-4.93061	-0.46779	0.718466
H	2.593082	1.539451	-1.2558	H	-4.4557	-2.0927	0.269278
H	-0.21412	-1.74145	0.619727	H	0.039773	-2.04082	0.015023
H	1.886677	-1.20954	1.314731	H	-2.6933	0.05386	1.642874
H	-5.00208	2.516004	-0.49023	H	4.728336	2.415546	0.58269
H	-3.46096	4.291422	-0.37665	H	3.172036	3.970202	1.4204
H	2.838249	1.689253	1.658186	H	-2.03854	-2.87048	0.874846
H	-3.32991	-1.37244	-1.53173	H	5.678082	0.156444	1.385124
H	-4.97833	-3.19651	-1.61755	H	7.349081	-1.42736	0.458511
H	-7.54686	-1.0269	1.055622	H	4.685138	-2.31566	-2.7922
H	-5.87054	0.79835	1.172528	H	3.03928	-0.71999	-1.8939
H	-7.02371	-3.92923	-0.86347	H	6.756893	-3.26282	-2.48438
H	4.027243	-4.66778	1.275995	H	-2.45516	3.428668	-1.88148
H	5.423867	-3.67192	0.753058	H	-1.36769	3.169467	-0.5215
H	4.469199	-3.17266	2.145745	H	-0.71773	3.131211	-2.19049
Free Energy = -1476.752717 Hartree				Free Energy = -1476.752712 Hartree			
**Conf. E**	**X axis(Å)**	**Y axis(Å)**	**Z axis(Å)**	**Conf. F**	**X axis(Å)**	**Y axis(Å)**	**Z axis(Å)**
C	-6.85358	-1.56303	-0.92685	C	4.000432	2.186035	-3.23
C	-2.7142	-3.71247	3.371131	C	3.89231	0.43948	4.136073
C	-2.67113	-2.3361	2.771181	C	3.162175	-0.24659	3.015816
C	-5.4717	-1.01503	-1.30147	C	3.773694	0.805767	-2.60273
C	-5.54597	0.422179	-1.82819	C	5.08808	0.027343	-2.44878
C	-4.15299	0.939236	-2.18981	C	4.858002	-1.3477	-1.81187
C	-3.14673	0.876843	-1.00204	C	4.12787	-1.24103	-0.44503
C	-3.07662	-0.55289	-0.39935	C	2.804147	-0.45305	-0.58729
C	-4.50358	-1.07786	-0.11186	C	3.062247	0.916747	-1.24333
C	0.17873	-1.0188	0.329902	C	-0.37737	-0.79435	0.433311
C	-0.78997	-0.21506	0.799667	C	0.596986	0.119846	0.59761
C	-2.24073	-0.6584	0.928133	C	2.029767	-0.38218	0.776632
O	1.48511	-0.65611	0.199308	O	-1.6995	-0.50916	0.265932
C	1.877635	0.594546	0.534741	C	-2.11574	0.781127	0.276562
C	0.979055	1.520865	1.047936	C	-1.21702	1.825468	0.44355
C	-0.43865	1.155859	1.231566	C	0.232375	1.557319	0.603284
C	3.278039	0.855633	0.335864	C	-3.53149	0.953128	0.084797
C	3.695322	2.107997	0.696534	C	-3.96349	2.252012	0.073174
N	2.841156	3.036381	1.200698	N	-3.1111	3.296538	0.229064

C	1.447543	2.87019	1.386394	C	-1.71163	3.209717	0.43545
O	0.791132	3.819369	1.783906	O	-1.07922	4.241881	0.580984
O	-1.29358	1.9186	1.683538	O	1.072136	2.445391	0.722825
C	-2.30969	-2.05365	1.517073	C	2.773089	0.342105	1.879952
C	4.234711	-0.14504	-0.19985	C	-4.48479	-0.16607	-0.12129
C	3.97931	-0.8542	-1.38819	C	-5.42813	-0.10512	-1.16223
C	4.903645	-1.75347	-1.90397	C	-6.37516	-1.10637	-1.34872
C	6.118644	-1.96591	-1.24127	C	-6.39113	-2.21239	-0.49355
C	6.392495	-1.27205	-0.05956	C	-5.45499	-2.29868	0.54296
C	5.453821	-0.37722	0.452753	C	-4.51732	-1.28579	0.724649
O	6.988955	-2.86166	-1.7949	O	-7.3353	-3.1729	-0.72072
C	-1.83463	1.401701	-1.59734	C	3.927165	-2.66683	0.070531
C	-1.59965	2.899574	-1.51792	C	5.051308	-3.26002	0.901786
O	-1.05577	0.661599	-2.17555	O	2.925477	-3.31207	-0.1922
H	-2.60278	-1.19956	-1.15211	H	2.161527	-1.03952	-1.25827
H	-3.48522	1.574896	-0.22481	H	4.790623	-0.71027	0.25236
H	-6.79065	-2.60019	-0.57604	H	3.053971	2.723409	-3.36092
H	-7.30817	-0.96785	-0.12427	H	4.645921	2.805128	-2.59391
H	-7.53605	-1.5402	-1.78517	H	4.480222	2.103351	-4.21301
H	-3.72279	-3.9591	3.729639	H	3.314513	0.398365	5.069613
H	-2.41558	-4.4785	2.647075	H	4.85655	-0.04446	4.344611
H	-2.04655	-3.78873	4.240188	H	4.083338	1.49217	3.903533
H	-2.96035	-1.51504	3.429565	H	2.927342	-1.30244	3.174647
H	-5.06201	-1.64505	-2.10758	H	3.122521	0.226947	-3.27815
H	-5.99776	1.068447	-1.06044	H	5.781566	0.610738	-1.82409
H	-6.20039	0.475749	-2.70892	H	5.575421	-0.09607	-3.42541
H	-4.21802	1.970567	-2.55917	H	5.815206	-1.87043	-1.6857
H	-3.74535	0.337901	-3.01397	H	4.247659	-1.96699	-2.48345
H	-4.92948	-0.49342	0.719747	H	3.671204	1.540325	-0.57388
H	-4.44299	-2.1094	0.251321	H	2.118344	1.454582	-1.37008
H	0.042683	-2.03803	-0.0089	H	-0.2169	-1.86678	0.408644
H	-2.6885	0.040307	1.64461	H	1.92737	-1.42452	1.097953
H	4.727818	2.41834	0.579236	H	-5.01183	2.500905	-0.04971
H	3.170822	3.967666	1.426846	H	-3.45993	4.248107	0.232863
H	-2.02877	-2.87712	0.855133	H	2.967399	1.399764	1.7286
H	3.047783	-0.69059	-1.92019	H	-5.40646	0.734891	-1.85165
H	4.705669	-2.29528	-2.82336	H	-7.09638	-1.05512	-2.15813
H	7.329489	-1.4398	0.468624	H	-5.46402	-3.15576	1.214177
H	5.667029	0.134521	1.387673	H	-3.80852	-1.3647	1.542138
H	7.782486	-2.91717	-1.23997	H	-7.2269	-3.88398	-0.07009
H	-1.39034	3.185761	-0.48022	H	5.073068	-2.76105	1.879596
H	-0.75721	3.177017	-2.15565	H	4.889148	-4.3304	1.045571
H	-2.4944	3.454077	-1.82545	H	6.02857	-3.0869	0.436281

Free Energy = -1476.752684 Hartree				Free Energy = -1476.752381 Hartree			
**Conf. G**	**X axis(Å)**	**Y axis(Å)**	**Z axis(Å)**	**Conf. H**	**X axis(Å)**	**Y axis(Å)**	**Z axis(Å)**
C	-2.77285	-1.38595	4.020208	C	7.126886	-0.68056	0.350134
C	-2.58089	4.535916	-1.01621	C	3.168337	2.812929	3.849672
C	-2.46015	3.06175	-1.28137	C	2.774092	1.555547	3.127937
C	-3.32375	-1.26686	2.594705	C	5.746718	-0.76212	-0.31201
C	-4.78293	-0.78529	2.587668	C	5.428525	-2.18058	-0.80169
C	-5.32573	-0.62556	1.162608	C	4.039725	-2.24308	-1.44266
C	-4.43441	0.320712	0.313881	C	2.925897	-1.75676	-0.47813
C	-2.98078	-0.20809	0.2832	C	3.21972	-0.31401	0.031781
C	-2.45319	-0.342	1.725175	C	4.641085	-0.27363	0.636997
C	0.41632	0.947115	-0.24203	C	-0.25721	-0.31747	1.032643
C	-0.60764	0.159317	-0.6148	C	0.775029	0.397386	0.550869
C	-2.05062	0.652174	-0.64647	C	2.179998	0.176345	1.098648
O	1.725673	0.560856	-0.21497	O	-1.55185	-0.16454	0.646947
C	2.066764	-0.70408	-0.56306	C	-1.87545	0.76771	-0.28213
C	1.111028	-1.61731	-0.98408	C	-0.90738	1.585875	-0.8503
C	-0.31256	-1.21978	-1.08341	C	0.514669	1.436589	-0.47125
C	3.470462	-0.99865	-0.4442	C	-3.27649	0.830082	-0.60014
C	3.826213	-2.27949	-0.77123	C	-3.6234	1.790123	-1.51243
N	2.913993	-3.2012	-1.1719	N	-2.70536	2.620005	-2.07103
C	1.520642	-2.98686	-1.32361	C	-1.30887	2.602726	-1.83219
O	0.824588	-3.91541	-1.69657	O	-0.6028	3.395581	-2.43201
O	-1.19492	-1.94917	-1.52299	O	1.427335	2.098888	-0.96437
C	-2.16422	2.133259	-0.36568	C	2.611984	1.448247	1.806571
C	4.486977	-0.02193	0.021144	C	-4.30238	-0.05514	0.005893
C	5.427041	-0.39291	0.998623	C	-4.1371	-1.44811	0.069573
C	6.428196	0.477637	1.416322	C	-5.1331	-2.26635	0.595097
C	6.504217	1.760769	0.866258	C	-6.32608	-1.70778	1.06735
C	5.572946	2.15465	-0.10105	C	-6.50939	-0.32262	1.014754
C	4.580586	1.271174	-0.51712	C	-5.50395	0.485975	0.495303
O	7.500571	2.581609	1.31318	O	-7.33907	-2.45939	1.591442
C	-5.07832	0.459636	-1.06615	C	1.573699	-1.98291	-1.16136
C	-6.13383	1.544627	-1.21828	C	1.289124	-1.25932	-2.46968
O	-4.7871	-0.27339	-1.99347	O	0.777412	-2.79125	-0.71186
H	-3.0004	-1.20294	-0.17157	H	3.187324	0.384405	-0.81199
H	-4.45311	1.309395	0.795379	H	2.914846	-2.42683	0.39311
H	-1.74234	-1.76079	4.018869	H	7.356369	0.343406	0.668144
H	-2.77239	-0.4108	4.525	H	7.175794	-1.32368	1.238538
H	-3.37805	-2.07244	4.624609	H	7.917332	-1.00343	-0.3382
H	-1.86425	5.107801	-1.62121	H	2.405637	3.105647	4.58432
H	-3.57886	4.909564	-1.28302	H	4.104219	2.675611	4.408463
H	-2.40099	4.773967	0.037909	H	3.306981	3.647732	3.154758

H	-2.62711	2.744146	-2.31247	H	2.612914	0.675036	3.754439
H	-3.30122	-2.26685	2.134553	H	5.751806	-0.09866	-1.19184
H	-4.84604	0.180592	3.112567	H	5.474608	-2.87758	0.048837
H	-5.41476	-1.48654	3.149262	H	6.186335	-2.51562	-1.52259
H	-6.35577	-0.24718	1.195505	H	3.815311	-3.26805	-1.76574
H	-5.36425	-1.60429	0.666224	H	4.037212	-1.61874	-2.3466
H	-2.40283	0.649214	2.20473	H	4.658813	-0.89023	1.550549
H	-1.42672	-0.72762	1.710264	H	4.866201	0.748091	0.960199
H	0.32902	1.981558	0.064	H	-0.17394	-1.11082	1.765864
H	-2.40537	0.462018	-1.66613	H	2.109886	-0.61562	1.859025
H	4.858772	-2.60921	-0.73494	H	-4.64938	1.918994	-1.83924
H	3.205976	-4.14032	-1.41683	H	-2.9887	3.313005	-2.7542
H	-1.99947	2.455244	0.665355	H	2.756442	2.317121	1.166841
H	5.360223	-1.37737	1.454353	H	-3.22459	-1.90215	-0.3024
H	7.147164	0.185864	2.175198	H	-4.98469	-3.34419	0.630572
H	5.628882	3.151003	-0.5363	H	-7.43247	0.102114	1.39614
H	3.876026	1.589978	-1.27796	H	-5.64562	1.563642	0.486894
H	7.435443	3.435769	0.858355	H	-7.08112	-3.39426	1.574528
H	-5.64293	2.526276	-1.20178	H	1.623371	-0.21779	-2.46051
H	-6.66097	1.421457	-2.16675	H	1.821282	-1.76688	-3.2847
H	-6.84916	1.531021	-0.3877	H	0.218984	-1.30925	-2.68259
Free Energy = -1476.75119 Hartree				Free Energy = -1476.751048 Hartree			
**Conf. I**	**X axis(Å)**	**Y axis(Å)**	**Z axis(Å)**	**Conf. J**	**X axis(Å)**	**Y axis(Å)**	**Z axis(Å)**
C	-2.7651	-1.34655	4.037269	C	7.130376	-0.65661	0.372737
C	-2.57825	4.525089	-1.05906	C	3.137912	2.790122	3.876405
C	-2.45896	3.04822	-1.30913	C	2.752693	1.536217	3.143799
C	-3.31861	-1.24199	2.611637	C	5.754108	-0.7412	-0.29711
C	-4.77776	-0.76039	2.602294	C	5.445963	-2.15812	-0.79754
C	-5.32301	-0.6158	1.17658	C	4.060825	-2.22374	-1.44605
C	-4.43342	0.321621	0.316414	C	2.93957	-1.74947	-0.48427
C	-2.97963	-0.20691	0.288663	C	3.223031	-0.30869	0.036621
C	-2.44958	-0.32625	1.731027	C	4.641076	-0.26453	0.649274
C	0.415595	0.943537	-0.25002	C	-0.2608	-0.32963	1.025722
C	-0.6082	0.152651	-0.61694	C	0.771906	0.388048	0.548874
C	-2.05143	0.644741	-0.65075	C	2.175351	0.168874	1.1016
O	1.724998	0.557854	-0.22106	O	-1.5548	-0.17605	0.637722
C	2.066666	-0.7079	-0.56445	C	-1.87619	0.75608	-0.29178
C	1.111472	-1.6244	-0.97934	C	-0.90731	1.57494	-0.85744
C	-0.31319	-1.22966	-1.07585	C	0.513133	1.430457	-0.47028
C	3.471067	-0.99976	-0.44694	C	-3.2767	0.818522	-0.61253
C	3.828856	-2.28059	-0.771	C	-3.62144	1.775084	-1.52902
N	2.917329	-3.20538	-1.16654	N	-2.70203	2.603343	-2.08804
C	1.522927	-2.99437	-1.31489	C	-1.30643	2.588749	-1.84341

O	0.828022	-3.92573	-1.68292	O	-0.59923	3.380686	-2.44313
O	-1.19616	-1.96396	-1.50595	O	1.425502	2.099732	-0.95447
C	-2.16494	2.128512	-0.38403	C	2.59776	1.437649	1.820887
C	4.484793	-0.01849	0.014968	C	-4.30409	-0.06187	-0.00181
C	4.581249	1.272357	-0.53679	C	-4.14662	-1.45922	0.059128
C	5.565556	2.162337	-0.12634	C	-5.13897	-2.2741	0.588533
C	6.490378	1.780115	0.85288	C	-6.32571	-1.70913	1.071149
C	6.413438	0.502987	1.414798	C	-6.5021	-0.32367	1.021642
C	5.415905	-0.37852	1.000175	C	-5.49586	0.48519	0.49532
O	7.436026	2.6963	1.21714	O	-7.2663	-2.55945	1.579032
C	-5.07976	0.445369	-1.06394	C	1.591598	-1.97727	-1.17529
C	-6.13396	1.530063	-1.22681	C	1.310887	-1.24893	-2.4819
O	-4.79161	-0.29927	-1.98292	O	0.795776	-2.78967	-0.73283
H	-2.99963	-1.20617	-0.15632	H	3.191088	0.395201	-0.80258
H	-4.45171	1.315366	0.787362	H	2.927286	-2.42527	0.382494
H	-1.73448	-1.72114	4.037796	H	7.352729	0.366469	0.698626
H	-2.76395	-0.36635	4.532197	H	7.178164	-1.30528	1.257163
H	-3.36901	-2.02704	4.649707	H	7.926095	-0.97074	-0.31357
H	-1.85864	5.089562	-1.66744	H	2.37037	3.074447	4.60933
H	-3.57473	4.897634	-1.33291	H	4.071599	2.652827	4.438867
H	-2.40124	4.773514	-0.00686	H	3.276516	3.630389	3.188077
H	-2.62458	2.720565	-2.33731	H	2.592464	0.650497	3.763135
H	-3.29702	-2.2467	2.161745	H	5.760304	-0.07211	-1.17268
H	-4.83982	0.211003	3.117063	H	5.4914	-2.86038	0.048692
H	-5.40862	-1.45561	3.172443	H	6.209269	-2.48443	-1.51665
H	-6.35308	-0.23734	1.207149	H	3.843448	-3.24779	-1.77674
H	-5.36206	-1.59977	0.690673	H	4.0597	-1.59375	-2.34608
H	-2.39821	0.669829	2.200277	H	4.657557	-0.88668	1.559072
H	-1.42316	-0.71207	1.718135	H	4.859214	0.756349	0.979894
H	0.32821	1.979974	0.049085	H	-0.17894	-1.12508	1.756803
H	-2.4078	0.444786	-1.66797	H	2.105085	-0.62891	1.855811
H	4.86228	-2.60785	-0.73653	H	-4.64662	1.902393	-1.85897
H	3.210511	-4.1443	-1.41071	H	-2.98321	3.292903	-2.77553
H	-2.00114	2.460283	0.644042	H	2.741547	2.311775	1.188166
H	3.880006	1.579545	-1.30557	H	-3.23804	-1.91498	-0.32054
H	5.639243	3.15545	-0.55806	H	-5.01556	-3.35158	0.629877
H	7.120516	0.201084	2.18545	H	-7.4158	0.125548	1.406876
H	5.349627	-1.35794	1.466589	H	-5.6338	1.563295	0.490361
H	8.006766	2.308904	1.898978	H	-8.03103	-2.04396	1.879348
H	-6.84763	1.527268	-0.39466	H	1.642094	-0.20646	-2.46701
H	-5.64162	2.511103	-1.22285	H	1.848019	-1.75168	-3.29663
H	-6.66317	1.396419	-2.17271	H	0.241841	-1.30112	-2.69967
Free Energy = -1476.751019 Hartree				Free Energy = -1476.750811 Hartree			

Table S5. The Cartesian coordinates of the optimized conformers (above 2% population, from 25 conformers in total) for (9*S**,10*R**,12*S**,15*S**)-**7**.

Conf. A	X axis(Å)	Y axis(Å)	Z axis(Å)	Conf. B	X axis(Å)	Y axis(Å)	Z axis(Å)
C	-3.10409	-0.58157	4.323793	C	-2.91863	0.623095	4.359637
C	-3.08506	1.193717	-4.28471	C	-3.20561	-0.10378	-4.40561
C	-2.92034	1.306362	-2.79565	C	-2.9941	0.434322	-3.01895
C	-3.32541	-1.066	2.886425	C	-3.2068	-0.23318	3.121311
C	-4.81559	-1.20286	2.548624	C	-4.71153	-0.42084	2.888518
C	-5.01033	-1.69561	1.112647	C	-4.97011	-1.2899	1.655497
C	-4.32208	-0.78452	0.056591	C	-4.31627	-0.72252	0.363384
C	-2.82975	-0.55124	0.393342	C	-2.80854	-0.43574	0.566105
C	-2.65471	-0.13062	1.86957	C	-2.56713	0.370036	1.862035
C	0.177271	-0.369	-0.4193	C	0.179524	-0.48517	-0.38074
C	-0.67157	0.660381	-0.26131	C	-0.68046	0.543457	-0.46579
C	-2.16834	0.53317	-0.5183	C	-2.18174	0.34354	-0.63572
O	1.528433	-0.29015	-0.24158	O	1.533072	-0.35258	-0.26281
C	2.097246	0.891196	0.104584	C	2.090856	0.882879	-0.22273
C	1.333699	2.035559	0.294591	C	1.31433	2.031673	-0.29999
C	-0.13562	1.990015	0.129484	C	-0.15511	1.933152	-0.43597
C	3.529676	0.845621	0.235155	C	3.522517	0.884792	-0.07628
C	4.123537	2.035121	0.561513	C	4.100095	2.124314	-0.01072
N	3.406665	3.172915	0.745755	N	3.36876	3.265855	-0.07601
C	1.998141	3.296983	0.649402	C	1.962553	3.348408	-0.23217
O	1.491483	4.385982	0.858625	O	1.444735	4.450505	-0.29209
O	-0.87862	2.95185	0.296252	O	-0.90698	2.899615	-0.5118
C	-2.38632	0.366564	-2.00807	C	-2.44226	-0.24415	-2.00714
C	4.344872	-0.37423	0.007649	C	4.347555	-0.34465	0.032468
C	5.494908	-0.31415	-0.79888	C	4.221535	-1.41076	-0.87205
C	6.312002	-1.42347	-0.98886	C	5.038531	-2.53419	-0.77973
C	5.984816	-2.63758	-0.37741	C	6.010901	-2.61562	0.223252
C	4.839501	-2.72259	0.42207	C	6.153088	-1.56482	1.134643
C	4.034401	-1.60277	0.611652	C	5.325036	-0.45164	1.037468
O	6.813787	-3.70004	-0.59983	O	6.841886	-3.69039	0.364235
C	-4.53481	-1.49339	-1.28579	C	-4.59343	-1.76945	-0.72134
C	-5.73917	-1.07061	-2.10591	C	-5.82853	-1.56378	-1.57853
O	-3.81366	-2.413	-1.63415	O	-3.89762	-2.76506	-0.82986
H	-2.32243	-1.51268	0.232611	H	-2.31559	-1.41394	0.654166
H	-4.84458	0.18301	0.042399	H	-4.82872	0.214417	0.100778
H	-2.03574	-0.51189	4.560995	H	-1.84035	0.729385	4.52846
H	-3.54231	0.413214	4.47553	H	-3.33705	1.631416	4.246615
H	-3.56557	-1.2634	5.0484	H	-3.35771	0.178673	5.261036
H	-2.73796	0.22403	-4.65704	H	-2.648	0.48526	-5.14656

H	-4.13524	1.316354	-4.58336	H	-2.8823	-1.14701	-4.48704
H	-2.52046	1.980292	-4.80367	H	-4.26291	-0.05213	-4.69952
H	-3.25112	2.242699	-2.3427	H	-3.30592	1.466387	-2.84839
H	-2.86793	-2.06397	2.787862	H	-2.768	-1.23076	3.284959
H	-5.30425	-1.89762	3.245001	H	-5.18144	-0.88137	3.76801
H	-5.30852	-0.22762	2.68061	H	-5.18393	0.5648	2.759459
H	-6.08066	-1.77978	0.882468	H	-6.04966	-1.4126	1.496831
H	-4.58933	-2.70528	1.015378	H	-4.56289	-2.29498	1.828762
H	-1.58468	-0.05186	2.097086	H	-1.48778	0.482245	2.020645
H	-3.06574	0.882394	2.000168	H	-2.95958	1.389135	1.723432
H	-0.10201	-1.37599	-0.7079	H	-0.09185	-1.53466	-0.39537
H	-2.58882	1.503873	-0.23133	H	-2.60394	1.355096	-0.62517
H	5.19639	2.115069	0.698509	H	5.173511	2.24598	0.085121
H	3.871206	4.037204	0.999508	H	3.824269	4.170467	-0.03893
H	-2.036	-0.56507	-2.45236	H	-2.1139	-1.27062	-2.17093
H	5.742467	0.616108	-1.30351	H	3.482842	-1.36049	-1.66491
H	7.194775	-1.37101	-1.61802	H	4.925268	-3.34694	-1.49507
H	4.580743	-3.66398	0.90373	H	6.901207	-1.64301	1.91703
H	3.158011	-1.68536	1.245737	H	5.424412	0.345405	1.769711
H	6.467128	-4.47637	-0.13305	H	6.62979	-4.34715	-0.31725
H	-5.87359	-1.74817	-2.95176	H	-5.99896	-2.44036	-2.2072
H	-6.64713	-1.05966	-1.49024	H	-6.71085	-1.37868	-0.95323
H	-5.586	-0.0492	-2.47339	H	-5.68961	-0.67849	-2.20999
Free Energy = -1476.75459 Hartree				Free Energy = -1476.754063 Hartree			
**Conf. C**	**X axis(Å)**	**Y axis(Å)**	**Z axis(Å)**	**Conf. D**	**X axis(Å)**	**Y axis(Å)**	**Z axis(Å)**
C	2.647918	-3.18531	-3.29227	C	2.766081	-3.36568	-3.03651
C	2.750692	-3.08257	4.134105	C	2.631222	-2.78679	4.377719
C	2.724107	-1.87765	3.238045	C	2.6252	-1.64289	3.404781
C	3.338203	-2.21067	-2.33135	C	3.426405	-2.33481	-2.1139
C	3.790856	-0.92832	-3.04407	C	3.909307	-1.09979	-2.88796
C	4.466609	0.046477	-2.07389	C	4.553327	-0.06757	-1.95636
C	3.55946	0.410676	-0.86653	C	3.603283	0.372211	-0.80906
C	3.086477	-0.87481	-0.14617	C	3.101365	-0.86482	-0.02589
C	2.430956	-1.85006	-1.14257	C	2.479534	-1.90089	-0.9817
C	-0.27605	-0.88306	0.693897	C	-0.29507	-0.83769	0.735348
C	0.801794	-0.08767	0.819701	C	0.78059	-0.03271	0.80867
C	2.198354	-0.61333	1.127095	C	2.168958	-0.52553	1.196946
O	-1.54429	-0.44145	0.448137	O	-1.55676	-0.42326	0.418586
C	-1.78496	0.882834	0.295923	C	-1.79321	0.880663	0.138852
C	-0.76315	1.816313	0.405495	C	-0.77525	1.823127	0.191032
C	0.611395	1.379754	0.720979	C	0.591743	1.419493	0.574921
C	-3.15443	1.215533	0.007183	C	-3.16148	1.188381	-0.18139
C	-3.40293	2.549991	-0.17292	C	-3.41433	2.507619	-0.44695

N	-2.42215	3.485283	-0.093	N	-2.43952	3.451985	-0.41199
C	-1.0563	3.239715	0.190949	C	-1.07076	3.226973	-0.12516
O	-0.28491	4.183723	0.222528	O	-0.29881	4.16988	-0.17195
O	1.548554	2.154817	0.90131	O	1.52306	2.210432	0.71148
C	2.177224	-1.83564	2.020307	C	2.126601	-1.68745	2.166617
C	-4.24298	0.214203	-0.11914	C	-4.25677	0.186327	-0.19204
C	-5.13341	0.272227	-1.20572	C	-5.4685	0.460591	0.466406
C	-6.19792	-0.61478	-1.3249	C	-6.53456	-0.43174	0.434054
C	-6.39027	-1.60085	-0.35235	C	-6.40555	-1.64043	-0.25715
C	-5.51042	-1.68289	0.732714	C	-5.20529	-1.93775	-0.91236
C	-4.4537	-0.78366	0.845563	C	-4.14805	-1.0325	-0.87973
O	-7.44505	-2.45242	-0.51685	O	-7.47737	-2.48648	-0.25362
C	4.383	1.332892	0.043257	C	4.393366	1.351385	0.070163
C	4.397751	2.80906	-0.2983	C	4.427137	2.801865	-0.36677
O	5.033739	0.881738	0.970819	O	5.004431	0.96214	1.051277
H	3.993217	-1.3473	0.252335	H	3.992461	-1.31109	0.433444
H	2.698427	0.97438	-1.24886	H	2.758685	0.910953	-1.2577
H	2.356086	-4.11091	-2.78122	H	2.451841	-4.2569	-2.47961
H	1.739523	-2.74017	-3.71902	H	1.87566	-2.9454	-3.52239
H	3.307505	-3.45665	-4.12538	H	3.453288	-3.69026	-3.82716
H	2.251886	-2.87805	5.091319	H	2.165901	-3.68315	3.952893
H	2.256288	-3.94369	3.67083	H	3.655799	-3.04556	4.677267
H	3.782079	-3.37301	4.375594	H	2.092393	-2.52805	5.299414
H	3.202404	-0.97775	3.62773	H	3.076038	-0.71257	3.753701
H	4.237523	-2.70498	-1.9307	H	4.309105	-2.80572	-1.65277
H	4.479721	-1.17322	-3.8639	H	4.625875	-1.39637	-3.66592
H	2.915845	-0.44431	-3.50491	H	3.053231	-0.6433	-3.40853
H	4.763013	0.958928	-2.60598	H	4.871904	0.809329	-2.53349
H	5.390946	-0.40619	-1.68863	H	5.461801	-0.49618	-1.51053
H	2.139193	-2.77334	-0.62374	H	2.167792	-2.78991	-0.41668
H	1.502426	-1.40915	-1.53497	H	1.565531	-1.48589	-1.43227
H	-0.27617	-1.96167	0.784006	H	-0.2987	-1.90199	0.931876
H	2.693246	0.186581	1.683601	H	2.64069	0.313167	1.715244
H	-4.40212	2.918631	-0.37733	H	-4.40832	2.852978	-0.70935
H	-2.63702	4.466724	-0.22689	H	-2.65224	4.417344	-0.63593
H	1.716851	-2.74498	1.629388	H	1.694866	-2.62736	1.816944
H	-4.97699	1.015179	-1.98353	H	-5.56978	1.382963	1.032312
H	-6.87789	-0.56745	-2.16945	H	-7.46477	-0.2161	0.949883
H	-5.65718	-2.44552	1.495623	H	-5.09973	-2.87507	-1.45587
H	-3.79134	-0.85461	1.701851	H	-3.23033	-1.27525	-1.40497
H	-7.45907	-3.08624	0.217202	H	-7.25416	-3.28477	-0.75728
H	4.516132	2.975127	-1.37557	H	5.212516	3.333648	0.175141
H	3.433455	3.237809	-0.00259	H	4.587588	2.89735	-1.44717

H	5.202979	3.307156	0.246467	H	3.453471	3.249874	-0.13818
Free Energy = -1476.753921 Hartree				Free Energy = -1476.753558 Hartree			
**Conf. E**	**X axis(Å)**	**Y axis(Å)**	**Z axis(Å)**	**Conf. F**	**X axis(Å)**	**Y axis(Å)**	**Z axis(Å)**
C	2.746084	-3.12777	-3.0406	C	-2.62523	-2.97835	3.24458
C	2.548557	-3.73991	3.437973	C	-2.63525	-3.93779	-3.18952
C	2.685238	-2.7309	2.331739	C	-2.74164	-2.86821	-2.13834
C	3.415835	-2.15074	-2.06753	C	-3.32715	-2.05256	2.244473
C	3.950637	-0.904	-2.7872	C	-3.83769	-0.76874	2.91446
C	4.597078	0.081144	-1.80723	C	-4.51827	0.16351	1.90596
C	3.636173	0.501539	-0.66141	C	-3.59871	0.521351	0.705927
C	3.09616	-0.75271	0.068449	C	-3.08173	-0.77106	0.027966
C	2.457654	-1.7265	-0.94192	C	-2.40689	-1.68821	1.067568
C	-0.28159	-0.76584	0.795891	C	0.266199	-0.80803	-0.78758
C	0.775304	0.061812	0.889216	C	-0.79627	0.007974	-0.91321
C	2.160755	-0.43353	1.282648	C	-2.18957	-0.51734	-1.2328
O	-1.54613	-0.37501	0.466711	O	1.536809	-0.39112	-0.51876
C	-1.80486	0.928934	0.201812	C	1.794482	0.928367	-0.34453
C	-0.80772	1.89213	0.281218	C	0.789744	1.880016	-0.4604
C	0.565843	1.51139	0.668623	C	-0.58861	1.469894	-0.79802
C	-3.17473	1.211842	-0.13329	C	3.163392	1.235116	-0.02633
C	-3.45379	2.529728	-0.37885	C	3.430873	2.563397	0.171998
N	-2.50137	3.494791	-0.31208	N	2.468013	3.516085	0.082175
C	-1.13163	3.295048	-0.01087	C	1.103678	3.296388	-0.22896
O	-0.38147	4.256227	-0.02613	O	0.349395	4.253717	-0.26806
O	1.483122	2.316427	0.81686	O	-1.51192	2.260263	-0.9829
C	2.053217	-1.55398	2.302611	C	-2.10725	-1.69335	-2.19074
C	-4.2459	0.184997	-0.18138	C	4.231898	0.214292	0.115597
C	-5.47376	0.41643	0.463339	C	5.097628	0.248774	1.222984
C	-6.51744	-0.50009	0.395136	C	6.143626	-0.6574	1.35997
C	-6.34904	-1.69024	-0.31943	C	6.341526	-1.63954	0.384544
C	-5.1321	-1.94487	-0.96172	C	5.486101	-1.69808	-0.72137
C	-4.09755	-1.01581	-0.89298	C	4.44791	-0.77978	-0.85175
O	-7.40032	-2.56102	-0.35096	O	7.377126	-2.51063	0.567101
C	4.432129	1.431909	0.263281	C	-4.42954	1.398616	-0.23986
C	4.479083	2.900373	-0.10579	C	-4.46543	2.885548	0.04711
O	5.035101	0.991459	1.227688	O	-5.06731	0.904325	-1.1545
H	3.978709	-1.23223	0.509113	H	-3.97799	-1.27408	-0.355
H	2.809895	1.072932	-1.10294	H	-2.75874	1.115966	1.087082
H	2.394127	-4.02855	-2.52316	H	-2.2946	-3.90753	2.764709
H	1.878856	-2.66401	-3.52888	H	-1.73941	-2.49315	3.675073
H	3.440461	-3.44362	-3.82852	H	-3.29161	-3.24771	4.072945
H	3.516297	-3.92895	3.92196	H	-3.61571	-4.1517	-3.63612
H	1.847102	-3.40169	4.208349	H	-1.95477	-3.64459	-3.99639

H	2.195615	-4.70799	3.056698	H	-2.2728	-4.8838	-2.76427
H	3.367721	-3.00486	1.528698	H	-3.4042	-3.09455	-1.30426
H	4.274844	-2.66452	-1.60661	H	-4.20081	-2.58796	1.838977
H	4.678941	-1.19301	-3.55708	H	-4.5392	-1.01524	3.723009
H	3.11971	-0.40877	-3.31295	H	-2.98947	-0.2476	3.384639
H	4.944335	0.970937	-2.34702	H	-4.84777	1.08127	2.40883
H	5.488221	-0.38125	-1.3601	H	-5.42356	-0.32162	1.515024
H	2.087812	-2.61713	-0.41857	H	-2.05182	-2.60527	0.58105
H	1.576428	-1.25229	-1.39877	H	-1.51293	-1.18909	1.469406
H	-0.25126	-1.83279	0.980827	H	0.235938	-1.88572	-0.89325
H	2.621399	0.411259	1.809246	H	-2.67318	0.293571	-1.79113
H	-4.45149	2.85727	-0.64989	H	4.431892	2.912697	0.400047
H	-2.73293	4.459454	-0.51985	H	2.696986	4.492529	0.228754
H	1.385169	-1.3194	3.134573	H	-1.46078	-1.50662	-3.05144
H	-5.60594	1.323883	1.046746	H	4.935601	0.988357	2.002832
H	-7.46044	-0.31761	0.900431	H	6.804243	-0.62832	2.220556
H	-4.99553	-2.86723	-1.52353	H	5.637393	-2.45759	-1.48649
H	-3.16634	-1.22562	-1.40862	H	3.804524	-0.8331	-1.72361
H	-7.15057	-3.34288	-0.86778	H	7.396806	-3.13996	-0.17066
H	5.258662	3.403379	0.470762	H	-3.49927	3.313749	-0.2434
H	4.65616	3.042151	-1.17859	H	-5.26543	3.355631	-0.52927
H	3.503425	3.34252	0.126008	H	-4.60536	3.08754	1.11573
Free Energy = -1476.752583 Hartree				Free Energy = -1476.752499 Hartree			
**Conf. G**	**X axis(Å)**	**Y axis(Å)**	**Z axis(Å)**	**Conf. H**	**X axis(Å)**	**Y axis(Å)**	**Z axis(Å)**
C	7.008978	0.869044	0.065203	C	7.006361	0.880037	0.076648
C	2.860692	-3.46335	3.306608	C	2.856762	-3.46863	3.301748
C	2.725334	-2.05405	2.804706	C	2.721668	-2.05884	2.801126
C	5.622403	0.676447	-0.55948	C	5.622285	0.682855	-0.55212
C	5.643335	-0.36233	-1.69066	C	5.649719	-0.35675	-1.68239
C	4.247669	-0.56984	-2.28992	C	4.256492	-0.56898	-2.28563
C	3.225675	-1.00134	-1.21133	C	3.232691	-1.00276	-1.20984
C	3.16383	0.059208	-0.07216	C	3.164028	0.058457	-0.07173
C	4.578209	0.286257	0.499694	C	4.575948	0.290556	0.50419
C	-0.27627	-0.72595	0.659986	C	-0.27769	-0.72835	0.650327
C	0.743363	0.13994	0.796986	C	0.741722	0.137098	0.792786
C	2.173848	-0.28198	1.104287	C	2.17168	-0.2852	1.10213
O	-1.56751	-0.35989	0.419364	O	-1.56867	-0.36086	0.409667
C	-1.8966	0.94977	0.306804	C	-1.89697	0.94907	0.301908
C	-0.94299	1.948588	0.452349	C	-0.94354	1.947328	0.451714
C	0.465339	1.5962	0.733504	C	0.464243	1.593612	0.734007
C	-3.29098	1.194892	0.048861	C	-3.29129	1.195476	0.044655
C	-3.64189	2.515014	-0.04368	C	-3.64206	2.515586	-0.04515
N	-2.73505	3.51525	0.100291	N	-2.73504	3.515392	0.101955

C	-1.34706	3.356233	0.336327	C	-1.34739	3.355352	0.339833
O	-0.64846	4.352712	0.413428	O	-0.64902	4.351632	0.421503
O	1.359724	2.420056	0.912095	O	1.358308	2.416788	0.917332
C	2.294633	-1.70887	1.587338	C	2.292023	-1.71265	1.583662
C	-4.31094	0.124593	-0.08516	C	-4.31075	0.124731	-0.09045
C	-4.11636	-0.99205	-0.91358	C	-5.5269	0.219272	0.602007
C	-5.1059	-1.95986	-1.06182	C	-6.52563	-0.74174	0.45145
C	-6.32333	-1.83012	-0.38434	C	-6.3178	-1.83397	-0.39524
C	-6.53745	-0.72617	0.446564	C	-5.10715	-1.95197	-1.08853
C	-5.53787	0.229667	0.593574	C	-4.12288	-0.98388	-0.93661
O	-7.33144	-2.74554	-0.49083	O	-7.24929	-2.81489	-0.58406
C	1.865389	-1.31519	-1.83216	C	1.874791	-1.32177	-1.83349
C	1.239176	-0.2934	-2.76862	C	1.249172	-0.30534	-2.77607
O	1.301814	-2.37531	-1.6067	O	1.312345	-2.38152	-1.60403
H	2.831143	1.012394	-0.49836	H	2.829526	1.010242	-0.49972
H	3.570564	-1.94838	-0.77862	H	3.579318	-1.94817	-0.77493
H	6.988163	1.634488	0.849928	H	6.981005	1.646287	0.860462
H	7.369058	-0.06366	0.518661	H	7.367631	-0.05117	0.532244
H	7.744657	1.179546	-0.68667	H	7.743492	1.191736	-0.67332
H	2.236866	-3.62931	4.195513	H	3.89058	-3.68666	3.602306
H	2.567343	-4.19305	2.544452	H	2.232126	-3.63565	4.189904
H	3.894752	-3.68115	3.606442	H	2.564153	-4.19755	2.538573
H	3.002882	-1.26654	3.508357	H	2.998494	-1.27192	3.505775
H	5.31012	1.638149	-0.99683	H	5.308537	1.643286	-0.99125
H	6.34378	-0.05201	-2.47747	H	6.351633	-0.04489	-2.46728
H	6.018091	-1.31947	-1.29742	H	6.026243	-1.31243	-1.28725
H	4.286442	-1.32704	-3.08434	H	4.300024	-1.32665	-3.07936
H	3.917191	0.36624	-2.76059	H	3.924532	0.365693	-2.75808
H	4.520985	1.07569	1.260169	H	4.513822	1.080608	1.26366
H	4.908996	-0.62715	1.016275	H	4.907844	-0.62135	1.022729
H	-0.19855	-1.80328	0.713143	H	-0.20034	-1.80594	0.698436
H	2.478981	0.381947	1.923188	H	2.474713	0.377672	1.922653
H	-4.66248	2.818186	-0.24996	H	-4.66249	2.819176	-0.25173
H	-3.02058	4.483572	0.010575	H	-3.01985	4.484007	0.013418
H	2.023454	-2.49414	0.881613	H	2.021751	-2.49719	0.876847
H	-3.18428	-1.10598	-1.45659	H	-5.69001	1.047359	1.286785
H	-4.93309	-2.81561	-1.7121	H	-7.45834	-0.64847	1.00496
H	-7.47957	-0.64146	0.978665	H	-4.95873	-2.80377	-1.74453
H	-5.70408	1.067134	1.266184	H	-3.1952	-1.08606	-1.48966
H	-7.04873	-3.46092	-1.08167	H	-8.03137	-2.61767	-0.04537
H	1.732261	-0.34334	-3.74804	H	1.360474	0.721084	-2.41136
H	0.18103	-0.52764	-2.90332	H	1.746644	-0.35643	-3.75319
H	1.355411	0.731514	-2.40138	H	0.192438	-0.54374	-2.91442

Free Energy = -1476.752121 Hartree	Free Energy = -1476.751993 Hartree

Figure S1. HPLC chromatogram of acremocholrins A (**1**) and B (**2**) at 225nm.

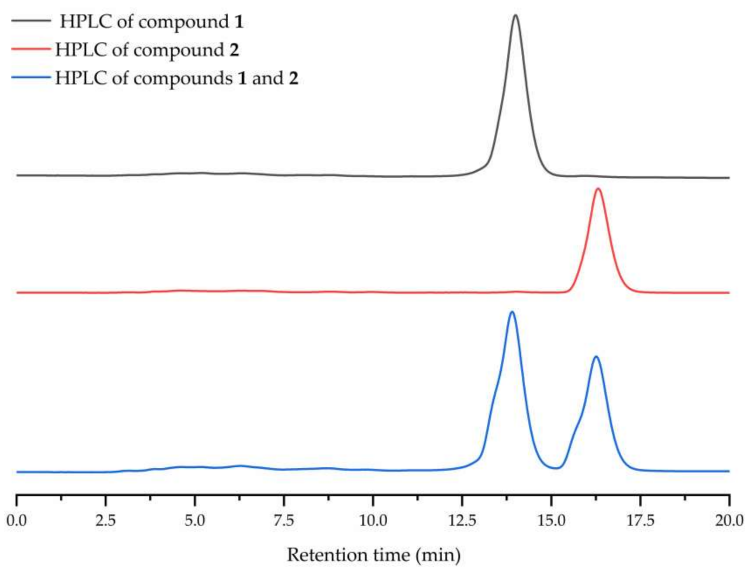

Figure S2. HRESIMS spectrum of acremocholrin A (**1**).

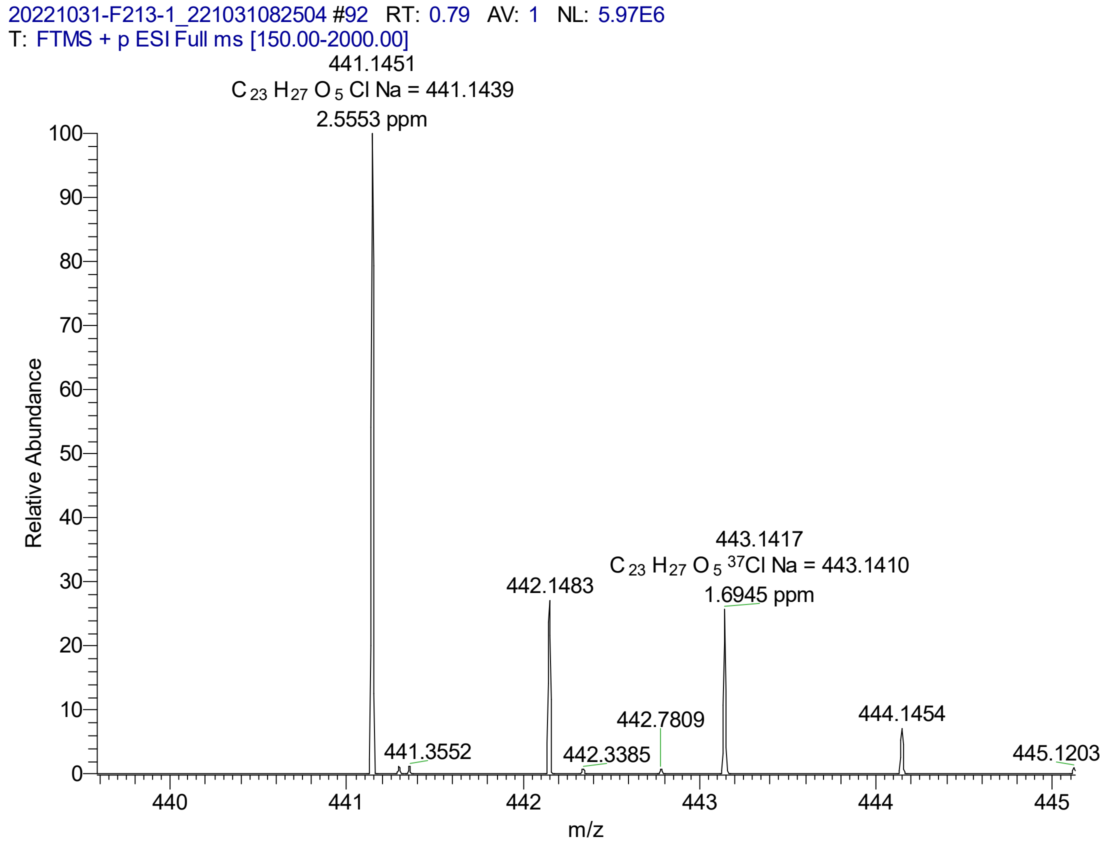

Figure S3. ^{1}H NMR (600 MHz, DMSO-d_6) spectrum of acremocholrin A (**1**).

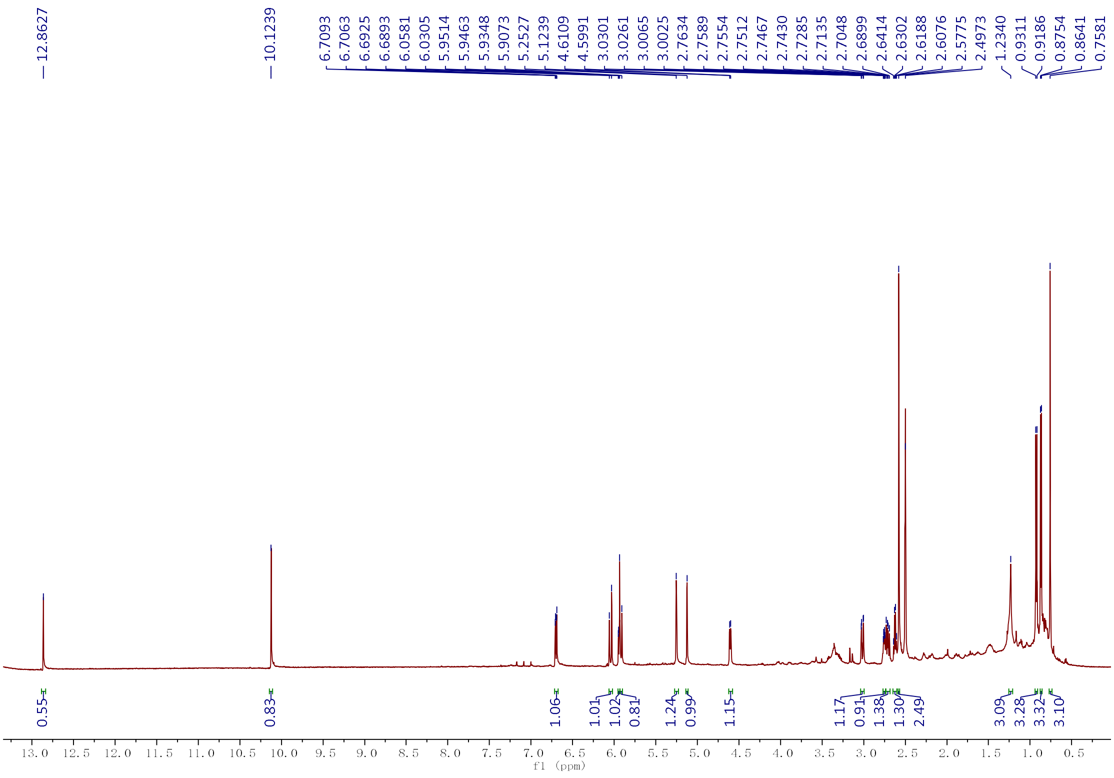

Figure S4. DEPT-Q (150 MHz, DMSO-d_6) spectra of acremocholrin A (**1**).

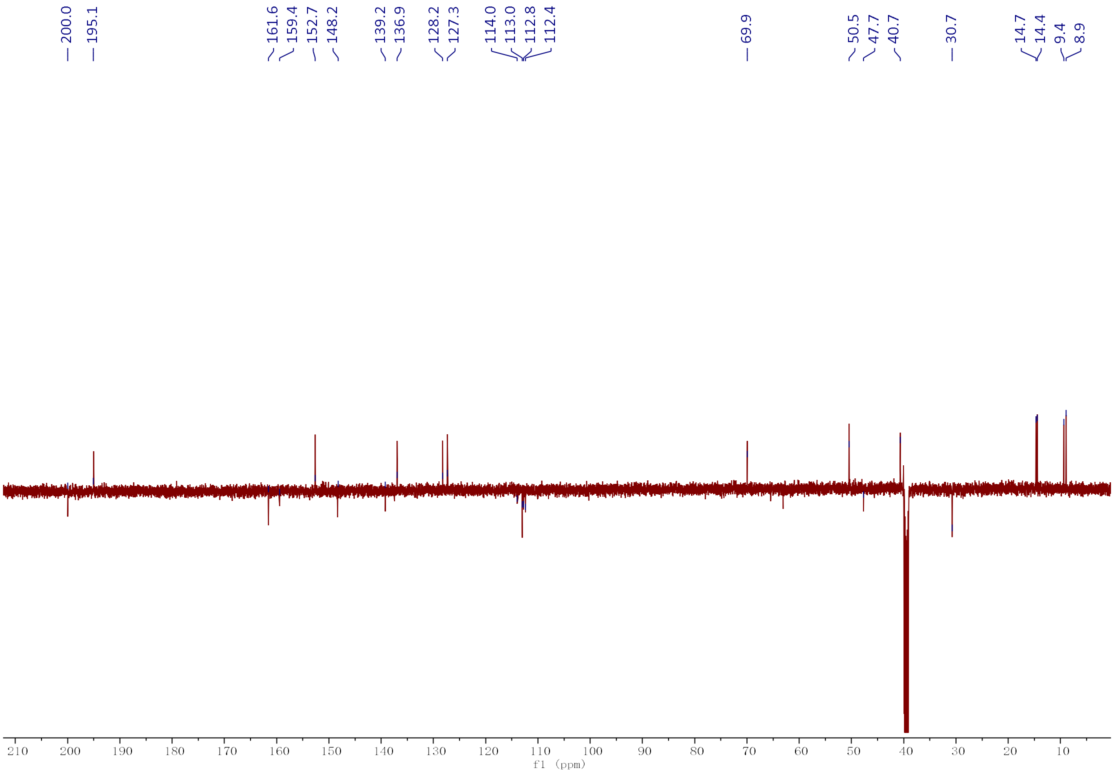

Figure S5. HSQC spectrum of acremocholrin A (**1**).

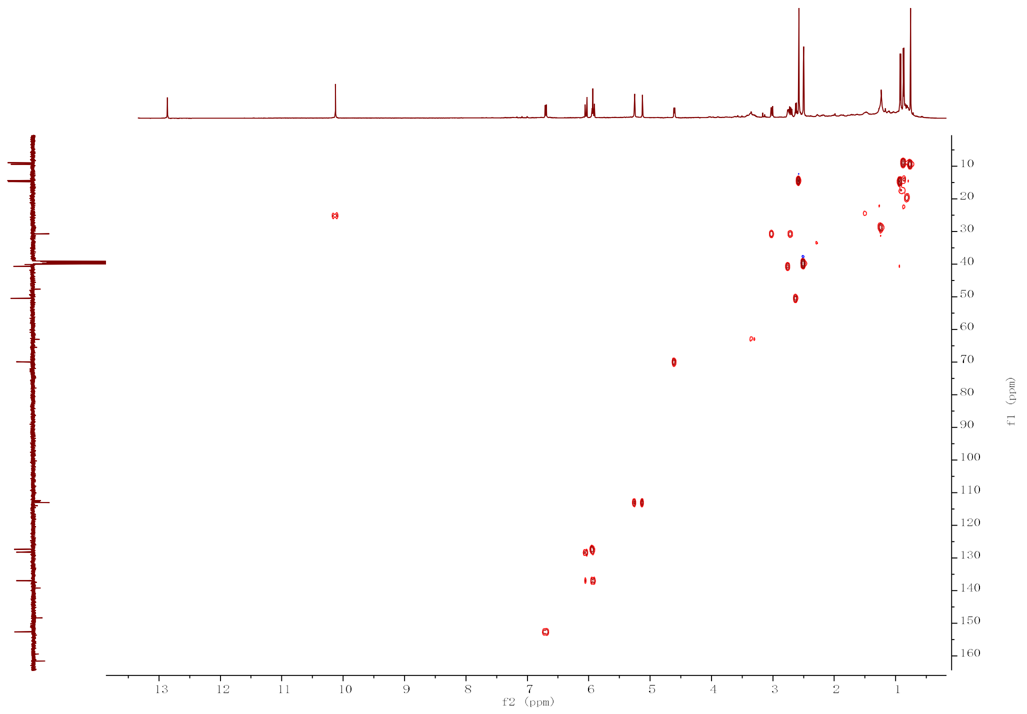

Figure S6. ^1H-^1H COSY spectrum of acremocholrin A (**1**).

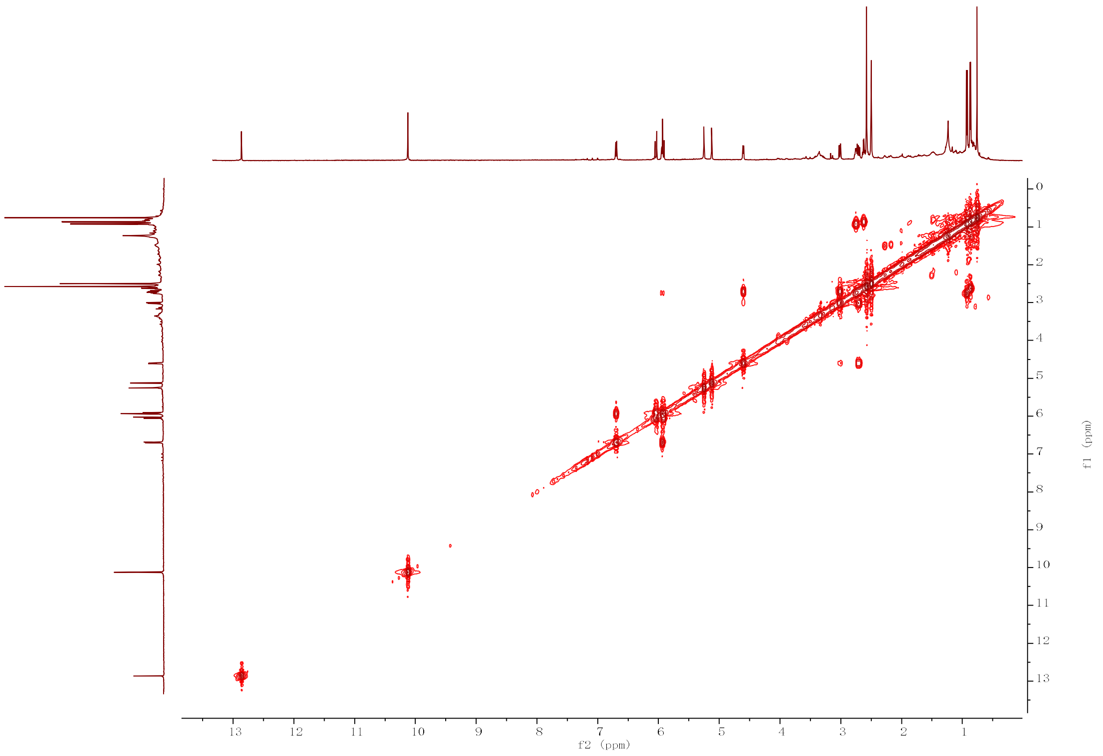

Figure S7. HMBC spectrum of acremocholrin A (**1**).

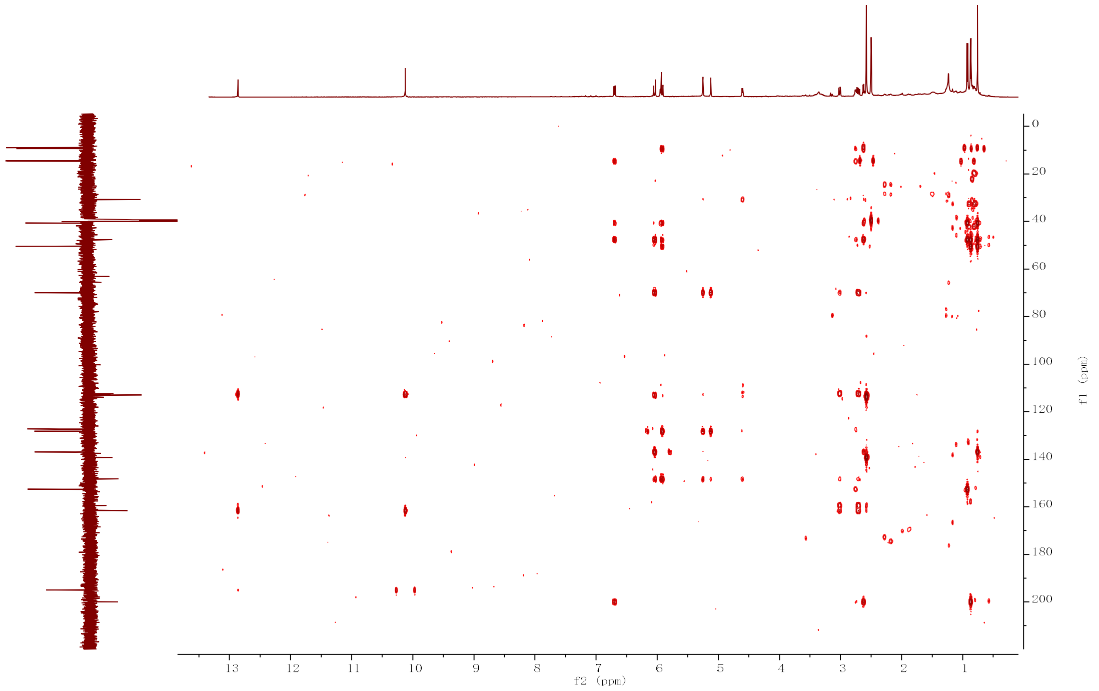

Figure S8. NOE difference spectrum of acremocholrin A (**1**).

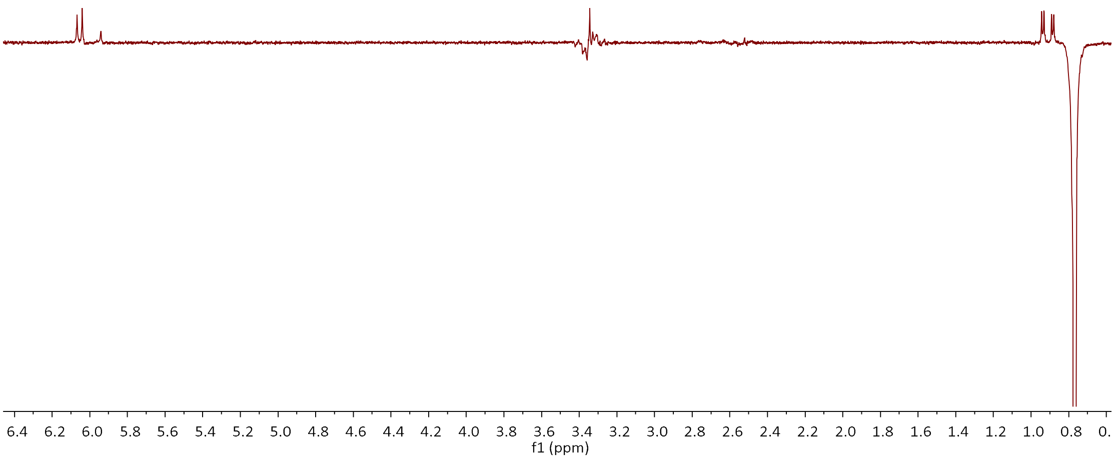

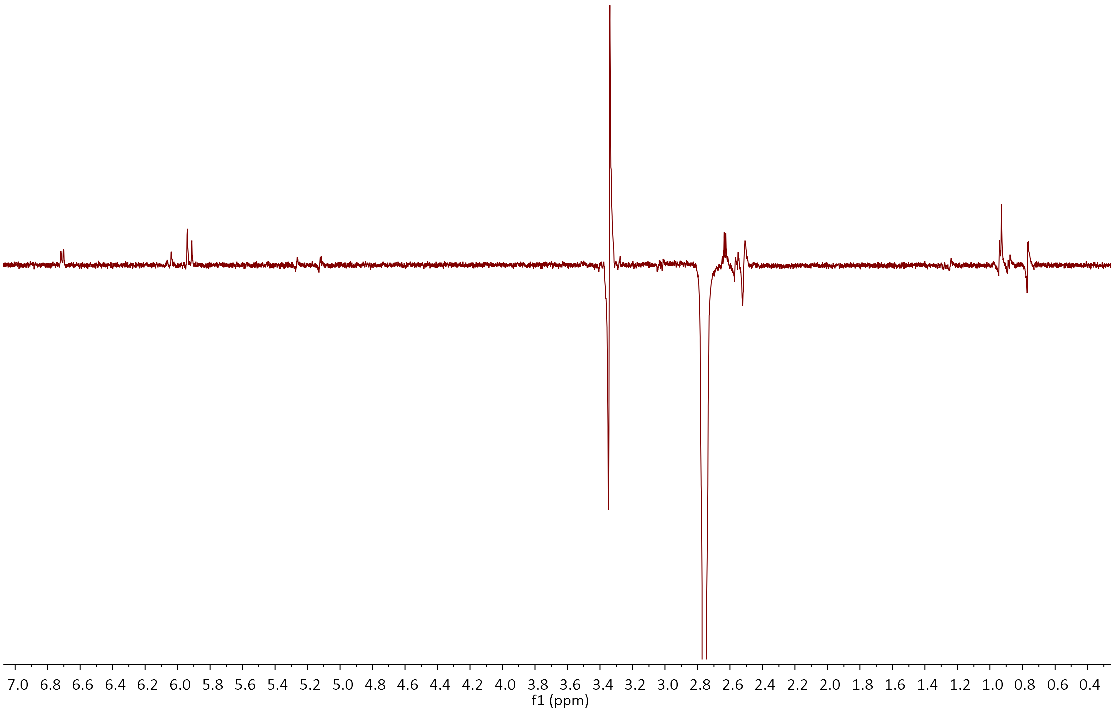

Figure S9. HRESIMS spectrum of acremocholrin B (**2**).

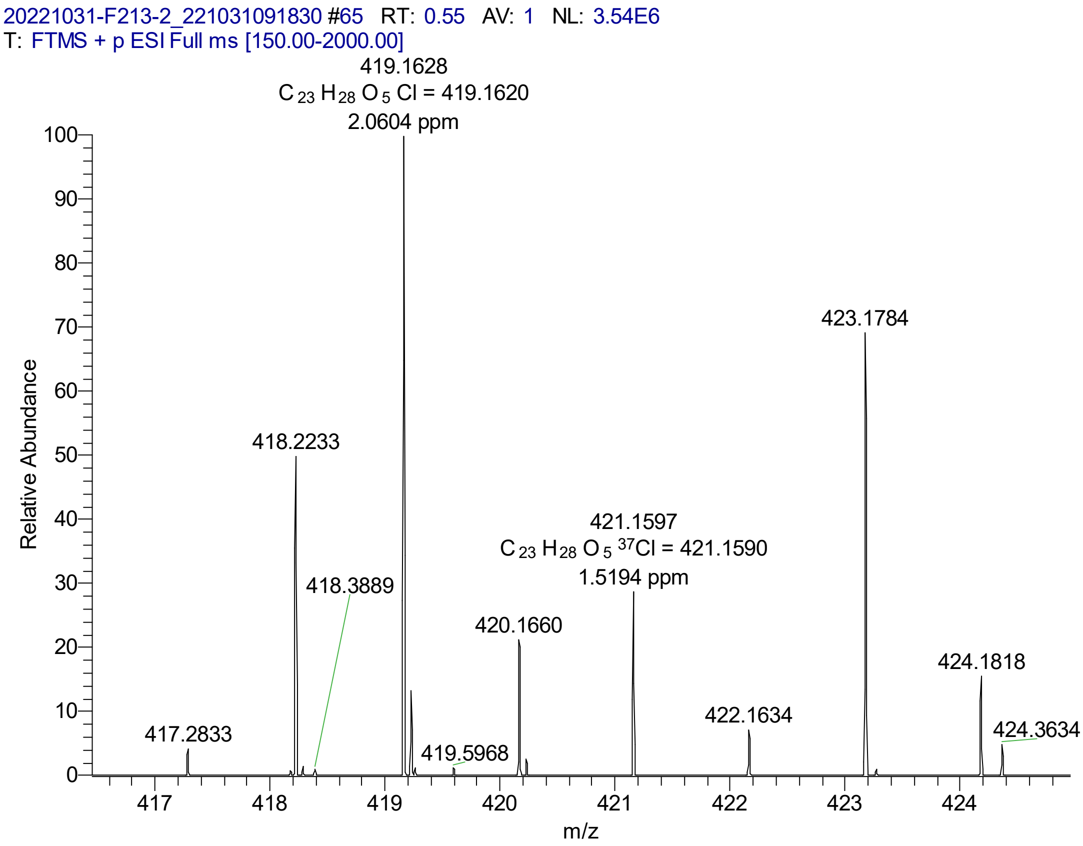

Figure S10. ^{1}H NMR (600 MHz, DMSO-d_6) spectrum of acremocholrin B (**2**).

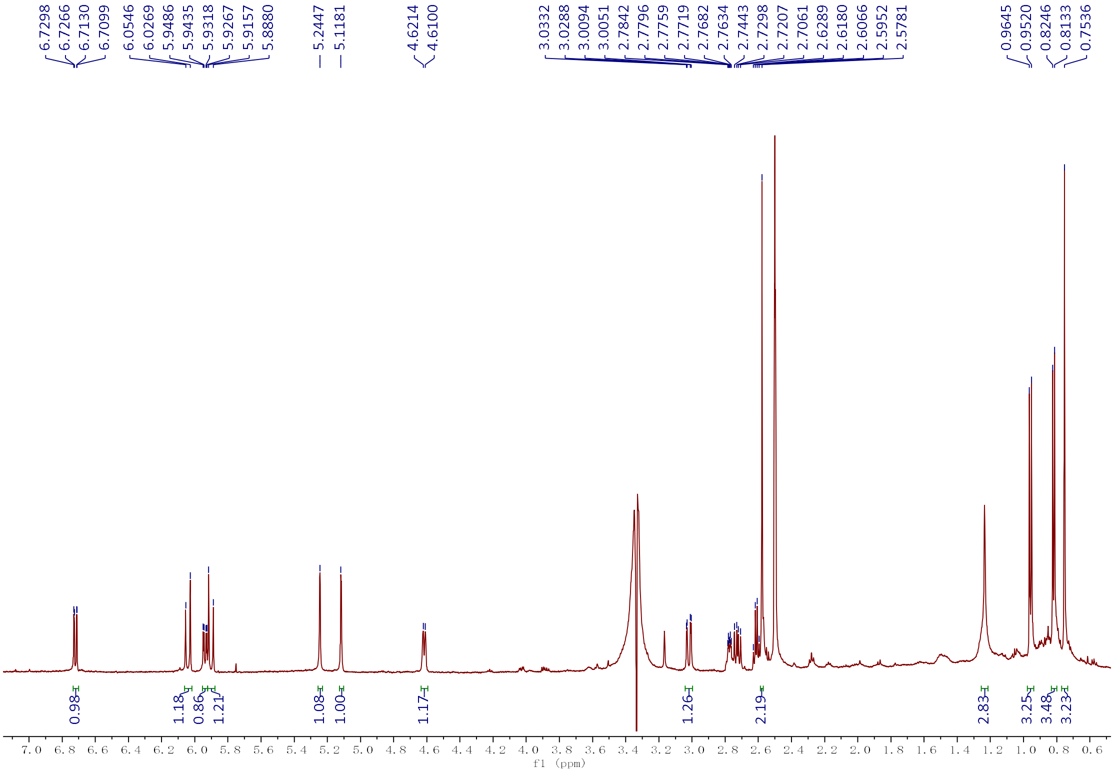

Figure S11. DEPT-Q (150 MHz, DMSO-d_6) spectrum of acremocholrin B (**2**).

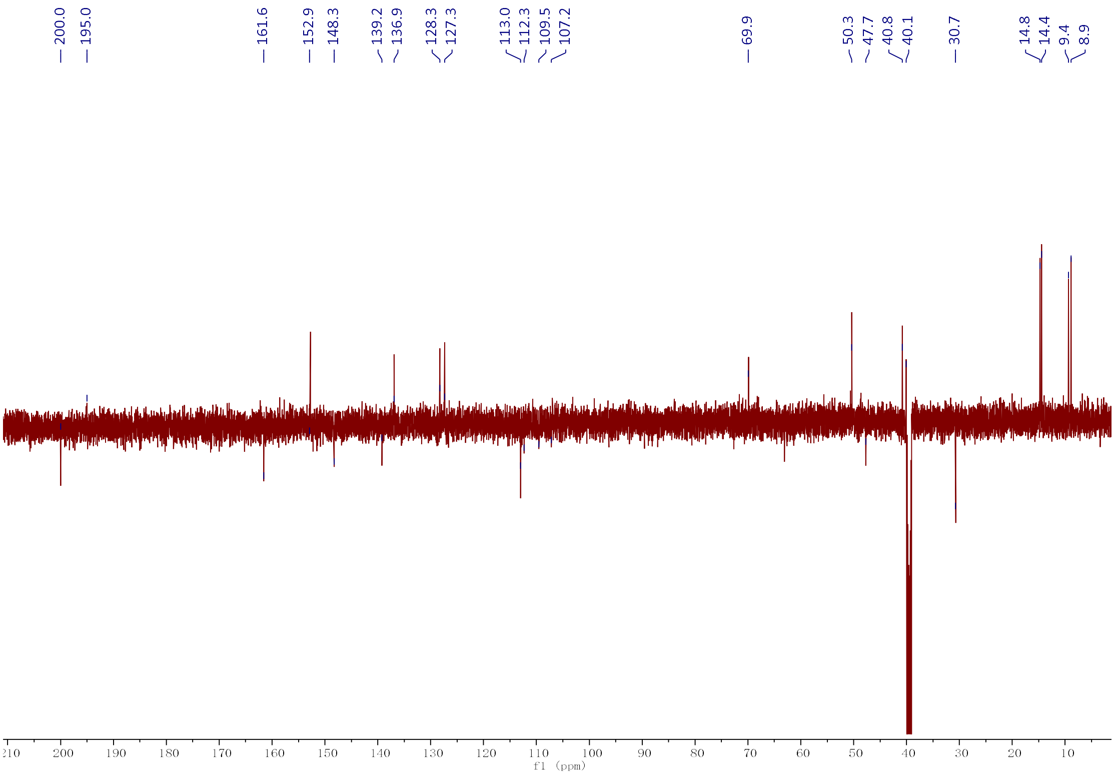

Figure S12. HSQC spectrum of acremocholrin B (**2**).

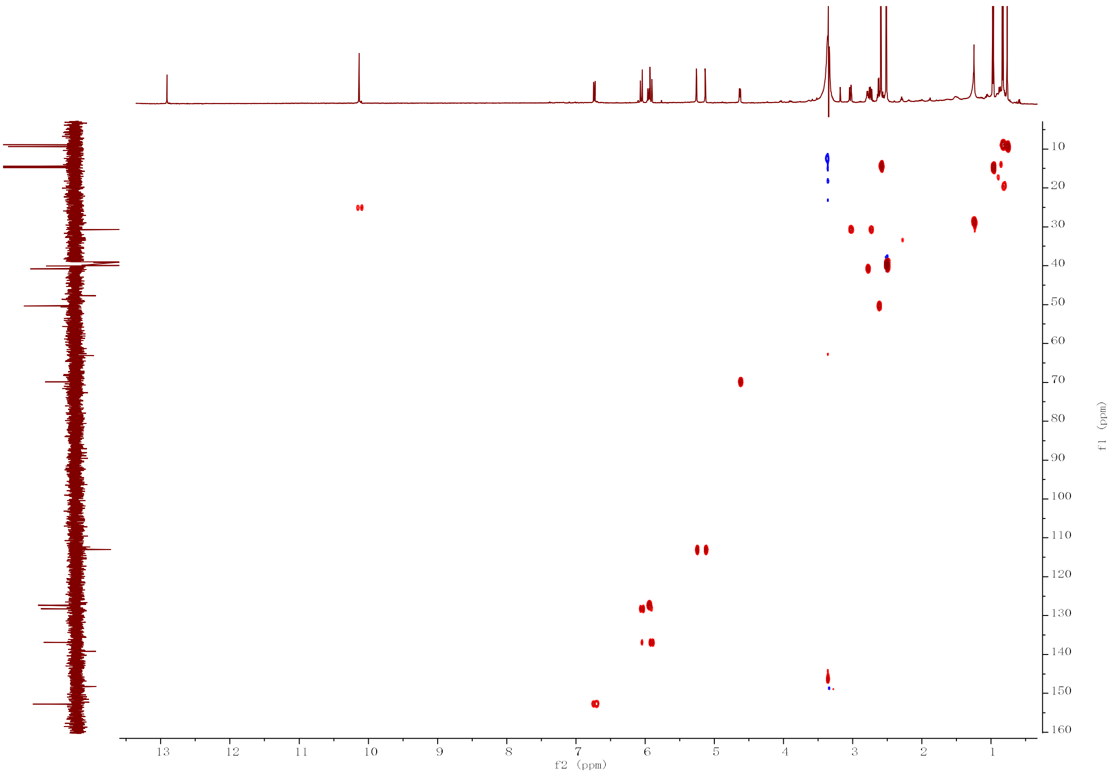

Figure S13. ^{1}H-^{1}H COSY spectrum of acremocholrin B (**2**).

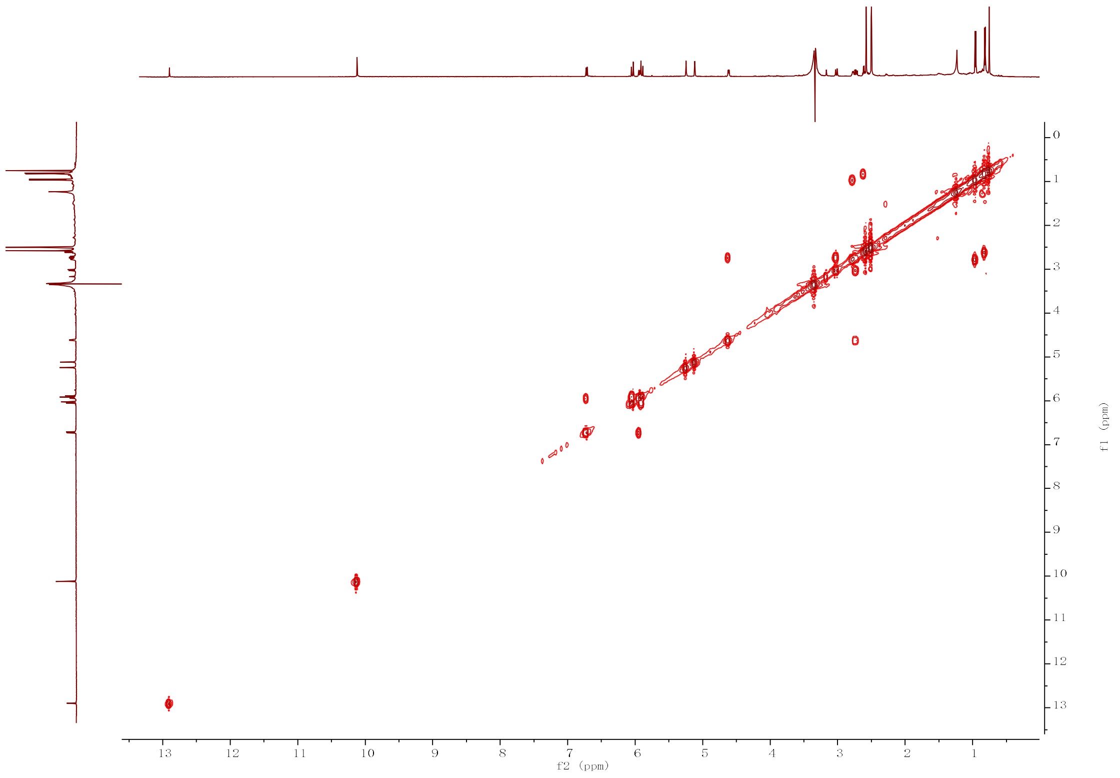

Figure S14. HMBC spectrum of acremocholrin B (**2**).

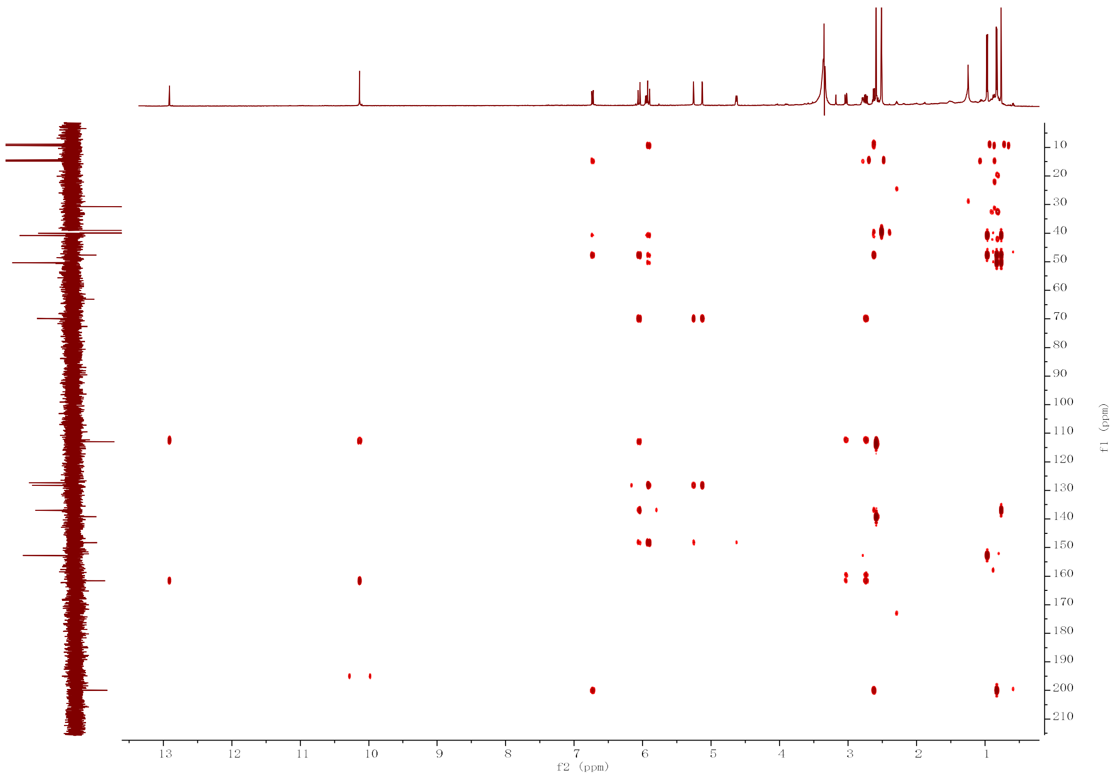

Figure S15. NOE difference spectrum of acremocholrin B (**2**).

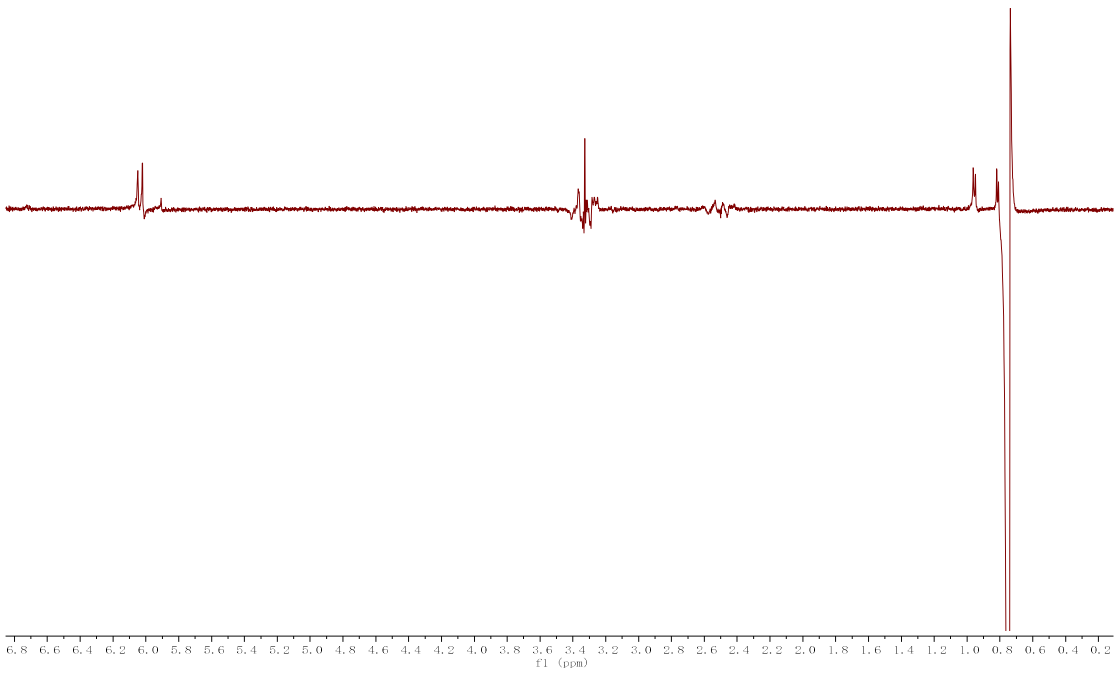

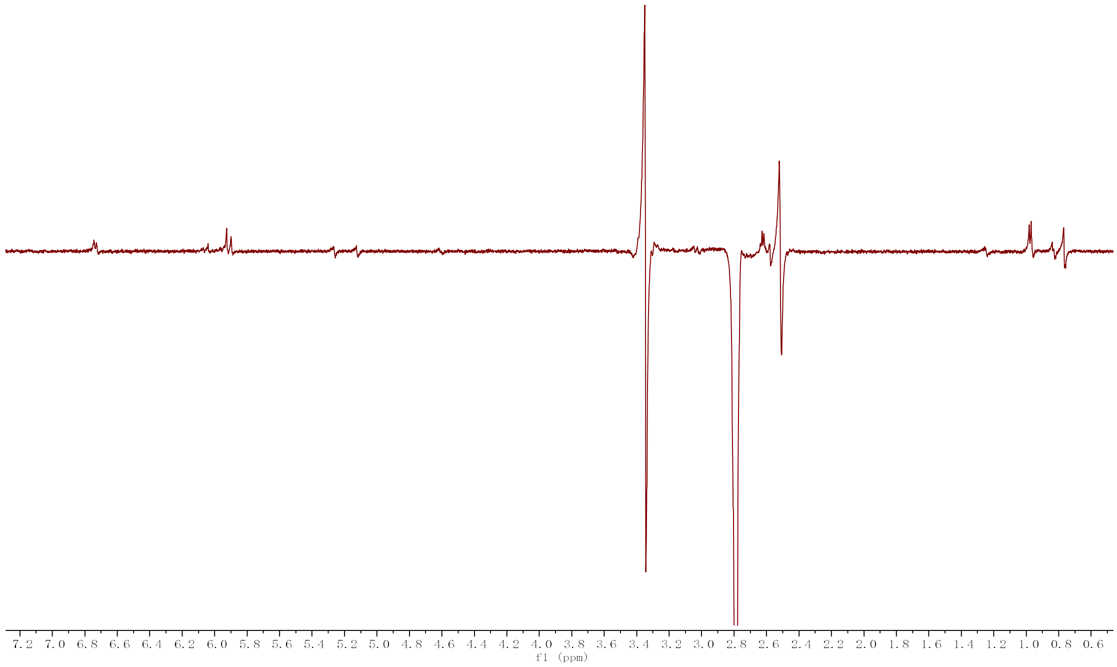

Figure S16. HRESIMS spectrum of Acremopyridone A (**7**).

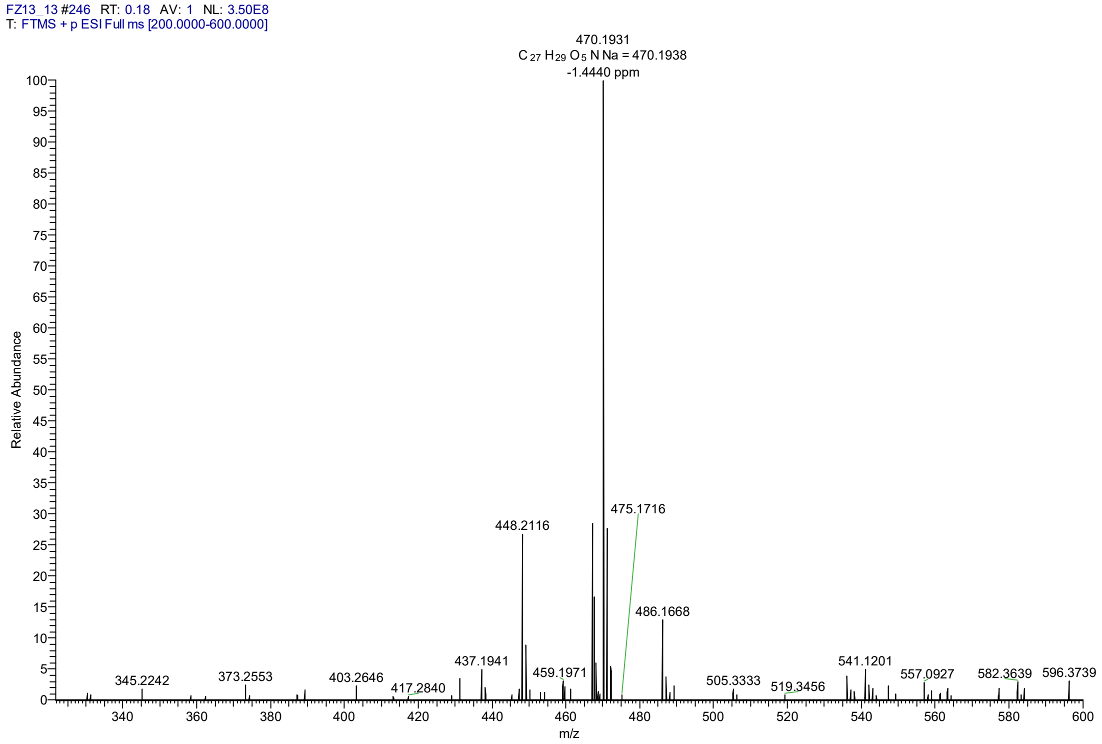

Figure S17. ^{1}H NMR (600 MHz, DMSO-*d*6) spectrum of Acremopyridone A (**7**).

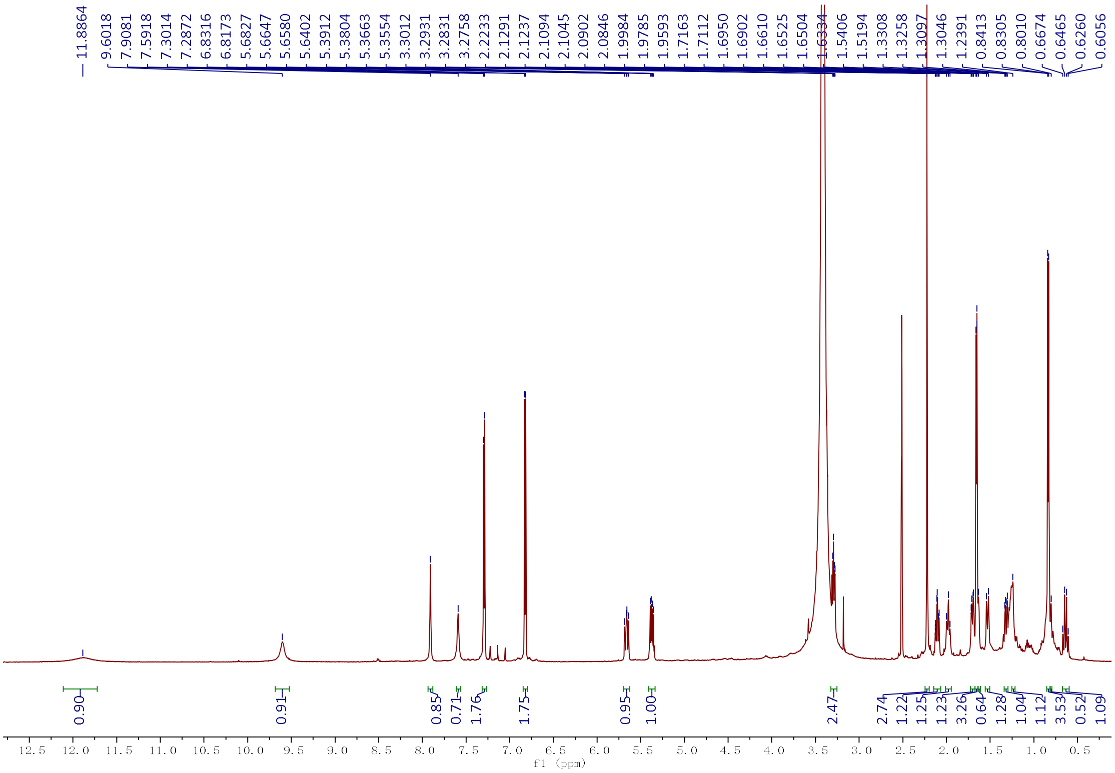

Figure S18. DEPT-Q (150 MHz, DMSO-d_6) spectrum of Acremopyridone A (**7**).

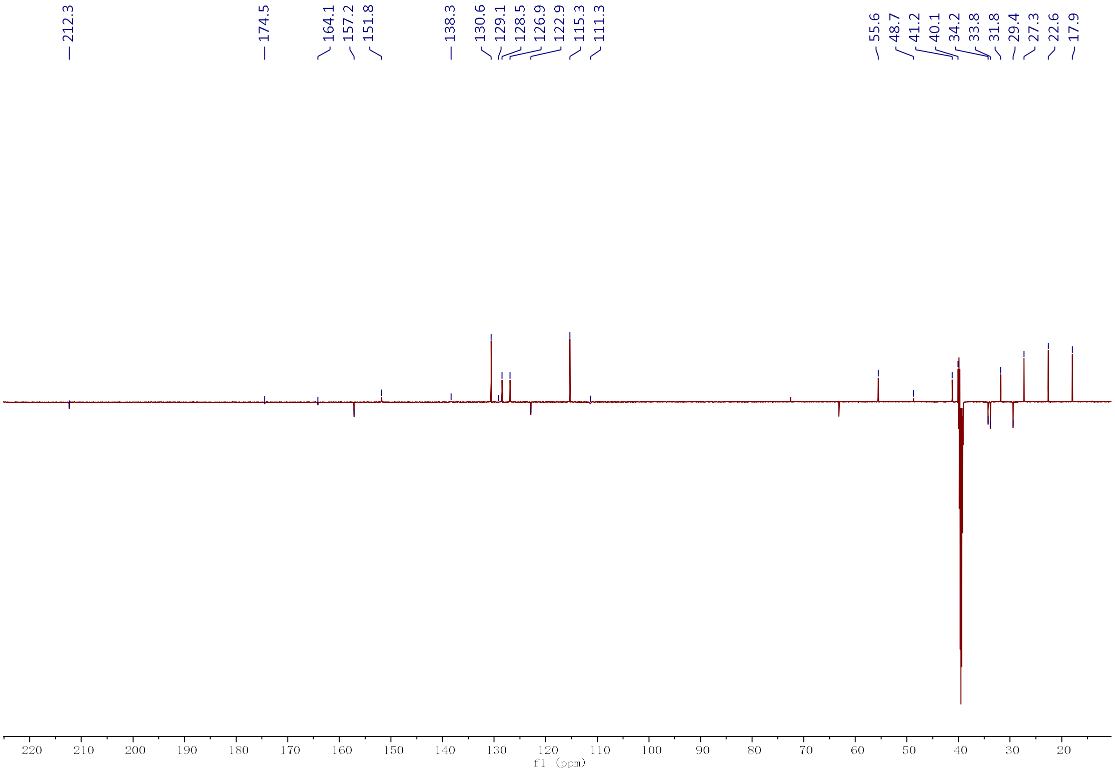

Figure S19. HSQC spectrum of Acremopyridone A (**7**).

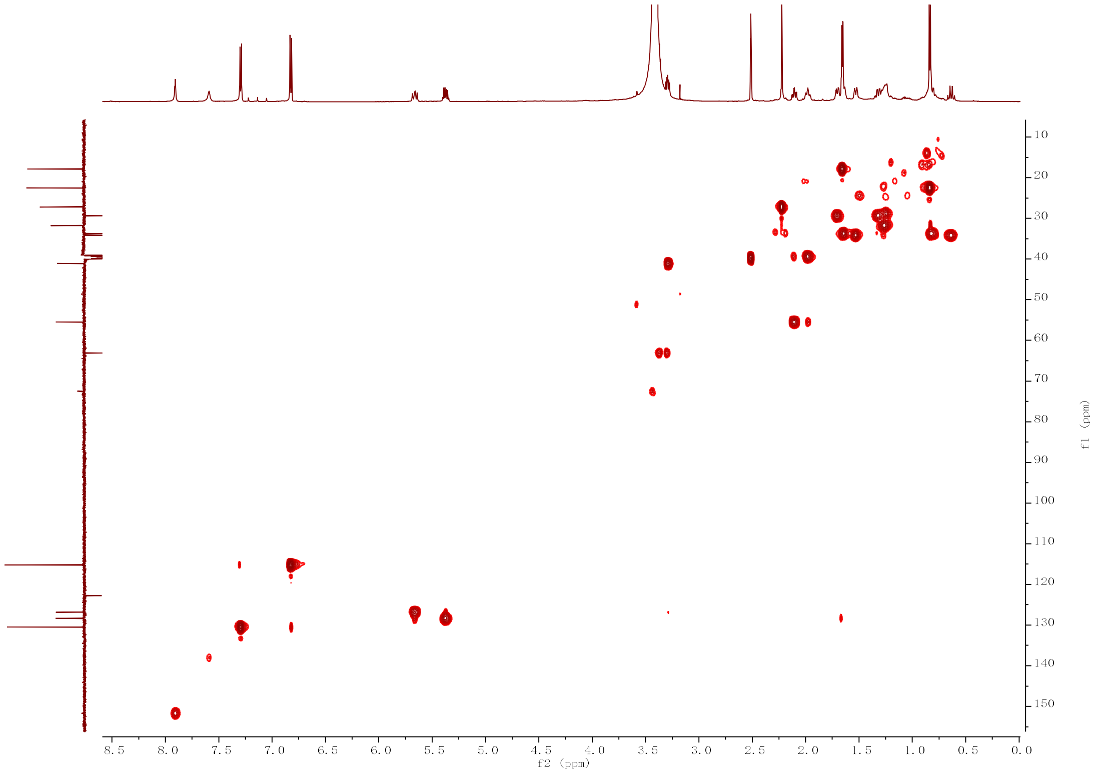

Figure S20. ^{1}H-^{1}H COSY spectrum of Acremopyridone A (**7**).

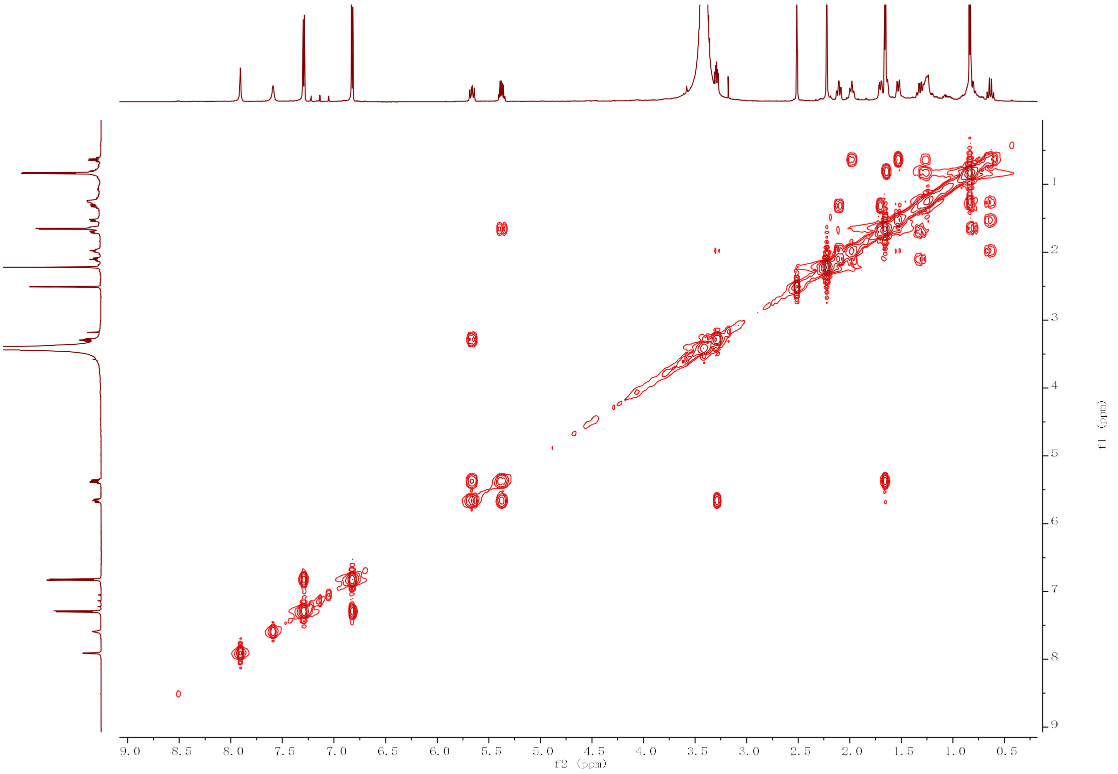

Figure S21. HMBC spectrum of Acremopyridone A (**7**).

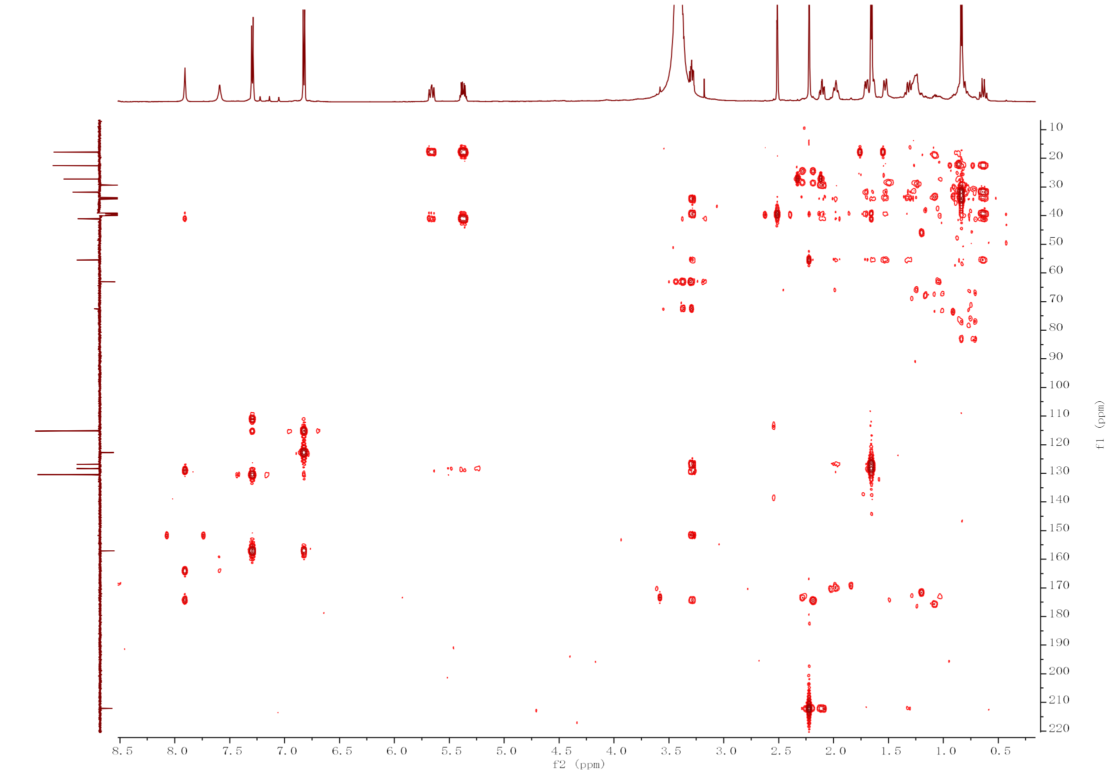

Figure S22. NOESY spectrum of Acremopyridone A (**7**).

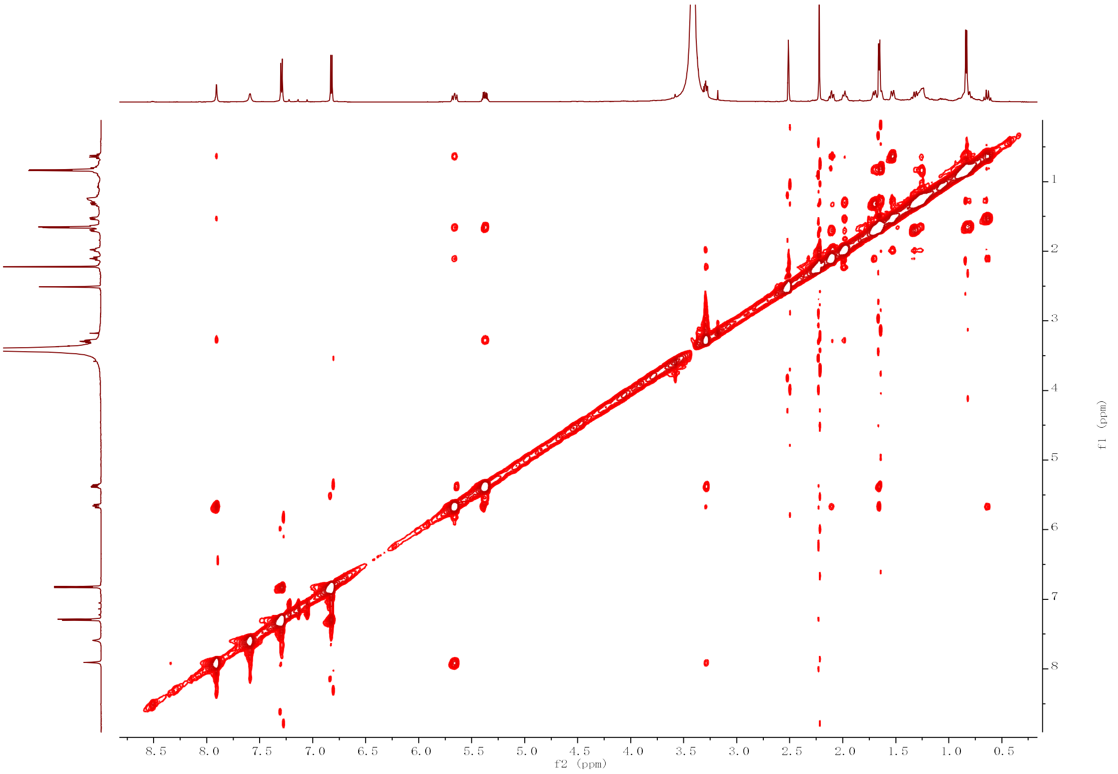

Figure S23. HRESIMS spectrum of acremoketene A (**12**).

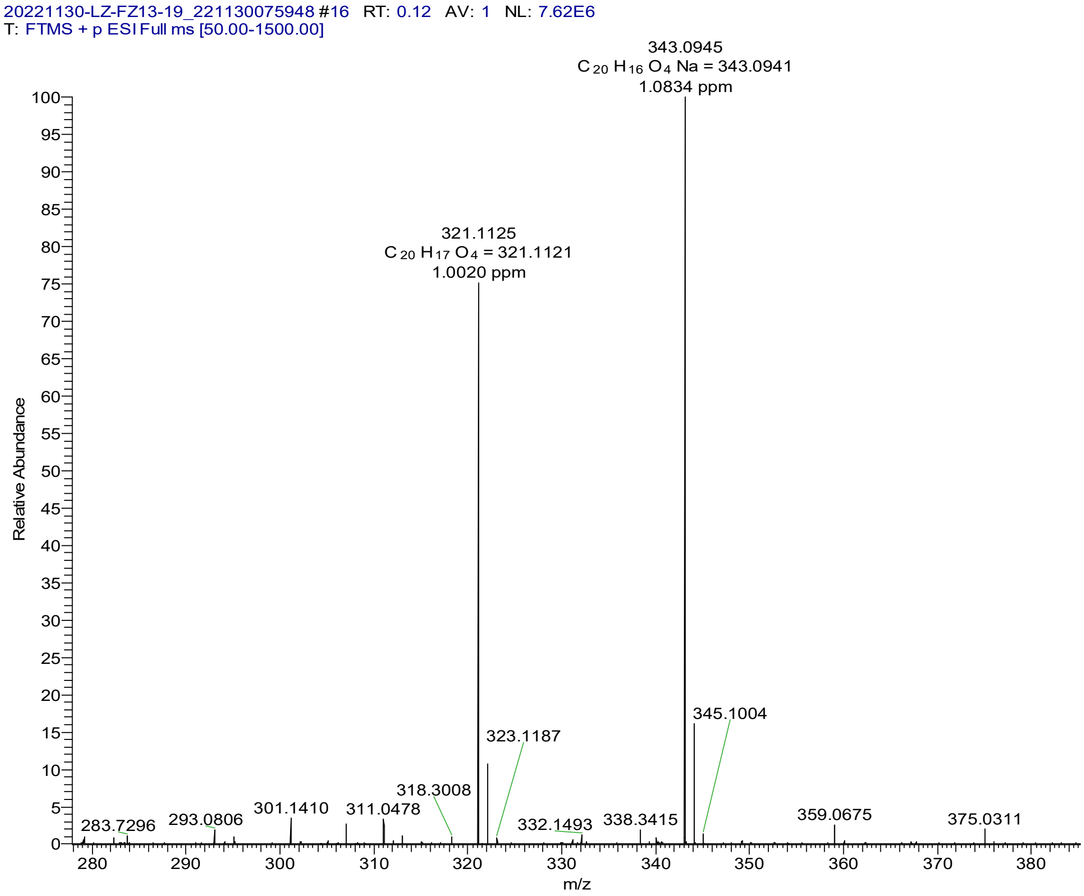

Figure S24. ^{1}H NMR (600 MHz, DMSO-d_6) spectrum of acremoketene A (**12**).

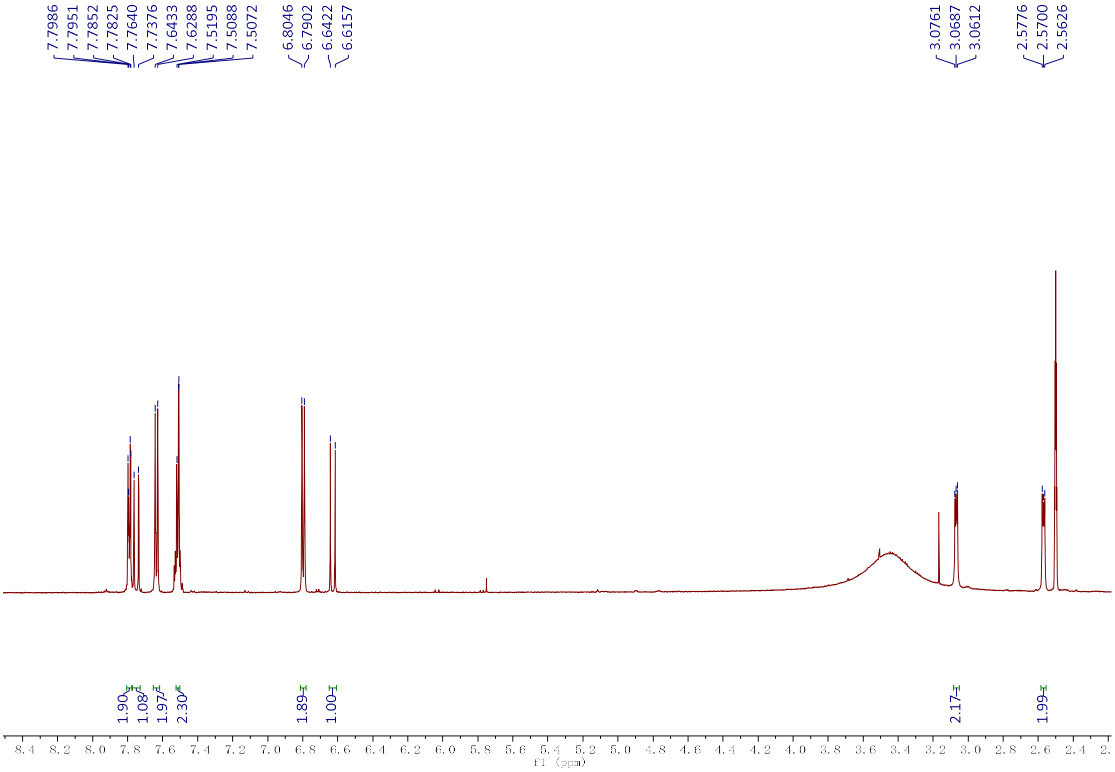

Figure S25. DEPT-Q (150 MHz, DMSO-d_6) spectrum of acremoketene A (**12**).

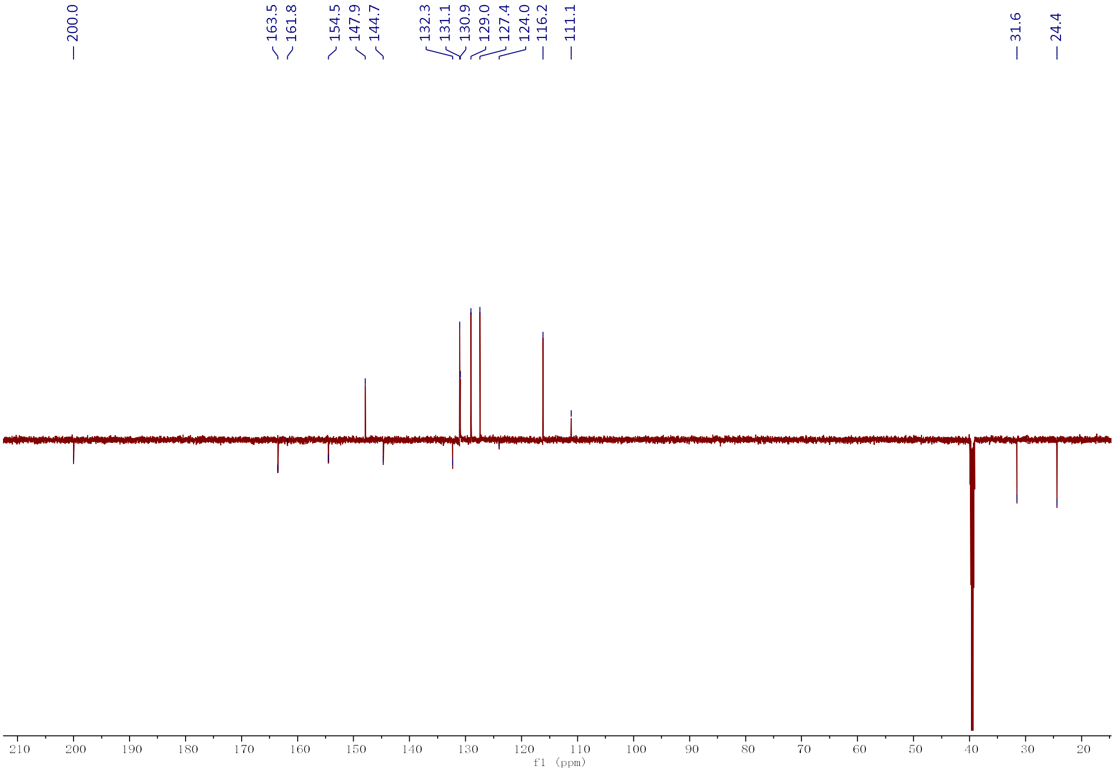

Figure S26. HSQC spectrum of acremoketene A (**12**).

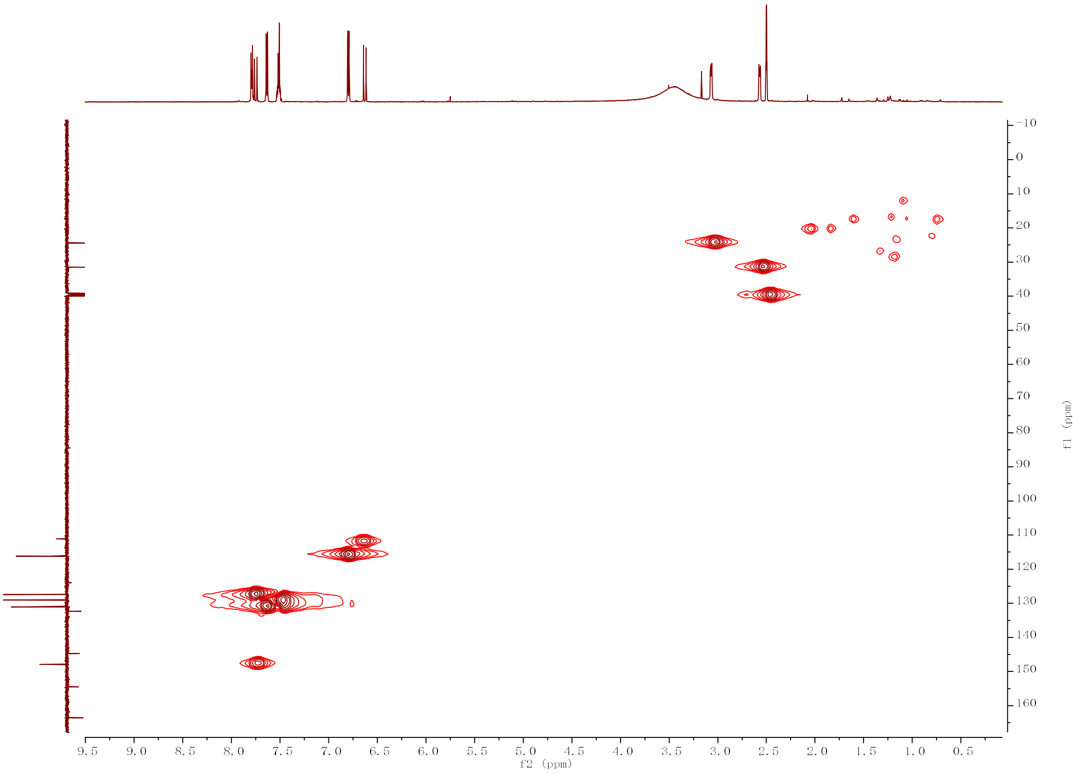

Figure S27. ^{1}H-^{1}H COSY spectrum of acremoketene A (**12**).

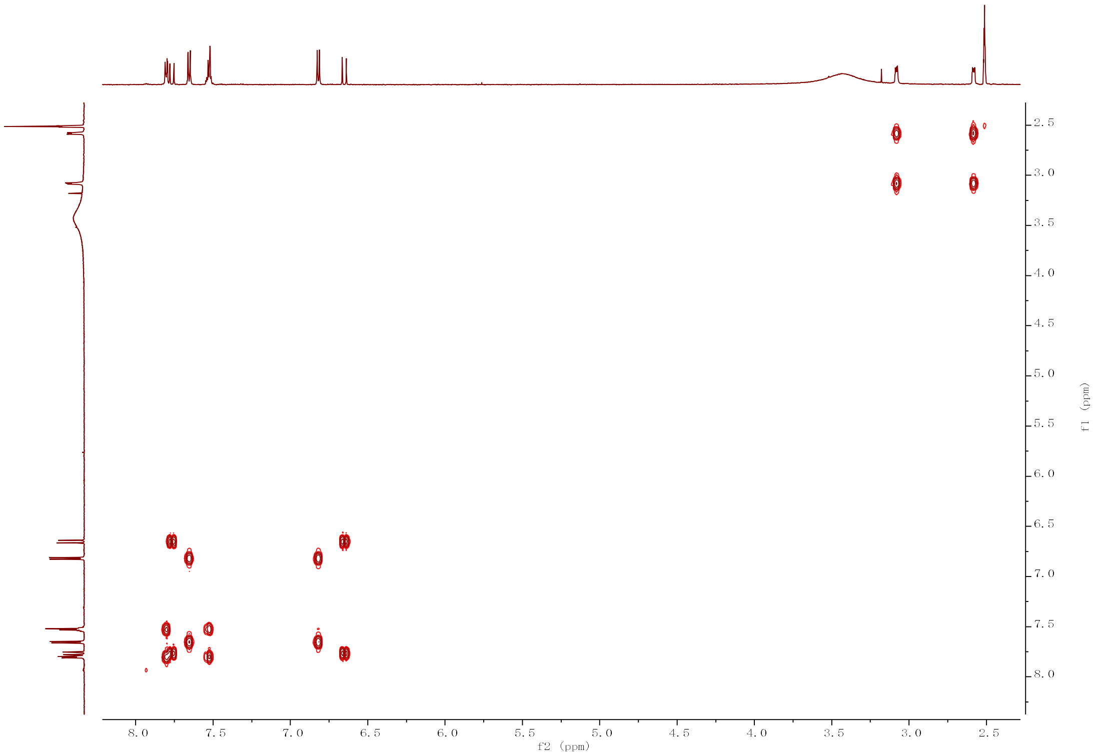

Figure S28. HMBC spectrum of acremoketene A (**12**).

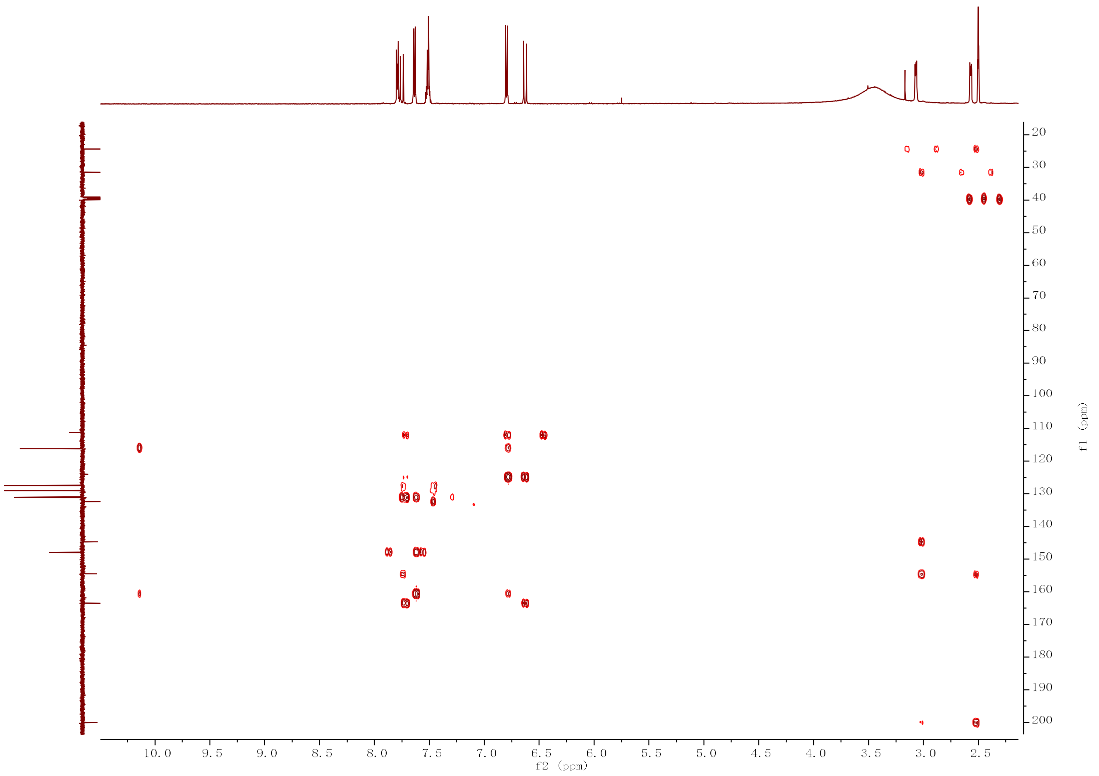

Figure S29. Optimized geometries of predominant conformers (weighting factors) for (2'*R*,6'*S*,7'*R*,11'*R*)-**1** subjected to calculations at the B3LYP/6-31g(d) level (above 2% population, from 128 conformers in total).

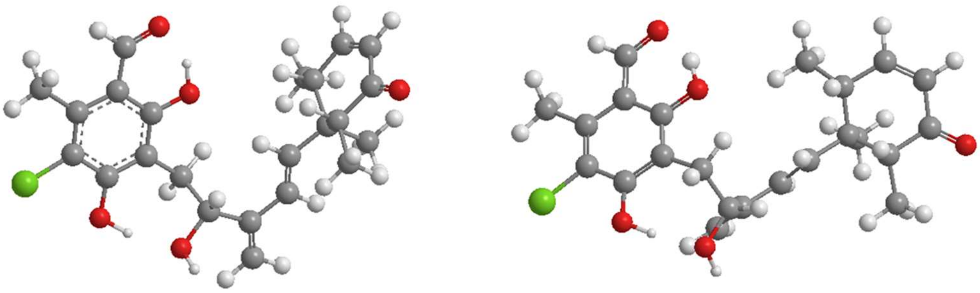

conf. A (96.4%) conf. B (3.3%)

Figure S30. Optimized geometries of predominant conformers (weighting factors) for (2'*S*,6'*S*,7'*R*,11'*R*)-**2** subjected to calculations at the B3LYP/6-31g(d) level (above 2% population, from 129 conformers in total).

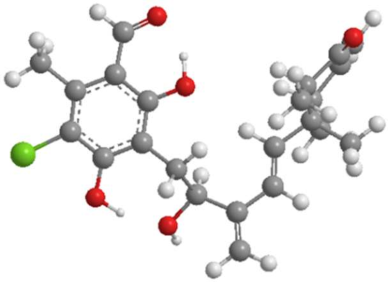

conf. A (90.0%)

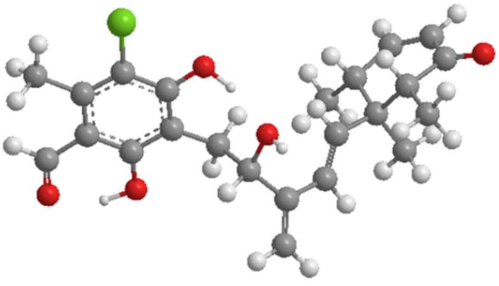

conf. B (9.2%)

Figure S31. Optimized geometries of predominant conformers (weighting factors) for (9*S*,10*S*,12*R*,15*R*)-**7** subjected to calculations at the B3LYP/6-31g(d) level (above 1% population, from 29 conformers in total).

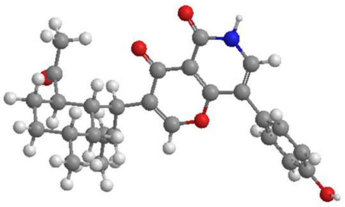

conf. A (27.2%)

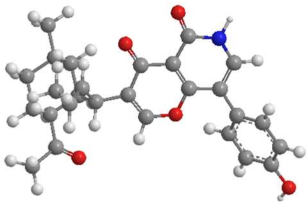

conf. B (16.3%)

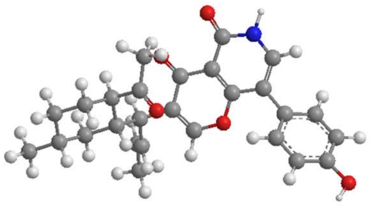

conf. C (16.1%)

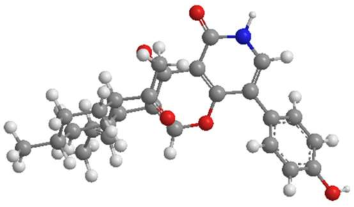

conf. D (15.6%)

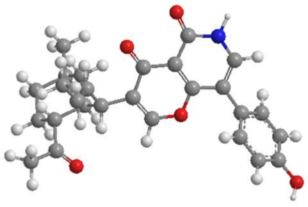

conf. E (11.3%)

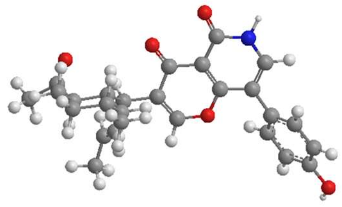

conf. F (3.2%)

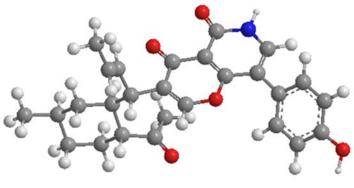

conf. G (2.8%)

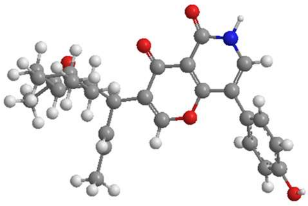

conf. H (2.7%)

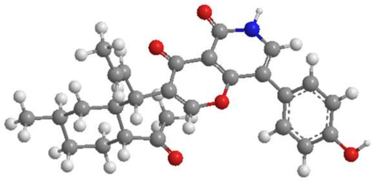

conf. I (2.1%)

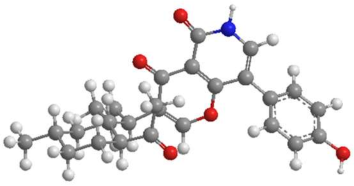

conf. J (2.0%)

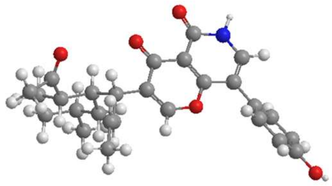

conf. K (1.9%)

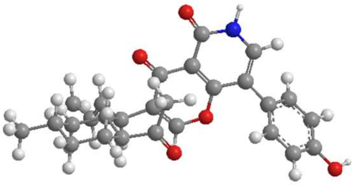

conf. L (1.5%)

Figure S32. Optimized geometries of predominant conformers (weighting factors) for (9*R*,10*R*,12*S*,15*S*)-**7** subjected to calculations at the B3LYP/6-31g(d) level (above 1% population, from 34 conformers in total).

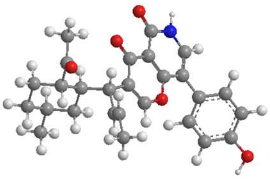
conf. A (28.8%)

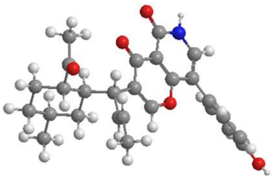
conf. B (21.0%)

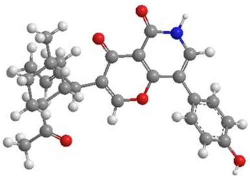
conf. C (11.5%)

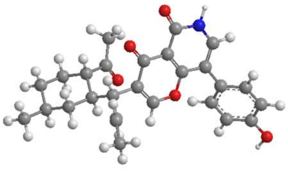
conf. D (11.4%)

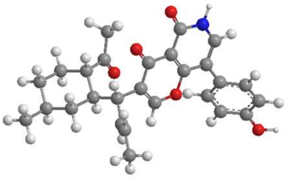
conf. E (11.1%)

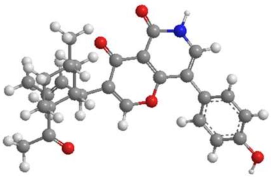
conf. F (8.0%)

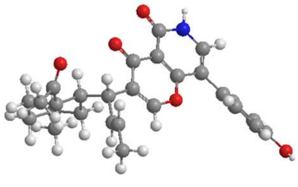
conf. G (2.3%)

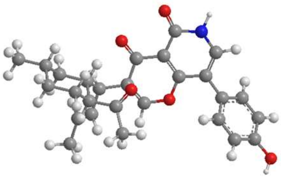
conf. H (1.9%)

Figure S33. Optimized geometries of predominant conformers (weighting factors) for (9*S**,10*R**,12*S**,15*S**)-**7** at the B3LYP/6-31g(d) level (above 2% population, from 25 conformers in total).

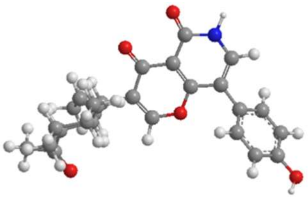
conf. A (34.9%)

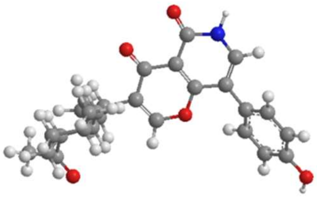
conf. B (20.0%)

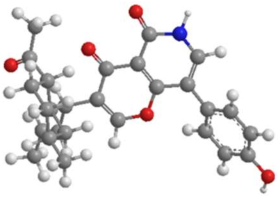
conf. C (17.2%)

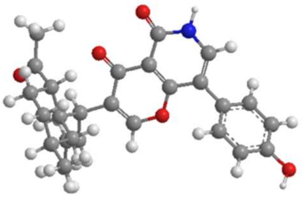
conf. D (11.7%)

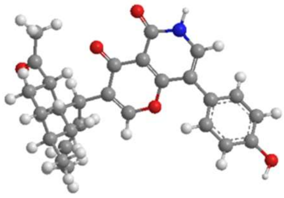
conf. E (4.2%)

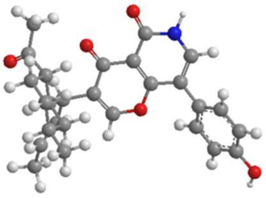
conf. F (3.8%)

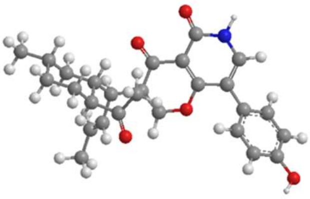
conf. G (2.5%)

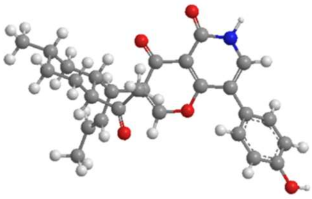
conf. H (2.2%)

Figure S34. DP4+ probability analysis of compound **7** (mPW1PW91/6-31+G(d,p) level).

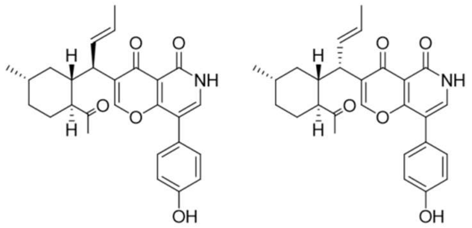

7a (isomer 1 in calculation) **7b** (isomer 2 in calculation)

Functional		Solvent?		Basis Set		Type of Data	
mPW1PW91		PCM		6-31+G(d,p)		Shielding Tensors	
		Isomer 1	Isomer 2	Isomer 3	Isomer 4	Isomer 5	Isomer 6
sDP4+ (H data)		99.58%	0.42%	–	–	–	–
sDP4+ (C data)		99.78%	0.22%	–	–	–	–
sDP4+ (all data)		100.00%	0.00%	–	–	–	–
uDP4+ (H data)		99.28%	0.72%	–	–	–	–
uDP4+ (C data)		99.93%	0.07%	–	–	–	–
uDP4+ (all data)		100.00%	0.00%	–	–	–	–
DP4+ (H data)		100.00%	0.00%	–	–	–	–
DP4+ (C data)		100.00%	0.00%	–	–	–	–
DP4+ (all data)		100.00%	0.00%	–	–	–	–
